# Supplementary material for: Testing approaches to sharing trial results with participants: The Show RESPECT cluster randomised, factorial, mixed methods trial
Source: PLoS Med. 2021 Oct 4;18(10):e1003798. doi: 10.1371/journal.pmed.1003798 (PMC8523080; doi:10.1371/journal.pmed.1003798)
Supplement: S1 Text — (DOCX) [file pmed.1003798.s001.docx]

# S1 Text: Topic guide for qualitative interviews with patients

### Introduction

- Thanks
  - For participating in ICON8
  - And for agreeing to be interviewed
- Who I am
  - I’m a researcher working at the MRC Clinical Trials Unit at UCL. We run large clinical trials on cancer and infectious diseases, including the ICON8 trial. I’ve been working at the Unit for 8 years, and my focus is on how we communicate the results of our trials, and how we involve patients as partners in our research.
- Go through participant info sheet
- Aim of study – to help us find the best ways to communicate results to people in research studies
  - Why I want to talk to her
  - Confidentiality and anonymity
  - Recording
  - Any questions?
- Go through consent form
- Interested in your views and experiences - no right or wrong answers.
- We can stop at any time, or take a break, or skip questions you don’t want to answer

### Health information sources

- When you want health information, where do you usually go for it?
  - Why?
  - Any other sources?
  - (Probe re. whether they use websites / ask people / look at medical journals for information)
- Do you usually find what you want to know?
- How would you describe your confidence at using the internet to find out information?
- Is health research and medical science something you’re interested in?

### Experience of being part of ICON8

*Remind participant about what ICON8 was about*

- How long have you been taking part in the ICON8 trial for?
- Why did you decide to take part?
- How would you describe your experience of being part of ICON8?
  - Do you feel your participation is valued by your medical team?
- When you joined ICON8, did you think you would want to know the results of the trial when they were available?
- How good do you think your ICON8 doctors and nurses are at keeping you informed about your treatment and health?
  - Do they explain things well for you?
  - Have you had any problems understanding the medical information they give you?
  - Do you feel comfortable asking them questions about your health and treatment?
- How good do you think your ICON8 doctors and nurses are at keeping you informed about the research study?
  - Do they explain things well for you?
  - Have you had any problems with understanding the information you’ve been given about the study?
  - Do you feel comfortable asking them questions?
  - Do you get frequent enough updates about the study?

### Experience of finding out the ICON8 results

*Now we’re going to focus on your experience of finding out the overall ICON8 results (so not your individual test results, but the overall results of the study)*

- Do you remember receiving the patient update, telling you how to find out the results? [show copy]
  - If yes, what did you think when you received it?
    - How did you feel?
  - If no, let them look at the patient update to see if it jogs their memory
    - Would you like to find out the results in any of those ways?
  - Did you want to find out the results in the first place?
    - Did that change over time?
- Do you remember finding out the results of ICON8?
  - *If yes,* tell me about finding out the results of ICON8
    - How?
    - How was your health when you found out the overall results?

Reaction to finding out the results

- - - How did you feel emotionally about finding out the results?
      - Why?
      - *(Comprehension, content, respected / valued, pride, satisfaction, made a difference, disappointed, confused)*
    - Has your emotional reaction to the results changed over time?
      - If yes, in what way?
    - Do you regret finding out the overall study results, or are you glad you did?
      - If yes, Why?
- *[Look at website/printed summary/email together if either of these were how they found out the results]*
  - What do you think of it now you’ve seen it again?
  - What do you like or dislike about it? (prompt: look and feel, content, language, navigation, *use of diagrams, links to further support and information, video, faq section*)
  - Which bits did you look at? Was anything particularly interesting? [prompt more on this]
  - Were there particularly boring or irrelevant bits?
  - Was any of it upsetting?
  - What would you change or add?
  - Was any of it confusing or unclear?
  - What do you think of the layout and formatting?
- Were you offered any other ways of finding out the results?
  - *If yes,* Which?
    - Did you use any of those ways?
      - *[If yes]* which? Why? Tell me about it.
      - *[if not:]* Why did you decide not to use those ways?
- Would you prefer to be able to find out the results in a different way?
  - *If so, how? Why?*
- If the results had been different, do you think that would change how you would prefer to have the results communicated to you?
  - *What if having weekly chemotherapy was better?*
  - *What if having three weekly chemotherapy was better?*

### Interpretation of the results

- What do you think the ICON8 results mean?
  - *Interesting? Important?*
  - *Do you think it will help other patients?*
- Did you discuss the results with anyone?
  - *If so, who?*
    - *Doctor / nurse?*
    - *Friends / family?*
  - *What did you talk about?*
- Did you have any questions about the results?
- Did you want any further information or support?
  - If so, what info or support did you want?
  - Did you seek further info or support?
    - If yes, did you get further info or support?
      - *If yes,* who or where from?
- How do the results compare to your own experience in ICON8?
- How did finding out the overall results make you feel about the group of the study you were in?

### Other ways of finding out the results

Some of the other women taking part in the ICON8 trial found out the results from this webpage [show other website – give time to look at]

- What do you think of this way of finding out the ICON8 results?
- Would you have liked the option to find out this way?
  - *Why?*
- How do you think researchers could improve it?
- What do you think of the layout?
- What do you think of the content?

*For those looking at enhanced webpage:*

- Do you think the video is helpful?
  - *For you?*
  - *For others?*
- Do you think the links to further information and support are helpful?
  - *For you?*
  - *For others?*
- Do you think the diagrams are helpful?
  - *For you?*
  - *For others?*
- Do you think being able to send in a question and have it answered on the webpage is helpful?
  - *For you?*
  - *For others?*

*For women who didn’t receive the printed summary:*

Some of the other women taking part in the ICON8 trial were sent this printed summary through the post [give copy and time to look at]

- What do you think of this way of finding out the ICON8 results?
- Would you have liked to find out this way?
  - *Why?*
- What do you think about the format and layout?
- What do you think about the use of diagrams?
- What do you think about the wording?
- How do you think researchers could improve it?

*For women who weren’t offered the email list:*

Some of the other women taking part in the ICON8 trial were able to join an email list to receive the results by email. Here’s the email they were sent. [Give time to look at email]

- What do you think of this way of finding out the ICON8 results?
- Would you have liked to find out this way?
  - *Why?*
- What do you think about the format and layout?
- What do you think about the use of diagrams?
- What do you think about the wording?
- How do you think researchers could improve it?

### Sharing the results with others

- What do you think about us sharing the overall trial results with people who weren’t on the trial, but care for people on the trial?
  - Eg. relatives
  - Wider care team (eg. GP)
- What do you think we should do about sharing overall trial results where the person who was taking part in the trial has died?
  - Should we contact relatives to offer them results?
    - helpful for them to know what their loved one contributed to?
    - too upsetting?
    - How can we do it sensitively?

### Final thoughts

- What advice would you give to researchers in other trials on how to share the results with participants?
- Is there anything else you'd like to say about this topic?

### Thanks and wrap up

- Thank you
  - For your contribution to the ICON8 trial, and to this study
- This information will help us improve how we communicate results to trial participants
- Answer any questions they have about the ICON8 results, if these came up earlier
- Give contact details for further support if needed
- Give voucher
