# Supplementary material for: Testing approaches to sharing trial results with participants: The Show RESPECT cluster randomised, factorial, mixed methods trial
Source: PLoS Med. 2021 Oct 4;18(10):e1003798. doi: 10.1371/journal.pmed.1003798 (PMC8523080; doi:10.1371/journal.pmed.1003798)
Supplement: S2 Text — (DOCX) [file pmed.1003798.s002.docx]

# S2 Text: Further information on uptake of interventions

3 patients opted out of the Mailed Printed Summaries. Mailed Printed Summaries were sent to 89 participants, 62 (70%) of whom reported using them, 10 (11%) reported not using them, and data were missing for the remaining 17 (19%). 23/90 (26%) participants randomised to the Basic Webpage and 28/90 (31%) randomised to the Enhanced reported using the webpage. 29/90 (32%) participants randomised to the Basic Webpage and 28/90 (31%) randomised to the Enhanced Webpage reported not using the webpage. However, data were missing on this question from 38/90 (42%) randomised to the Basic Webpage and 34/90 (38%) randomised to the Enhanced, making these results hard to interpret. 60/72 participants for whom this data is missing reported having found out the results, of whom 51 were in the Mailed Printed Summary group. No participants signed up to the email list.
