# Supplementary material for: Testing approaches to sharing trial results with participants: The Show RESPECT cluster randomised, factorial, mixed methods trial
Source: PLoS Med. 2021 Oct 4;18(10):e1003798. doi: 10.1371/journal.pmed.1003798 (PMC8523080; doi:10.1371/journal.pmed.1003798)
Supplement: S3 Text — (DOCX) [file pmed.1003798.s003.docx]

# S3 Text: Further qualitative findings

## Qualitative findings around participants’ desire for trial results

Most of the participants interviewed had wanted to receive the results of the trial. For several, this interest went back to when they joined the trial. Participants’ motivation for finding out the ICON8 results varied, including wanting to know how they had been affected by trial participation, that they had contributed to something, and gaining closure. The former two motivations for finding out the trial results mirror the motivations for taking part in the ICON8 trial, which were around the perceived benefits of trial participation to themselves (such as access to potentially better treatment approaches, and increased monitoring) and benefits to other patients in the future.

One interviewed participant said she did not want to receive the ICON8 results as, although she herself was well, she did not want to find out that others in the trial had done less well. If, however, she had been on a trial for a less serious condition, she would have been interested in learning the results. Importantly, receiving the Patient Update Information Sheet saying results were available was not upsetting for this patient.

*"If everything is fine, and all the ladies on the trial are fine, I would want to read that. If I was going to read it, and I found out that a very high proportion were not well, I don’t want to see that. I’m probably putting my head in the clouds, or burying my head in the sand probably. But I’m very well at present, and I’m sure I’ll stay well, but … there are always the seeds of doubt… "* GSI01

## Other reasons for not wanting to know the results included worry, feeling like the results would not make any difference to their situation, and wanting to believe that the treatment they had was the best. Difficulties accessing or understanding the results were also reported as reasons for not wanting to know the results.

## Qualitative findings on whether the information told participants everything they wanted to know

In the interviews, most participants said that the results summaries they had received had told them everything they wanted to know. Some felt that including any extra information would make it harder for participants to understand or could deter people from reading it. Against this, some extra information items that a minority of participants would have liked to have been included in the results summaries were identified. These included more information on side-effects, overall survival, the demographics of the participants who took part in the trial, a more detailed breakdown of the results by subgroups, compliance with treatment, the study design, more information putting the results into the wider context, a personalised reminder of which group they had been in, and personalised information on how their individual results compared to the overall results.

## Qualitative findings on whether the information was understandable

Participants described the information as “clear”, “easy to understand” and not too “technical”. In the interviews, nearly all participants were able to give accurate summaries of what the results showed, and their implications. However, for some participants, it had taken them a while to reach that understanding.

*"Initially, when I first read it, I was thinking I’m really glad I had the ‘every three weeks’ [sic] because I thought the outcome would be better. But actually, when we looked at it again… I can see basically, it didn’t seem to make much difference whichever way the treatment was administered. It’s just the different side effects from different dosages."* DMI01

A minority of participants did say the results were difficult to understand. For some, this was because of the large amount of information provided in the results summaries. Others did not understand that a trial might not produce a clear 'winner', so were confused about the implications even while their understanding was accurate.

*"I got a bit confused to be quite honest, because I didn’t fully understand it. I came to the conclusion that the three different methods of administering the chemo didn’t produce a winner, if you like. I got a bit lost after that as to where you went from there, because to me there hadn’t been any great find; any big development."* GMI01

The information at the top of the Basic Webpage, including the full scientific title of the trial and registration number, was felt by some to be too complex for lay readers, but was important information for others. One patient, who received the results via the Mailed Printed Summary, said she would have preferred to read the results online, as she found the printed information difficult to understand.

## Qualitative data on patients’ emotional response to the results

In the qualitative interviews, nearly all participants talked about being “pleased” or “glad” to receive the results. For some participants, particularly those in the ICON8 control group (three-weekly chemotherapy), receiving the results gave them reassurance or relief, as it told them they had not missed out on a superior treatment. Receiving the results of the trial was a positive experience for some participants because it made them feel part of something “worthwhile” and gave them a sense of completion.

*“It makes it more worthwhile having done it. I think it doesn’t leave anything unfinished or in the air or doesn’t leave me wondering so that I know that it’s been completed and all these other women have taken part. It’s quite special really, I suppose, 1,500 women in the world from different countries and we’ve all been part of the same thing. It’s quite powerful stuff.”* DLI01

Both the qualitative and quantitative results agree that, for most participants receiving the results was a positive experience overall, but one that was tinged, for some, with more negative emotions as well. One patient spoke of feeling panic as she read the results. However, she did not mind going through the panic to get the results.

*"I think because when you first get some results like that, especially in my situation, you almost panic. You get the result but you panic. So, you read it and you’re panicking, although you can see the [overall] outcome’s good."* DMI01

Several participants (mostly but not exclusively from the ICON8 intervention arms) spoke of being disappointed that the results did not show a benefit from one of the new treatment schedules. That disappointment was tempered by the understanding that you need to do trials to find out whether or not things work.

*"I was a bit disappointed really, because I thought, well, gone to all this trouble, and that’s obviously not a crucial factor. But on the other hand, when you think about it, at least they’ve eliminated that as a potential factor. So, that’s the way it works, isn’t it, that’s what you have to do, you have a theory, and test it."* FLI01

None of the participants interviewed regretted taking part in the trial, or receiving the results, despite the disappointment of the results not showing a benefit. Nor did they say that they had found the results upsetting.

*"Upsetting isn’t a word I’d use because, no, it’s all interesting. I don’t know, it’s interesting really, isn’t it? As a patient, I suppose I’m looking at it almost forgetting that I’m one of them, so you kind of extract yourself reading it."* DLI01

However, the quantitative results revealed that some other participants had reported being upset by the results. It may be that those who had found the results upsetting were unwilling to be interviewed, in order to protect themselves from further upset, making it difficult to explore this further. One questionnaire respondent said that she had found some of the terminology used to explain the outcome measure upsetting, rather than the results themselves.

*"It was more the terminology that caused upset 'period of survival' implied that illness will come back rather than cured."* HLQ06

Some participants’ emotional reaction to the results changed between initially finding out the results, and the interview (usually several months), with the initial negative emotional reaction being softened.

*“I’m more objective now than I was when I first got the results. I was thinking, oh my God, how long have I got? And I read it again yesterday, only because I thought I must update myself before we talked about it. It’s not quite so daunting now.”* DMI01

## Qualitative findings on attitudes to the results

Participants described the results as “interesting” and “important”. Despite ICON8 not finding any benefits from the treatment regimens tested, participants still felt the ICON8 trial had been worthwhile, either through ruling out a potential treatment, or providing data that might eventually lead to something in the future.

*"The initial disappointment that I felt, it’s obviously softened with time. I still think that I’m pleased I took part, and I still think that some lessons may eventually come from this research in future. You know, something else might happen, and then this might become very relevant again. So I think it’s still all been very worthwhile."* BLI01
