# Supplementary material for: Testing approaches to sharing trial results with participants: The Show RESPECT cluster randomised, factorial, mixed methods trial
Source: PLoS Med. 2021 Oct 4;18(10):e1003798. doi: 10.1371/journal.pmed.1003798 (PMC8523080; doi:10.1371/journal.pmed.1003798)

# S1 Fig: Forest plot of satisfaction with how the results were shared, by subgroup

Forest plot showing the adjusted odds ratio for satisfaction with how the results were shared for each of the three randomisations, by sub-group.


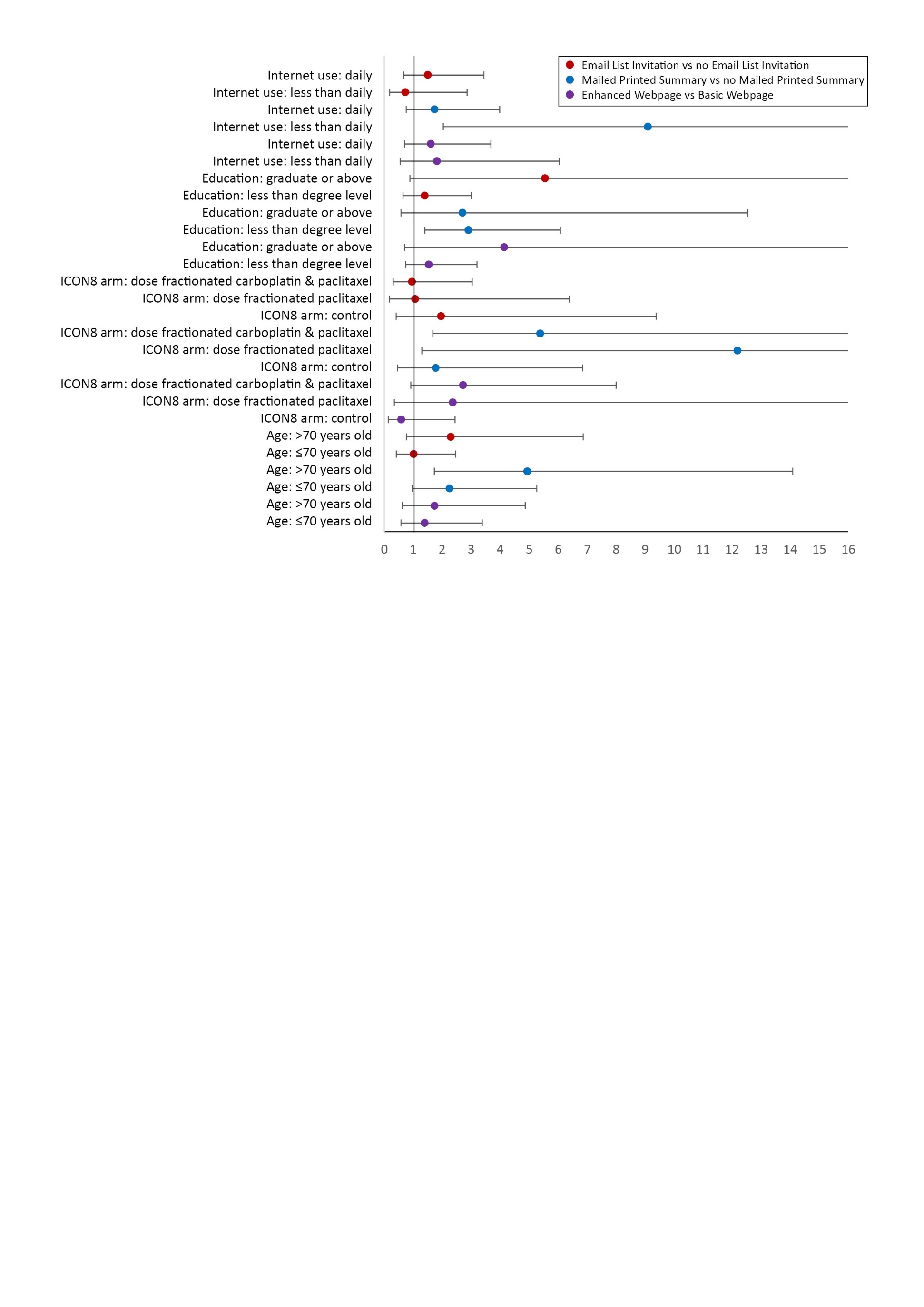

Supplement: S1 Fig — (DOCX) [file pmed.1003798.s004.docx]
