# Supplementary material for: Testing approaches to sharing trial results with participants: The Show RESPECT cluster randomised, factorial, mixed methods trial
Source: PLoS Med. 2021 Oct 4;18(10):e1003798. doi: 10.1371/journal.pmed.1003798 (PMC8523080; doi:10.1371/journal.pmed.1003798)
Supplement: S1 Appendix — (PDF) [file pmed.1003798.s005.pdf]

An international phase III randomised trial of dose-fractionated chemotherapy compared to standard three-weekly chemotherapy, following immediate primary surgery or as part of delayed primary surgery, for women with newly diagnosed epithelial ovarian, fallopian tube or primary peritoneal cancer

Introduction

We now have the first results from the ICON8 study. This information sheet contains details of what the next steps are for you and the study. It will also tell you how you can find out the results of the ICON8 study.

The ICON8 study is testing how best to give chemotherapy to women with ovarian cancer. It compared having chemotherapy every week to the current standard of having chemotherapy once every three weeks. It aimed to see if weekly chemotherapy is better at delaying or preventing the disease getting worse and improving how long women live for.

Women who agreed to take part in the ICON8 study were split into 3 groups, at random.

- 522 women were in group 1. They received standard chemotherapy, with two drugs (paclitaxel and carboplatin) given once every 3 weeks for 6 treatments (cycles). This took 18 weeks in total.
- 523 women were in group 2. They received the chemotherapy drug paclitaxel once a week, and the drug carboplatin once every 3 weeks for 6 cycles. This took 18 weeks in total.
- 521 women were in group 3. They received both paclitaxel and carboplatin once a week for 18 weeks.

Thank you

Thank you for taking part in the ICON8 study. You are helping us to answer important questions about how to treat women with ovarian cancer. This will help other women with ovarian cancer in the future.

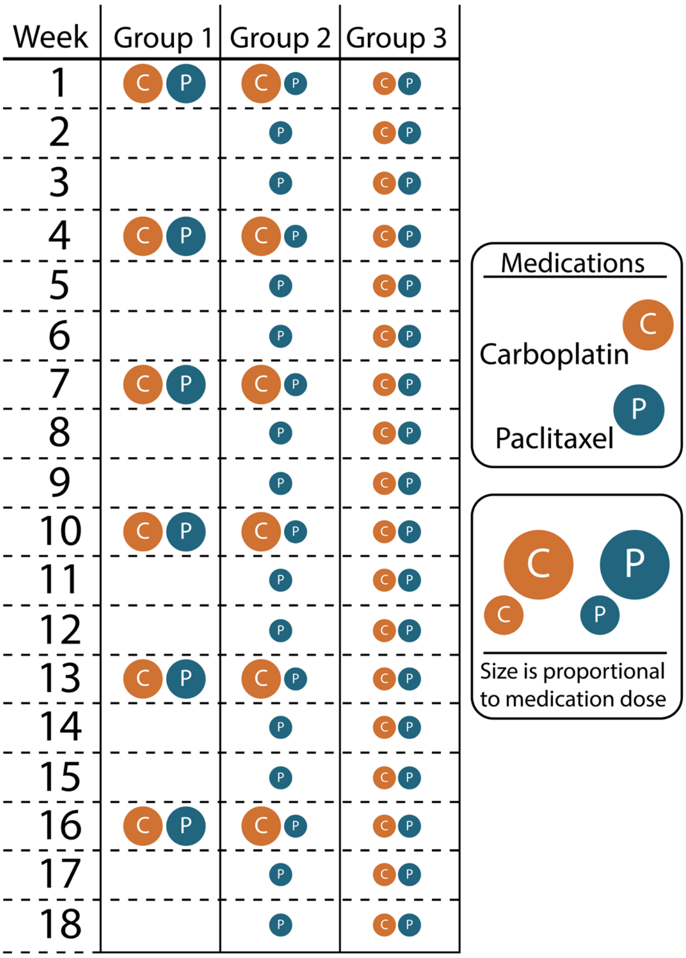

Reference numbers

IRAS ID: 11/LO/0043
ISRCTN: 10356387

What is happening now in the ICON8 study?

All the women in ICON8 have completed their study treatment. We are now in the 'follow-up' phase. This is where we keep track of how you are doing, but your current and future treatment is the same as patients who are not in the trial.

Your study doctors and nurses will continue to monitor how you are, as part of the trial. This will help us to answer questions about the long-term effect of weekly chemotherapy.

How can I report side-effects?

When you see your doctor or research nurse at each hospital visit they will ask you about any side-effects you have had. It is important that you tell your doctor or research nurse about any problems. We will monitor you closely for any possible side-effects and your doctor or nurse may suggest extra tests if he/she considers it appropriate.

What results will be available and when?

We now have results telling us about whether weekly chemotherapy delays ovarian cancer getting worse, compared to having chemotherapy once every three weeks.

We do not yet know whether weekly chemotherapy makes a difference to how long women live, on average, compared to having chemotherapy once every three weeks. We expect these long-term results to be ready sometime in 2019.

How can I find out the results of the research?

We have put a summary of the results on this webpage [insert URL], which you can visit if you want to find out the results.

We will post you a written summary of the results. If you **do not** want us to send you the results, please tell your research nurse or doctor within the next three weeks. If we do not hear from you, we will assume that you would like the results to be posted to you.

If you want us to email you a summary of the results, sign-up for our email list here [insert URL of sign-up form]

Will I be given any results about me as an individual?

Your doctor has already discussed the results of any tests or scans you have had with you when they became available. If you have any questions about these, please ask your doctor or research nurse.

Which group of the study was I in?

If you would like to be reminded about which group of the study you were in, please ask your doctor or research nurse.

If I have any questions, whom should I contact?

If you have any questions about the ICON8 study, please speak to your doctor or research nurse.

Further information

ICON8 study is registered with the ISRCTN registry. The registration number is 10356387. You can see more details about the trial http://www.isrctn.com/ISRCTN10356387

The ICON8 study was sponsored by the Medical Research Council. It was funded by Cancer Research UK.
