# Supplementary material for: Testing approaches to sharing trial results with participants: The Show RESPECT cluster randomised, factorial, mixed methods trial
Source: PLoS Med. 2021 Oct 4;18(10):e1003798. doi: 10.1371/journal.pmed.1003798 (PMC8523080; doi:10.1371/journal.pmed.1003798)
Supplement: S3 Appendix — (PDF) [file pmed.1003798.s007.pdf]

From: ICON8 Results
To: South, Annabelle
Subject: ICON8 results update December 2018
Date: 10 December 2018 11:21:47

View this email in your browser

Results of the ICON8 trial

Thank you

Thank you for taking part in the ICON8 trial. You have helped us to answer important questions about how to treat women with ovarian cancer. We need you to carry on attending clinic visits so we can find out important longer term results. This will help other women with ovarian cancer in the future.

This email describes the results of the study, including statistics about survival and side effects. If you have any questions about the trial and its results, or if this summary raises any other worries for you, please speak to your oncologist or research nurse.

We wrote this summary in May 2018. We will have more results from this study at a later stage. This summary only includes results from the ICON8

What's in this email?

Clink on the links below to skip straight to a section.

What was the ICON8 trial about?
Why was the ICON8 trial needed?
Who took part in the ICON8 trial?
How was the ICON8 trial carried out?
What did the ICON8 trial find?
How sure can we be about these results?
What do these results mean?
What difference will these results make?
Thank you
Further information
Any questions?
Support
Tell us what you think about this email

trial. Other studies may find different results.

What was the ICON8 trial about?

The ICON8 trial tested how best to treat ovarian cancer. It compared three ways of giving chemotherapy:

- Standard chemotherapy, giving both carboplatin and paclitaxel (sometimes also called Taxol) once every three weeks for a total of 18 weeks (Group 1)
- Weekly chemotherapy, giving carboplatin once every three weeks and paclitaxel once a week (at a lower dose) for a total of 18 weeks (Group 2)
- Weekly chemotherapy, giving both carboplatin and paclitaxel once a week (at a lower dose) for a total of 18 weeks (Group 3)

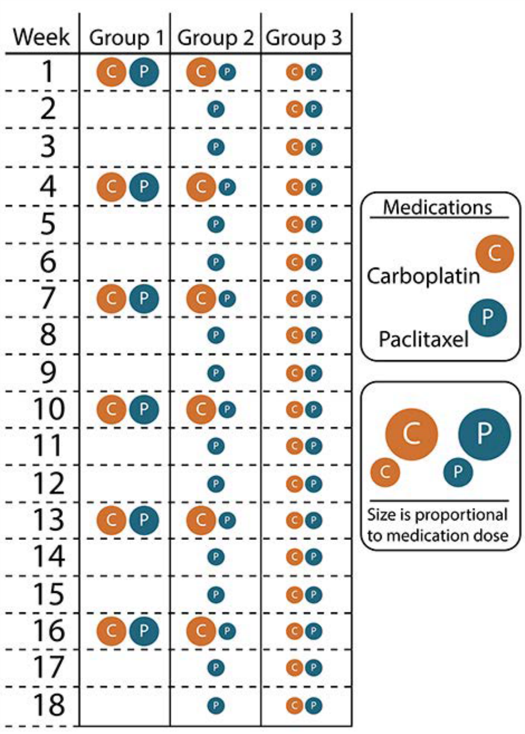

The aim of the study was to see if having chemotherapy every week rather than every three weeks could:

- delay (or prevent) the cancer coming back or getting worse
- improve how long women with ovarian cancer lived (we hope to find out these results in 2019)

Why was the ICON8 trial needed?

Ovarian cancer is usually treated by a combination of surgery and chemotherapy. Surgery is done to remove as much of the cancer as possible. The initial chemotherapy used for ovarian cancer usually involves two drugs, carboplatin and paclitaxel (sometimes also called Taxol). Chemotherapy might be started before or after surgery, depending on the extent of cancer. These drugs are recommended by international experts for treating ovarian cancer. They are referred to as "standard chemotherapy". This treatment is usually given six times, once every three weeks over 18 weeks.

A previous study in Japan suggested that giving chemotherapy more often than once every three weeks may also be effective. This type of treatment involves giving paclitaxel and/or carboplatin at a lower dose every week for 18 weeks during treatment, rather than a larger dose once every three weeks. We call this "weekly chemotherapy".

In this study we wanted to find out if weekly chemotherapy is better than standard chemotherapy in treating women with ovarian cancer. We also wanted to see if weekly chemotherapy causes more or fewer side-effects than standard chemotherapy. Although weekly chemotherapy involves more doses of chemotherapy than standard chemotherapy, the treatment course is the same length for both.

Who took part in the ICON8 trial?

People taking part in the ICON8 trial were:

- female and at least 18 years old
- diagnosed with stage Ic, II, III or IV ovarian cancer, fallopian tube cancer or primary peritoneal cancer
- well enough to be up and about for at least half the day
- starting treatment for ovarian cancer for the first time

The trial took place in almost 100 UK hospitals as well as hospitals in Korea, the Republic of Ireland, Mexico, Australia and New Zealand.

1566 women took part in the ICON8 trial. The average age of women who joined ICON8 was 62, ranging from 22 to 84 years old. Most women had advanced ovarian cancer (stage IIIc or stage IV).

How was the ICON8 trial carried out?

Women joined the ICON8 trial between June 2011 and November 2014.

People who agreed to take part in the trial were put into three groups.

- Group 1 (522 women): received standard chemotherapy, having both carboplatin and paclitaxel once every three weeks for a total of 18 weeks.
- Group 2 (523 women): received weekly chemotherapy, having carboplatin once every three weeks and paclitaxel once a week (at a lower dose) for a total of 18 weeks
- Group 3 (521 women): received weekly chemotherapy having both carboplatin

and paclitaxel once a week (at a lower dose) for a total of 18 weeks

Women in ICON8 could have surgery before or part way through their chemotherapy. Most women did have surgery.

So far, we have followed up how women were doing for at least 3 years. We wanted to see if having chemotherapy every week rather than every three weeks could delay (or prevent) the cancer coming back or getting worse, and improve how long women with ovarian cancer lived. We also looked at the side-effects women taking part in the study reported.

What did the ICON8 trial find?

The ICON8 trial found no difference in how long it was until the cancer came back or got worse for women who had weekly chemotherapy, compared to women who had three weekly chemotherapy.

On average, women who had chemotherapy every three weeks (Group 1) had around 24 months before their cancer came back or got worse. Women who had carboplatin every three weeks, and paclitaxel every week (Group 2) had around 25 months before their cancer came back or got worse, on average. Women who had carboplatin and paclitaxel every week (Group 3) also had around 25 months, on average, before their cancer came back or got worse. This difference is not big enough for us to be confident that having weekly chemotherapy is better than having chemotherapy once every three weeks. These results are averages. This means some women have done better, with the disease not coming back or getting worse, and others have had their disease come back or get worse sooner.

We found no evidence of any subgroups of women taking part in ICON8 benefitting from weekly chemotherapy compared to three-weekly chemotherapy. We looked at subgroups including stage of disease, and whether chemotherapy was started before or after surgery.

Many women in the study told us they had some side-effects. The main side-effects were:

- Having a low number of white blood cells and a fever
- Pins and needles, numbness, and/or pain, usually in your feet
- Severe anaemia (low numbers of red blood cells, or low levels of haemoglobin in the blood)

The difference in numbers of women having any severe side-effect, a low number of white blood cells and a fever, or pins and needles numbness and /or pain is not big enough for us to be sure that it was due to the different treatment approaches. The

difference in numbers who had severe anaemia is big enough for us to be confident that women in Group 2 were more likely to have severe anaemia than women in the other groups because of the treatment approach used in that group.

Women in Group 3 (who had weekly carboplatin) were more likely to have an allergic reaction to carboplatin than women who only had it once every three weeks (groups 1 and 2). These reactions were mostly mild.

Generally, women's quality of life improved during the trial. Women in Group 1 saw a faster improvement in quality of life than those in Group 2 or 3. But nine months after joining the trial, women's quality of life was similar in all three groups.

Weekly chemotherapy was safe to give, but did not work better than 3 weekly chemotherapy as a first treatment for ovarian cancer. We think carboplatin and paclitaxel every 3 weeks should still be the standard treatment.

How sure can we be about these results?

The ICON8 trial had a large number of women taking part in it. This means we can be confident that having chemotherapy every week does not delay or prevent the cancer coming back or getting worse compared to having chemotherapy once every three weeks.

We do not currently know whether having chemotherapy once a week rather than once every three weeks improves how long women with ovarian cancer live for. We will need to follow-up women in ICON8 for longer to answer this question.

These results differ from an earlier trial in Japan, which showed that weekly chemotherapy increased how long women lived for compared to those who had chemotherapy every three weeks. This difference may be due to genetic differences between Japanese women and women from Europe and other places.

What do these results mean?

What do these results mean for you?

These results do not affect how you should be treated in the future.
Please continue to come to your appointments with your study doctor, so we can keep track of how well you are. This will help us to find out if there are any differences between the groups in the longer term, and see if it improves how long women with ovarian cancer live.

What do these results mean for other people?

These results suggest that women like those in ICON8 with ovarian cancer are unlikely to benefit from having chemotherapy once a week rather than once every three weeks.
ICON8 did not include women who were so unwell they were confined to bed for more than half of every day, so we do not know if they apply to them.
Evidence from the earlier Japanese trial suggests that Japanese women may benefit from weekly rather than three-weekly chemotherapy.

What difference will these results make?

These results will not change the way that future patients are treated. But they help doctors to understand more about how chemotherapy should be given to women with ovarian cancer. This may help them find other, better ways to treat ovarian cancer in the future.

The ICON8 trial will continue to follow-up women to answer the longer term question on whether weekly chemotherapy improves how long women live for.

A follow-on trial is now running called the ICON8B trial. ICON8B is looking at whether weekly chemotherapy is better than three-weekly chemotherapy for women who are also receiving the drug bevacizumab (also known as Avastin) in addition to chemotherapy.

Thank you

Once again, thank you for takin part in the ICON8 trial. You are helping us to answer important questions about how to treat women with ovarian cancer. We hope that the results of this trial will help women with ovarian cancer in the future.

Further information

If you have any questions about the ICON8 trial, please speak to your doctor or research nurse.

Cancer Research UK has information about ICON8 on their website

The ICON8 trial is registered with the ISRCTN registry. The registration number is 10356387

The ICON8 trial was sponsored by the Medical Research Council. It was funded by Cancer Research UK.

Target Ovarian Cancer have some useful information and support guides on their website, as do Ovacome and Cancer Research UK

Any questions?

Do you have a question about the ICON8 trial and what it found? Submit your question, and we will try to post an answer to it in the next email.

If you have a question about your own health or individual results, please ask your doctor or research nurse, who will be able to help you.

Support

Target Ovarian Cancer have a Support Line where you can speak to a nurse advisor. You can call the Support Line on 020 7923 5475.

Ovacome also have a Support Service that offers information and emotional support to women, their families, friends and carers. You can call the Support Service on 0800 008 7054, text them on 07427 390504, or instant message them on their website.

To find a Support Group or Service near you, visit Ovacome's list of local Support Services.

My Ovacome is an online community for anyone affected by ovarian cancer. It is a safe, supportive space for women with ovarian cancer and their friends and families to share their experiences and offer each other encouragement, knowledge, understanding and friendship.

Target Ovarian Cancer also has information about other sources of support on their website

Tell us what you think about this email

We are trying to improve how we communicate trial results to people taking part in our trials. If you have any comments about this email, please tell us.

You are receiving this email because you opted in via our website.

Our mailing address is:

MRC CTU at UCL
PO Box 75361
London, WC1A 9PL
United Kingdom

Add us to your address book

You can **unsubscribe** from this list at any time by:

- sending an email with the word 'unsubscribe' in the subject line to email address or visiting http://www.mailinglists.ucl.ac.uk/mailman/options/mrcctu.icon8-
- results/ and entering your email address and clicking the 'unsubscribe' button.

This email was sent to a.south@ucl.ac.uk
why did I get this? unsubscribe from this list update subscription preferences
MRC CTU at UCL · PO Box 75361 · London, WC1A 9PL · United Kingdom
