# Supplementary material for: Testing approaches to sharing trial results with participants: The Show RESPECT cluster randomised, factorial, mixed methods trial
Source: PLoS Med. 2021 Oct 4;18(10):e1003798. doi: 10.1371/journal.pmed.1003798 (PMC8523080; doi:10.1371/journal.pmed.1003798)
Supplement: S4 Appendix — (PDF) [file pmed.1003798.s008.pdf]

Show RESPECT
Show RESults to Participants Engaged in Clinical Trials

A cluster randomised factorial trial of different modes of communicating results to participants of the ICON8 phase III ovarian cancer trial

Version: 3.0
Date: 20-Aug-2018

MRC CTU at UCL ID: 18/0261
ISRCTN #:

MREC #: 18/LO/1011

Authorised by:
Name: Annabelle South
Role: Chief Investigator
Signature:

Date:

Name: Andrew Copas
Role: Study Statistician
Signature:
Date

This protocol has been produced using MRC CTU at UCL Protocol Template version 6.0. The template, but not any study-specific content, is licensed under a Creative Commons Attribution 4.0 International License (https://creativecommons.org/licenses/by/4.0/). Use of the template in production of other protocols is allowed**,** but MRC CTU at UCL must be credited.

GENERAL INFORMATION

This document was constructed using the MRC CTU at UCL Protocol Template Version 6.0. The CTU endorses the Standard Protocol Items: Recommendations for Interventional Trials (SPIRIT) initiative. It describes the Show RESPECT study, coordinated by the Medical Research Council (MRC) Clinical Trials Unit (CTU) at University College London (UCL). Every care has been taken in drafting this protocol, but corrections or amendments may be necessary.

COMPLIANCE

The study will be conducted in compliance with the approved protocol, the Declaration of Helsinki, the principles of Good Clinical Practice (GCP), Commission Directive 2005/28/EC with the implementation in national legislation in the UK by Statutory Instrument 2004/1031 and subsequent amendments, the UK Data Protection Act (DPA number: Z6364106), and the National Health Service (NHS) UK Policy Framework for Health and Social Care Research.

SPONSOR

UCL is the trial Sponsor and has delegated responsibility for the overall management of the Show RESPECT study to the MRC CTU at UCL. Queries relating to UCL's sponsorship of this trial should be addressed to the Max Parmar, MRC CTU at UCL Director, MRC CTU at UCL, 90 High Holborn, London, WC1V 6LJ UK or via the study team.

FUNDING

This study is funded by the MRC Clinical Trials Unit at UCL, from core funds awarded by the Medical Research Council, grant number MC_UU_12023/24.

AUTHORISATIONS AND APPROVALS

This trial was approved by London – Chelsea Research Ethics Committee and is part of the Greater Manchester clinical research network portfolio.

TRIAL REGISTRATION

This trial will be registered with the International Standard Randomised Controlled Trials Register, where it is identified as ***[number to be added]***.

TRIAL ADMINISTRATION

Please direct all queries to mrcctu.showrespect@ucl.ac.uk in the first instance.

COORDINATING SITE

MRC Clinical Trials Unit at UCL<br>90 High Holborn, 2nd Floor London WC1V 6LJ	**Switchboard**:	020 7670 4700
	Email:	mrcctu.showrespect@ucl.ac.uk

MRC CTU AT UCL STAFF

Project Leader:	Annabelle South	Tel:	0207 670 4827
Co-Investigator:	Conor Tweed	Tel:	0207 670 4619
Statistician:	Andrew Copas	Tel:	0207 670 4888
Trial Manager:	Cara Purvis	Tel:	0207 670 4930
Clinical Reviewer:	Nalinie Joharatnam		
Data Manager:	TBC		
Database programmer:	Carlos Diaz-Montana		
Programme Lead:	Matt Sydes		

CHIEF INVESTIGATOR

Annabelle South<br>MRC Clinical Trials Unit at UCL<br>Institute of Clinical Trials & Methodology<br>90 High Holborn<br>London<br>WC1V 6LJ<br>UK	Tel:<br><br>Email:	0207 670 4827<br><br>**a.south@ucl.ac.uk**

COLLABORATORS

ICON8 team: Babasola Popoola, Liz James, Rick Kaplan, Adrian Cook	MRC CTU at UCL
Barbara Bierer	Harvard Medical School
Katie Gillies	University of Aberdeen
Talia Isaacs	University College London
Amanda Hunn	Health Research Authority
Katie Scott	Cancer Research UK
Claire Snowdon	London School of Hygiene and Tropical Medicine
Eva Burnett	Patient and Public Involvement contributor
Will Cragg	University of Leeds

NB: throughout this document, “MRC CTU at UCL” is generally abbreviated to “CTU”.

SUMMARY OF TRIAL

SUMMARY INFORMATION TYPE	SUMMARY DETAILS
**[Acronym or short title]**	Show RESPECT
**Long Title of Trial**	Show RESults to Participants Engaged in Clinical Trials: A cluster randomised factorial trial of different modes of communicating results to participants of the ICON8 phase III ovarian cancer trial
**Version**	3.0
**Date**	20-August-2018
**ISRCTN #**	*[Insert after Registration]*
**MREC #**	18/LO/1011
**Study Design**	Show RESPECT is a 2 by 2 by 2 factorial cluster randomised controlled trial. Each cluster is an ICON8 site. The interventions sites will be randomised between are:<br>▪ Basic webpage vs enhanced webpage<br>▪ No printed summary vs printed summary posted to participant<br>▪ No email list invitation vs invitation to join email list<br>Quantitative data will be collected from all ICON8 sites in England, Scotland, Wales and Northern Ireland. Quantitative data will be collected from a pre-specified subset of trial participants, and from site staff at all included sites, and CTU staff involved in the study.
**Setting**	ICON8 sites in England, Scotland, Wales and Northern Ireland
**Type of Participants to be Studied**	Adults taking part in the ICON8 trial at sites in England, Scotland, Wales and Northern Ireland.
**Sponsor**	University College London
**Interventions to be Compared**	▪ Basic webpage vs enhanced webpage<br>▪ No printed summary vs printed summary posted to participant<br>▪ No email list invitation vs invitation to join email list<br>The factorial design means sites will be randomised to receive a combination of these interventions/controls.
**Study Hypothesis**	The study hypothesis is that each of these interventions is superior to the relevant control.
**Primary Outcome Measure(s)**	Participant’s reported satisfaction with how the results were communicated to them (using a 5 point Likert scale)
**Secondary Outcome Measure(s)**	Secondary effectiveness outcomes (collected from

SUMMARY INFORMATION TYPE	SUMMARY DETAILS
	participants):<br>▪ Whether the information about the trial results told participants everything they wanted to know<br>▪ Ease of understanding the results<br>▪ How upsetting participants found the results?<br>▪ Willingness to take part in another trial in the future<br>▪ Likelihood of recommending taking part in a clinical trial to friends and family<br>▪ Whether participants feel glad to have found out the results<br>▪ Participant regret at finding out the results<br><br>Secondary process outcomes (collected from participants):<br>▪ Reported uptake of the intervention(s) offered<br>▪ Proportion of participants who wanted to find out the results who reported finding out the results<br>▪ Proportion of participants who did not want to find out the results who reported finding out<br>▪ Ease of finding out the results<br>▪ Would they prefer to have been given the opportunity to find out the results in a different way? If so, how and why?<br>▪ If they found out the results in several ways, which did they prefer and why?<br><br>Secondary process outcomes (collected from site staff):<br>▪ Any concerns with the interventions or process<br>▪ Time taken for each of the interventions (and by whom)<br>▪ Who delivered the interventions<br>▪ Challenges faced implementing the interventions<br>▪ Number of queries received following results being communicated<br>▪ Costs incurred by the site in implementing the interventions<br>▪ What would they like to do differently for the next trial they are involved in communicating results for?<br>▪ What proportion of patients did the Patient Update Information Sheet go out to? (Collected via log)<br>▪ For sites in the Printed Summary arm, what proportion of patients did the printed summary get posted to? (Collected via log)

SUMMARY INFORMATION TYPE	SUMMARY DETAILS
	▪ For sites in the Printed Summary arm, how participants many opted out? (Collected via log)<br><br>Other secondary outcomes collected from site staff:<br>▪ Site staff's preferred method of communicating the results to participants<br>▪ Do they think any of the interventions they were allocated to should become standard practice for trials they are involved in?<br>▪ Would they prefer to have given participants a different way to find out the results? If so, what and why?<br><br>Secondary process outcomes (collected from CTU staff/data):<br>▪ Any concerns with the process<br>▪ Time taken at CTU for each of the interventions<br>▪ Challenges faced implementing the interventions<br>▪ Number of queries received from participants or sites about the interventions or results<br>▪ Costs incurred by the CTU in implementing the interventions<br>▪ What would they like to do differently for the next trial they are involved in communicating results for?<br>▪ Uptake of basic and enhanced webpages, by site (from analytics data)<br>▪ Sign-ups to email list<br><br>Other secondary outcomes collected from CTU staff:<br>▪ CTU staff's preferred method of communicating the results to participants<br>▪ Concerns with the interventions<br>▪ Would they prefer to have given participants a different way to find out the results? If so, what and why?
**Randomisation**	Sites will be randomised on a 1:1 ratio for each of the factorial randomisations.
**Number of Participants to be Studied**	c.255 ICON8 participants from at least 37 sites
**Duration**	1 year
**Funder**	MRC
**Chief Investigator**	Annabelle South

TRIAL SCHEMA

Figure 1: Trial Entry, Randomisation and Treatment

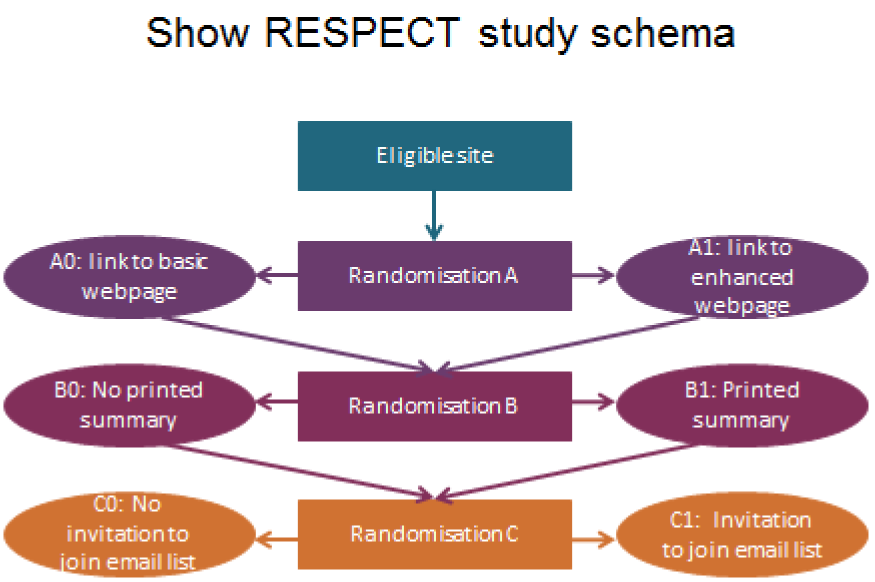

TRIAL ASSESSMENT SCHEDULE

Table 1: ICON8 Participant Trial Assessment Schedule

**Timeline:** / **Action**	As soon as possible following receipt of all appropriate approvals (for each site) ('baseline')	3 weeks after baseline	1 month after first site is randomised to this intervention	1 month after administration of last intervention at each site	2 months after administration of last intervention at each site	For all participants, we have data or confirmed unavailability of data (aim: within 6 months)
Distribute Patient Update Information Sheets (with links to basic or enhanced webpage) to sites for sending to participants	X					
Distribute printed results summary to sites for sending to participants		X				
Distribute results email to participants who have signed up to the mailing list			X			
Start of data collection				X		
Start of qualitative data collection					X	
End of data collection						X

LAY SUMMARY

BACKGROUND

We know that many people who take part in clinical trials want to be able to find out the results of their trial. We also know that, in many cases, this does not happen. It can be difficult for trial teams to provide the results to the people who took part. This is for a number of reasons, including:

- Practical challenges such as losing touch with people
- Lack of time and/or money
- Worry about upsetting people
- Difficulties explaining complex results
- Protecting the privacy of people taking part in trials
- Data protection laws
- Keeping track of people's wishes on whether or not to be told results

There is little evidence on how best to feedback results to the people who took part in the trial, either from a practical perspective, or on what people taking part in a trial prefer. The evidence that we do have is weak. This means that people running trials do not know how best to do it.

WHAT THE STUDY IS TESTING

The Show RESPECT study is trying to find practical ways to share the results of clinical trials with the people taking part in it. It is doing this by testing several different approaches within a large ovarian cancer trial (the ICON8 trial). Each hospital that is taking part in ICON8 in the UK will be allocated at random to share the results of the study in one or more of the following ways:

- Giving people taking part in the trial a link to a basic webpage that contains a simple summary of the results
- Giving people taking part in the trial a link to an 'enhanced' webpage that contains a simple summary of the results, links to further information, a short video of a doctor explaining the results, and a 'frequently asked questions' section that answers questions people send in
- Giving people taking part in the trial a simple printed summary of the results
- Inviting people taking part in the trial to join an email list, where a summary of results and updates will be sent out

WHAT INFORMATION WE WILL COLLECT

We will collect information from people taking part in the trial on how satisfied they are with how the results were communicated to them, including whether they found out the results, how easy it was to understand, and whether it told them everything they wanted to know.
We will also collect information from research nurses on how easy it was to share the results using these methods, how much time it took, and what they think of them.
We will also carry out interviews with a small number of people taking part in the trial and research nurses to explore their experience and views on this in more detail.

WHAT WE HOPE THIS STUDY WILL ACHIEVE

We hope this study will tell us which approaches to sharing results with people taking part in ovarian cancer trials are practical for trials to do, and are most satisfactory for the people taking part. This

will help us decide how best to do it in similar trials in the future. We hope this will improve people's experience of being part of a trial.

CONTENTS

GENERAL INFORMATION II

SUMMARY OF TRIAL V

TRIAL SCHEMA VIII

TRIAL ASSESSMENT SCHEDULE IX

LAY SUMMARY X

CONTENTS XII

ABBREVIATIONS XV

1 BACKGROUND 1

2 SELECTION OF TRIALS, SITES AND PATIENTS 2
2.1 SELECTION OF TRIALS 2
2.2 SELECTION OF SITES 2
2.3 PATIENT INCLUSION CRITERIA 2
2.4 PATIENT EXCLUSION CRITERIA 2
2.5 NUMBER OF PATIENTS 2
2.6 CONSENT 3

3 REGISTRATION & RANDOMISATION 4
3.1 RANDOMISATION PRACTICALITIES 4

4 INTERVENTIONS 5
4.1 INTRODUCTION 5
4.2 LINK TO BASIC WEBPAGE 7
4.3 LINK TO ENHANCED WEBPAGE 7
4.4 PRINTED SUMMARY 8
4.5 INVITATION TO JOIN EMAIL LIST 9
4.6 COMPLIANCE & ADHERENCE 9

5 QUANTITATIVE AND QUALITATIVE DATA COLLECTION 11
5.1 QUANTITATIVE DATA COLLECTION 12
5.1.1 ICON8 Participants 12
5.1.2 ICON8 site staff 13
5.1.3 MRC CTU trial team staff 13
5.2 QUALITATIVE DATA COLLECTION 13
5.2.1 Qualitative data collection from participants 13
5.2.2 Qualitative Data Collection from Site Staff 14
5.2.3 Analysis of qualitative data 15
5.3 PARTICIPANT MANAGEMENT 15

6 QUALITY ASSURANCE & CONTROL 16
6.1 RISK ASSESSMENT 16
6.2 MONITORING PROCESSES 16
6.3 ADVERSE EVENTS 16

7 STATISTICAL CONSIDERATIONS 18
7.1 METHOD OF RANDOMISATION 18
7.2 OUTCOME MEASURES 18
7.2.1 Primary outcome measure 18
7.2.2 Secondary effectiveness outcomes (collected from participants) 18
7.2.3 Secondary process outcomes (collected from participants) 19
7.2.4 Secondary process outcomes (collected from site staff): 19
7.2.5 Other secondary outcomes collected from Site Staff 19
7.2.6 Secondary process outcomes (collected from CTU staff/data): 20
7.2.7 Other secondary outcomes collected from CTU staff 20
7.3 SAMPLE SIZE 20
7.4 INTERIM MONITORING & ANALYSES 21
7.5 ANALYSIS PLAN (BRIEF) 21

8 REGULATORY & ETHICAL ISSUES 22
8.1 COMPLIANCE 22
8.1.1 Regulatory Compliance 22
8.1.2 Data Collection & Retention 22
8.2 ETHICAL CONDUCT 22
8.2.1 Ethical Considerations 22
8.2.2 Favourable Ethical Opinion 23
8.3 OTHER APPROVALS 23
8.4 STUDY CLOSURE 23

9 INDEMNITY 24

10 FINANCE 25

11 OVERSIGHT & TRIAL COMMITTEES 26
11.1 STUDY MANAGEMENT GROUP (SMG) 26
11.2 STUDY STEERING GROUP 26

12 PATIENT AND PUBLIC INVOLVEMENT 27
12.1 POTENTIAL IMPACT OF PPI 27
12.2 IDENTIFYING PPI CONTRIBUTORS 27
12.3 PROTOCOL DESIGN AND STUDY SETUP 27
12.4 PPI IN THE ONGOING RUNNING OF STUDY 28
12.5 INTERPRETING AND PLANNING DISSEMINATION OF STUDY RESULTS 28

13 PUBLICATION AND DISSEMINATION OF RESULTS 29

14 DATA SHARING 31

15 PROTOCOL AMENDMENTS 32

16 REFERENCES 36

ABBREVIATIONS

ABBREVIATION	EXPANSION
AE	Adverse event
CI	Chief Investigator
CISCRP	Center for Information and Study on Clinical Research Participation
CRF	Case Report Form
CRN	Clinical Research Network
CRUK	Cancer Research UK
CTA	Clinical Trials Authorisation
CTU	*See MRC CTU at UCL*
DM	Data Manager
DPA	(UK) Data Protection Act
EU	European Union
EudraCT	European Union Drug Regulatory Agency Clinical Trial
GCP	Good Clinical Practice
HRA	Health Research Authority
ICH	International Conference on Harmonisation of Technical Requirements for Registration of Pharmaceuticals for Human Use
ICON8	International Collaborative Ovarian Neoplasm 8 trial
IRAS	Integrated Research Application System
ISRCTN	International Standard Randomised Controlled Trial Number
ITT	Intention-to-treat
MRC	Medical Research Council
MRC CTU at UCL	Medical Research Council Clinical Trials Unit at University College London (also generally abbreviated to “CTU”)
MRCT	Multi-regional Clinical Trials Center, Harvard
NIHR	National Institute of Health Research
PPI	Patient and public involvement

ABBREVIATION	EXPANSION
QA	Quality Assurance
QC	Quality Control
QMAG	Quality Management Advisory Group
RCT	Randomised controlled trial
REC	Research Ethics Committee
RGC	Research Governance Committee
SAP	Statistical Analysis Plan
SAR	Serious adverse reaction
SMG	Study Management Group
SSA	Site-specific approval
SSG	Study Steering Group
TM	Trial Manager
TMF	Trial Master File
URL	Uniform Resource Locator

1 BACKGROUND

There is clear evidence that the majority of trial participants want to be informed of aggregate trial results [1-6]. Sharing results with participants demonstrates respect for their contribution, with some suggestion that it may increase the likelihood of participants taking part in future medical research, or recommending taking part in trials to others [1, 7-9]. Ethics committees and clinicians generally support the idea of sharing results with participants, even if they are not yet doing it in practice [10-13]. Many participants are not informed of the results of trials they contributed to [10, 11, 14]. The evidence on how best to do it is weak, and the guidelines that exist are largely based upon opinion rather than evidence.

The literature includes reports of various approaches to communicating results to participants, the most common being:

- Written summaries or letters sent by post
- Face-to-face individual meetings with clinicians
- Individual telephone calls
- Teleconferences
- Group meetings between patients and local trial staff

Most of the evidence is based on surveys of participants prospectively asking how they would prefer to be informed, or retrospectively asking whether an approach that was used is acceptable, rather than comparing outcomes from different approaches. Low response rates to surveys are also a limitation in some of the studies.

Most of the studies that have looked at this question report posted summaries as a preferred or acceptable approach for most participants [1, 2, 5, 6, 8, 14-18]. Some of these reported that participants had different preferences depending on whether they were in the arm that had the inferior treatment, with face-to-face conversations being preferred for this [5, 16]. Few studies to date have investigated the use of online methods such as websites, email, web chat or social media. These may offer practical advantages in terms of cost, reducing the burden on sites, and enabling participants to opt-in, but may not be appropriate for all patient groups. A number of pharmaceutical companies have recently developed online portals for participants to access trial results, although there are no published evaluations of these. The EU are also developing a portal that trialists will be required to post a lay summary of their results to, as part of the new EU Clinical Trial regulation.

Only one study identified in the literature review compared outcomes from different methods of communication. Dorsey et al. compared communicating results to participants in a Huntington's Disease trial by media release on a website, individual telephone call or teleconference [18]. They report participant satisfaction was highest with individual telephone calls, followed closely by a conference call. However, they did not assess sending written summaries to participants, and do not report on the resource implications of these different approaches.

More evidence is needed to inform trialists about which approaches (or combinations of approaches) are best in terms of participant satisfaction with how the results were communicated, and feasibility. There may be setting or disease-specific factors that influence participant satisfaction that influence this, so it is likely that both quantitative and qualitative evidence is needed to understand these issues.

2 SELECTION OF TRIALS, SITES AND PATIENTS

2.1 SELECTION OF TRIALS

To be considered for the Show RESPECT study, trials must meet the following criteria:

- phase III randomised controlled trials
- have some UK sites
- be due to publish primary or important trial results in the next year

The ICON8 trial meets these criteria, and will be the first trial in which the Show RESPECT study takes place. The intention is, following successful completion of the Show RESPECT study within the ICON8 trial, to expand the Show RESPECT study into several different trials. The protocol will be amended as required to reflect any future expansion of trials or sites in which this study will take place.

2.2 SELECTION OF SITES

The Show RESPECT study (referred to as 'the study') will take place in all sites that are part of the ICON8 trial (referred to as 'the trial') in England, Scotland, Wales and Northern Ireland, unless they opt out of the Show RESPECT study.

2.3 PATIENT INCLUSION CRITERIA

1. Participant in the ICON8 trial
2. Currently being followed up at an ICON8 trial site in England, Scotland, Wales and Northern Ireland
3. Aged 18 years or older

2.4 PATIENT EXCLUSION CRITERIA

1. Participant has previously informed their site that they do not wish to attend any further visits in relation to the ICON8 trial, or provide any further data (sometimes referred to as 'withdrawal of consent'); participants who previously stopped ICON8 trial treatment earlier than expected but continue in ICON8 follow-up will not be excluded, nor will participants who have reduced follow-up arrangements but still contribute data to the ICON8 trial.
2. Lost to follow-up from the ICON8 trial
3. Site staff consider the patient to be too unwell to be contacted about this study

2.5 NUMBER OF PATIENTS

The maximum number of patients who can take part in this study is the total number enrolled in the ICON8 trial at sites in England, Scotland, Wales and Northern Ireland, alive at the time results become available. All these participants will be offered the Show RESPECT interventions (according to the randomisation of their site). Data will be collected from a subset of these patients (see **Section 5**, Data Collection and **Section 7**, Statistical Considerations for further details).

2.6 CONSENT

No informed consent is required for the study interventions as a whole, as we would expect trial results to be shared with participants regardless of this study. Randomisation is site level (each site being a cluster), rather than individual, so individuals will not have the opportunity to opt out of randomisation. In addition, most of the interventions offered are by nature opt-in, i.e. trial participants can choose whether or not to visit a webpage, or join an email list.

We will provide participants and site staff who are sent the quantitative questionnaire with information about why we are collecting the study data. This information will be integrated into the data collection tool and will include explanations of:

- What the data collected will be used for
- How data will be stored
- Confirmation that provision of data for the study is entirely voluntary

Participants in Show RESPECT are not placed at any significant risk through being randomised to receive information about ICON8 trial results in different ways, especially as there is no strong evidence to recommend one approach above others. In order to avoid overloading participants with information (and likely reducing the completion rate as a result) we will therefore keep participant information short and focus on key information only.

In line with the HRA's guidance on proportionate approaches to informed consent for self-administered questionnaire-based research, we will not ask for a signature to confirm participants have read and understood the information; instead, completion and return of the questionnaire will be taken to indicate consent to use the data has been given.

For the qualitative component, participants who agree to be contacted about the qualitative study (by returning a contact details form alongside their quantitative questionnaire) and are selected to be invited will be sent the qualitative participant information sheet and consent form. The site staff will be sent the information sheet and consent form by email.

The researcher will answer any questions, and, if the participant or site staff member is willing to take part, will arrange the time, date and location for the interview. At the start of the interviews, the researcher will go through the participant information sheet or the site staff information sheet with the participant or site staff member, answer any questions and make sure they understand what is involved. They will then obtain informed consent from the participant, taking one signed copy of the consent form, and leaving the participant with a copy for their records.

For interviews that take place remotely via Skype or telephone, the researcher will contact the participant a week ahead of the interview by telephone to go through the information sheet and consent forms with the interviewee, and answer questions. The interviewee will then be asked to post the signed consent form to the interviewer, to reach the interviewer prior to the interview commencing. At the start of the interview, the interviewer will check whether there are any further questions, and make sure the interviewee is still happy to take part in the interview.

3 REGISTRATION & RANDOMISATION

This study is cluster randomised, with each ICON8 site being a separate cluster.

3.1 RANDOMISATION PRACTICALITIES

Randomisation of each site will be performed centrally by the MRC CTU at UCL, after the relevant approvals have been obtained (see **Section 7.1** for more information about the timelines of randomisation). The factorial design of the study means some sites will be allocated to a combination of interventions.

Sites will receive an email from the MRC CTU at UCL explaining what they have been randomised to, and giving detailed instructions for what they need to do.

For sites in the medium and high-recruiting strata, where data will not be collected from all participants, the participants from whom we will attempt to collect data will be pre-specified by the trial statistician, having been chosen at random from the list of all eligible patients at that site. See **Section 5.1.1** for details on how we will replace participants who choose not to respond in medium and high-recruiting strata. Sites in the low-recruiting stratum will be asked to collect data from all eligible participants.

Further details on the process of randomisation can be found in **Section 7.1.**

4 INTERVENTIONS

4.1 INTRODUCTION

Show RESPECT has a cluster randomised factorial design, so sites will be assigned to a combination of the interventions outlined below.

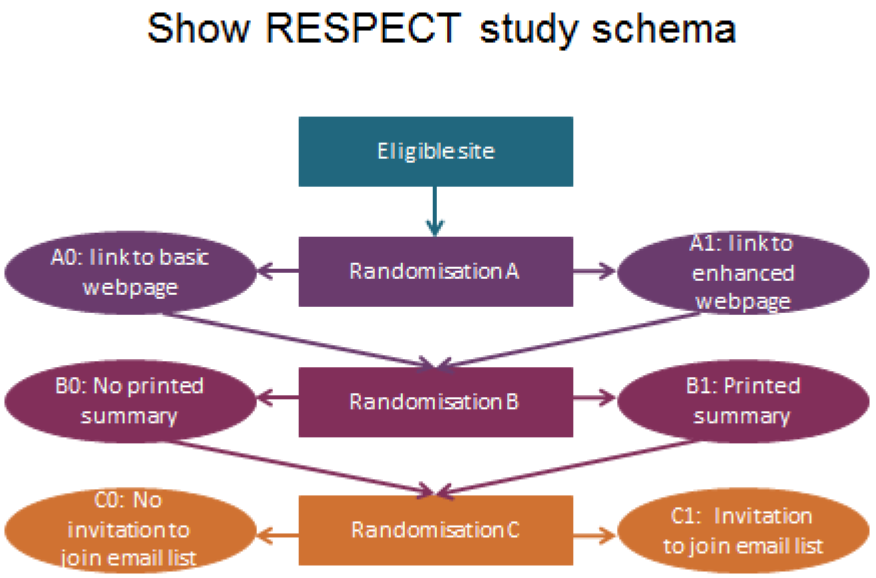

All participants will be sent an Patient Update Information Sheet (based on the HRA guidance and End of Study Information Sheet template https://www.hra.nhs.uk/documents/322/hra-guidance-end-study-pis-v4-1_20-august-2015.pdf) outlining how the results will be communicated to them (according to how their site was randomised), how they can opt out of receiving the results, and issues around future follow-up.

As there is clear evidence that trial participants want to be offered trial results, all participants will have the opportunity to receive the results in some form. The minimum offered will be a link to a basic webpage containing the trial results. This basic webpage will follow the content and structure recommended in the template in the EU Guidelines on Summaries of Clinical Trial Results for Laypersons https://ec.europa.eu/health/sites/health/files/files/eudralex/vol-10/2017_01_26_summaries_of_ct_results_for_laypersons.pdf

Table 2 summarises the main characteristics of the interventions.

Table 2: Summary of intervention characteristics

	BASIC WEBPAGE	ENHANCED WEBPAGE	PRINTED SUMMARY	EMAIL LIST
Opt-in / opt-out	Opt-in	Opt-in	Opt-out	Opt-in
Control of timing of receipt of information	Participant controls timing	Participant controls timing	Site controls timing	CTU controls timing
Access	Internet (via URL given to participant)	Internet (via URL given to participant)	Post	Email
Text content (NB all text will be written following Plain English principles)	EU Lay Summary structure (see Section 4.2)	Participants results summary structure (see Section 4.3)<br>FAQ section that develops as people send in questions	Participants results summary structure (See section 4.3)	Participants results summary structure (see Section 4.3)<br>FAQs as people send in questions
Other content	Link to paper when available.<br>Link to trial register entry.	Links to further information (e.g. lay explanations of terms, the results paper when available, entry of trial register) and support groups.<br>Graphic to illustrate interventions<br>Graphic to illustrate prevalence of common side-effects<br>Simple video of summary of results (see Section 4.3)	Graphic to illustrate interventions<br>Link to paper when available.<br>Link to trial register entry.	Links to further information (e.g. lay explanations of terms, the results paper when available, entry of trial register) and support groups.<br>Graphic to illustrate interventions
Format	Webpage	Webpage	Professionally printed 4-page colour A4 document on 150gsm paper	Email (available in plain text and HTML versions, depending on email client of user)
Design	Clear, bold headings.<br>Body text will be Arial 12pts, black against a white background.	Clear, bold headings.<br>Body text will be Arial 12pts, black against a white background.	Formatted according to MRC CTU at UCL Participant Summary template.<br>Use of clear, bold headings.<br>Body text will be Arial 12pts, black against a white background.	Use a suitable MailChimp template<br>Use of clear, bold headings.<br>Body text will be Arial 12pts, black against a white background.
Static / evolving	Static	Evolving	Static	Evolving

4.2 LINK TO BASIC WEBPAGE

Participants randomised to receive a link to a basic webpage will be given the URL of a webpage in their Patient Update Information Sheet. It will be up to participants as to whether they access it. Each site randomised to this will be given a different URL (pointing to the same page), to allow us to monitor uptake by site, as well as overall number of hits.

The information on the page will follow the structure and 'friendly' versions of the headings for lay summaries mandated by the European Parliament Regulation (EU) No 536/2014 Article 37 (4):

1. Study name
2. Who sponsored this study?
3. General information about the study
4. What patients were included in this study?
5. Which medicines were studied?
6. What were the side effects?
7. What were the overall results of the study?
8. How has this study helped patients and researchers?
9. Are there plans for further studies?
10. Where can I find further information about this study?

https://ec.europa.eu/health/sites/health/files/files/eudralex/vol-10/2017_01_26_summaries_of_ct_results_for_laypersons.pdf

The content of the webpage will be written following the principles of Plain English, and will be reviewed by patient representative(s).

The web page will be laid out with clear headings, and the body text will be Arial 12pts, black against a white background. Section 10 of the webpage will link to the entry on the clinical trial register, and the peer-reviewed paper (when available).

4.3 LINK TO ENHANCED WEBPAGE

Participants randomised to receive a link to an enhanced webpage will be given the URL of a webpage in their Patient Update Information Sheet. Each site randomised to this will be given a different URL (pointing to the same page), to allow us to monitor uptake by site. The enhanced webpage will not be linked from other pages on the website, will not be findable via the site navigation, and search engines will be discouraged from indexing it, so people will need to know the URL to access it, to reduce crossover. It will be up to participants who have been told the URL as to whether they access it.

The information on the webpage will follow the structure and language of the 'Participants Results Summary template' (see Annex 1), adapted for the ICON8 trial. This template has been adapted from the MRCT Center Guidance and Toolkit[19] and the CISCRP template, based on a focus group with cancer trial participants.

1. Thank you
2. What was the study about?
3. Why was the study needed?
4. Who took part in the study?
5. How was the study carried out?
6. What did the study find?
7. How sure can we be about these results?

8. What do these results mean?
    a. What do these results mean for you?
    b. What do these results mean for other people?
9. What difference will these results make?
10. Thank you
11. Further information

The enhanced webpage will use tables and/or graphics to illustrate key points. It will use in-text links to websites aimed at patients with explanations of key terms or concepts (e.g. Cancer Research UK, Target Ovarian Cancer, Ovarian Cancer Action). It will also provide links in the further information section to the support that is available for patients and their loved ones via these organisations, as well as to the trial register entry, the peer-reviewed journal article, and any additional sources of information about the trial and its results (e.g. accurate news stories on trusted websites). The webpage will include a short, simple video of a trial clinician explaining the main results of the trial in lay language. This video will just feature one clinician talking to the camera, covering:

- Introducing themselves and their role in the trial
- Thanking participants for taking part
- Explaining what the trial was testing and how
- Summarising the main results
- Saying what this means for future patients, and why the research results are important

The webpage will also contain a Frequently Asked Questions section, which will invite participants to send any additional questions they have by email or post to the trial team. Questions can be submitted anonymously via an online form using the Opinio survey system. Answers to the questions received will be posted on the website within 3 weeks of receiving them.

The content of the webpage will be written following the principles of Plain English. The web page will be laid out with clear headings, and the body text will be Arial 12pts, black against a white background. Both the content and layout of the enhanced webpage will be reviewed by patient representatives, with the content also being reviewed by a nurse who specialises in providing information about ovarian cancer to patients.

4.4 PRINTED SUMMARY

Participants at sites randomised to use the printed summary will be posted the printed summary by their current trial site, if their current address is known, around three weeks after they have been sent the Patient Update Information Sheet, unless they have opted out.

The printed summary will follow the structure and language of the 'Participants Results Summary template' (see Annex 1), adapted for the ICON8 trial. This template has been adapted from the MRCT Center Guidance and Toolkit[19] and the CISCRP template, based on a focus group with cancer trial participants.

1. Thank you
2. What was the study about?
3. Why was the study needed?
4. Who took part in the study?
5. How was the study carried out?
6. What did the study find?
7. How sure can we be about these results?
8. What do these results mean?

   a. What do these results mean for you?
   b. What do these results mean for other people?
9. What difference will these results make?
10. Thank you
11. Further information

The content of the printed summary will be written following the principles of Plain English. The text of sections 2-10 will be identical to that of the enhanced webpage. The further information section will contain information on relevant patient helplines, how to access the peer reviewed paper and the link to the trial registry entry.

The printed summary will be laid out with clear headings, plenty of white space, and the body text will be Arial 12pts, black against a white background. It may use graphics to illustrate key points. It will be two to four pages long, once formatted. It will be professionally printed on 150gsm paper. Both the content and layout of the printed summary will be reviewed by patient representatives and a nurse who specialises in providing information about ovarian cancer to patients.

4.5 INVITATION TO JOIN EMAIL LIST

Participants randomised to be invited to join an email list will be given a URL to sign-up to the email list in their Patient Update Information Sheet. The URL will take them to a form where participants can enter their email address onto a secure MailMan database, which will not be linked to their trial data. We will use MailChimp to design the email newsletter in a way that works well in different email platforms, including mobile phones.

When participants sign up to the email list, they will receive an email, confirming their subscription and telling them how they can unsubscribe at any time.

The first email with a summary of results (using the same written content as the enhanced webpage, see 4.3, above) will be sent 1 month after sites have received the Patient Update Information Sheets, to allow them time to distribute them to participants, and for participants to sign up. Questions about the results can be submitted anonymously via an online form using the Opinio survey system. An update email will be sent out a month later with answers to any frequently asked questions that have been received since the previous email, and any updates (e.g. links to publications or presentations). Participants who sign up to the email list after the first email has been sent will be sent a welcome email with a link to online copies of any email(s) that have previously been sent.

The content of the emails will be written following the principles of Plain English, and will be reviewed by patient representative(s). The email will be laid out with clear headings, plenty of white space, and the body text will be Arial 12pts, black against a white background. It will use a suitable MailChimp template.

The further information section will contain links to the peer-reviewed journal article and the entry in the trial registry.

4.6 COMPLIANCE & ADHERENCE

We will keep a clear record of what is sent out to each site, to ensure they receive the right information for their randomisation.

Sites will be asked to complete a log of which patients have been sent the Patient Update Information Sheet, which have opted out of receiving printed summaries and which have been sent the printed summary (if applicable to the site's randomisation). We will also keep a log of when participants selected for the quantitative study are sent the questionnaire.

We will record the number of visits to each custom URL, the number of people who sign up to the email list.

5 QUANTITATIVE AND QUALITATIVE DATA COLLECTION

Show RESPECT will collect data from various sources:

- Quantitative data, including primary outcome data, from ICON8 trial participants
- Additional quantitative data from ICON8 site staff and MRC CTU staff
- Qualitative data from ICON8 trial participants and from ICON8 site staff

Predominantly, the quantitative data collection from participants, sites and CTU will be outcome data (see **Section 7**, below), but we will also collect some additional covariate data not already collected as part of the ICON8 trial. All quantitative Show RESPECT data will be collected in a study-specific database, designed according to MRC CTU Standard Operating Procedures for database development. All qualitative Show RESPECT data will be stored in the UCL Data Safe Haven.

A summary of assessments and their timings is given in table 3.

Table 3: Summary of assessments and timings

Data collection type	Method	Timing
Quantitative		
Quantitative data from ICON8 trial participants	Single questionnaire, distributed by site staff, with site-level incentives to increase response rates	To begin at each site 1 month after administration of last intervention (Patient Update Information Sheet sent/ email sent / printed summary sent, depending on randomisation of site) to the last patient at that site. Sites will be reminded of when this is<br><br>Aim to complete data collection within 6 months
Quantitative data from ICON8 site staff	Case Report Forms for site staff to complete; one immediately after intervention delivery, one later (more than one set per site allowed, if several people were involved in the process of communicating results)	Data about the process of communicating results will be collected immediately after interventions have been delivered. Data about the response from patients will be collected 2-3 months after administration of last intervention. Aim to complete data collection within 6 months
Quantitative data from MRC CTU trial staff	Case Report Forms for CTU staff to complete; one immediately after intervention delivery, one later (one set per team member involved in dissemination of trial results)	Data about the process of communicating results will be collected immediately after interventions have been delivered. Data about the response from patients and sites will be collected 2-3 months after administration of last intervention.
Qualitative		
Qualitative data from ICON8 trial participants	Interviews with trial participants, conducted by Annabelle South	To begin approximately two months after site has delivered intervention/Patient Update Information Sheet

Qualitative data from ICON8 site staff	Interviews with site staff, conducted by Annabelle South	To begin two months after site has delivered last intervention

5.1 QUANTITATIVE DATA COLLECTION

5.1.1 ICON8 Participants

We will attempt to collect data from a subset of all ICON8 participants, with per-site numbers varying depending on the total number of participants randomised at each site. See **Section 7**, below, for more details on site strata.

All quantitative participant-level data collection specific to Show RESPECT will be collected on a single questionnaire. This will also have an embedded informed consent element, i.e. the first pages of the questionnaire explains why we would like to collect the data, how data will be handled, and give participants the chance to opt in to providing data.

The questionnaire will be sent by site staff to participants by post, together with a stamped addressed envelope for participants to return their completed forms to the MRC CTU at UCL. Sites will be asked to distribute the data collection materials to participants a month after the last intervention has been delivered at their site. Sites will be sent the questionnaires at this time, pre-populated with the trial ID numbers of relevant participants (i.e. those who we are asking to provide data at their site). Prior to sending these questionnaires out to the selected participants, sites will be asked to confirm that the participant is still alive. This will be done through site staff contacting each selected participant's GP to check the patient has not died. The questionnaire should be sent within a week of this check. This check may only be omitted if the site has had contact with the participant within the preceding two weeks (eg. for a clinic visit), and has no reason to believe the participant is too unwell to receive the questionnaire. In cases of doubt, the GP should be contacted to check the patient is alive.

Sites will be asked to follow-up with participants who have not returned their forms within a month of the forms being sent, to encourage them to return them. Before contacting patients, sites will be asked to confirm the participant is still alive through checking with the participant's GP, if the site has not been in contact with the participant within the preceding two weeks. To boost data collection rates, an incentive (£15) will be offered to sites for each completed questionnaire returned to the MRC CTU. If a pre-selected participant chooses not to complete the questionnaire, or is ineligible for the study, the site will be asked to contact an additional participant as a substitute, if there are other ICON8 participants at the site who were not selected in the initial Show RESPECT sample. A list of up to 6 (for medium recruiting sites) or 12 (for high recruiting sites) substitutes, selected at random (or, where it equals all the eligible participants at a site, ordered at random) will be prepared in advance by the trial statistician, but will not be revealed to sites until required (i.e. participant has confirmed to site that they do not wish to complete the questionnaire, or completed questionnaire has not been returned to the MRC CTU at UCL despite the site having reminded the participant about it three times). Substitute participant numbers will be revealed to sites one at a time, to prevent selection bias.

We will provide sites with a specific log to record each stage of this process for each participant, i.e. the date the Patient Update Information Sheet was distributed, the date postal interventions were distributed (if applicable to their site), the date the patient's GP was contacted to confirm the patient has not died, the date the CRFs were distributed and dates of any further attempts to

contact participants to encourage return of CRFs. These data will be used for central monitoring processes and also to inform various secondary outcome measures related to the process of getting interventions to participants.

Some data from the ICON8 trial will be used in Show RESPECT (year of birth and ICON8 trial arm), to avoid having to complete it again. The ICON8 trial ID number will be used to link these data with the Show RESPECT questionnaire data.

5.1.2 ICON8 SITE STAFF

Site staff will be asked to complete two short Show RESPECT CRFs to collect data on resource use and other secondary outcomes. Data about the process of communicating results (first CRF) will be collected immediately after interventions have been delivered. Data about the response from patients (second CRF) will be collected 2-3 months after administration of last intervention. A CRF set can be collected for each member of staff involved at each site. At a minimum, we will aim to collect one CRF set from each site, from the main person involved in disseminating trial results.

5.1.3 MRC CTU TRIAL TEAM STAFF

MRC CTU trial team staff will be asked to complete two short Show RESPECT CRFs. Data about the process of communicating results (first CRF) will be collected immediately after interventions have been delivered. Data about the response from patients and sites (second CRF) will be collected 2-3 months after administration of last intervention. One CRF set can be completed for each member of staff involved in dissemination of results.

5.2 QUALITATIVE DATA COLLECTION

5.2.1 QUALITATIVE DATA COLLECTION FROM PARTICIPANTS

We will **carry out semi-structured interviews with participants to explore**

1. What are the experiences and views of women in the ICON8 ovarian cancer trial on how the results were communicated to them?
2. What aspects of the mode of communication influence satisfaction with how the results are communicated, and why?
3. What other factors influence how satisfied women taking part in the ICON8 trial are with how the results are communicated to them?

5.2.1.A Setting

We will invite participants from at least 8 English hospitals that are ICON8 sites to take part in the qualitative study. Participants will be offered the choice of being interviewed in their own homes, or, if that is not possible, via Skype video call. If a Skype video call is not possible, a telephone interview will be carried out instead.

5.2.1.B Who will be recruited and how?

We will use purposive sampling to select participants that give us a range of:

- interventions they were offered (this will be done by selecting 8 sites that were randomised to the 8 different combinations of modes of communication)
- reported satisfaction with how the results were communicated (identified from the quantitative survey carried out as part of the Show RESPECT study)
- level of education (identified from the quantitative survey carried out as part of the Show RESPECT study)

- internet usage (identified from the quantitative survey carried out as part of the Show RESPECT study)

Initially we will pick 8 ICON8 sites, each allocated to a different combination of interventions) from which to select participants. These sites will be selected to as being feasible for the interviewer to travel to, given the resource constraints of this study. We will attempt to recruit at least two women from each site. If it is not possible to recruit two women at a particular site, we will add another site allocated to that combination of interventions. Our minimum sample size will be 16 (a minimum of 2 per combination of interventions they were offered, meaning we interview at least 8 women offered each intervention), but we will continue to recruit until we reach theoretical saturation. While recruiting we will keep a record of the characteristics of volunteers with regard to the criteria we are interested in (including the factors listed above, and ICON8 arm and age), to ensure we are reaching the minimum number for each criteria. As the analysis progresses, we will consider whether to use a theoretical sampling approach to test emerging theories.

During the quantitative data collection, participants will be asked to complete a contact details form if they are willing to be contacted about the qualitative study. Those that return the contact details form, and who are chosen to be part of the qualitative sample, will be sent the patient information sheet and consent form by the qualitative researcher (see **Section 2.6** for the consent process). Participants will be offered a voucher worth £20 to recompense them for their time. We will exclude participants who do not have sufficient verbal English skills to allow them to participate in the interviews. Informed consent will then be taken face-to-face at the beginning of the qualitative interview.

5.2.1.C Data collection

We will carry out semi-structured interviews with participants, using a topic guide that will be amended as needed as the data collection and analysis progresses. The interviews will be audio recorded and subsequently transcribed verbatim. The transcriptions will be checked back against the recordings for accuracy, any identifying data (e.g. names) will be removed. The researcher will also keep field notes after each interview, and a reflexive journal. The interviews will be carried out by Annabelle South.

5.2.2 Qualitative Data Collection from Site Staff

We will carry out semi-structured interviews with site staff to explore:

1. What are the experiences and views of site staff in communicating the results of the ICON8 trial to the trial participants using the approaches tested in the Show RESPECT study, and how are these views shaped by their clinical setting or the interventions their site was randomised to?
2. Which approaches to communicating the results of the ICON8 trial to participants are acceptable and feasible to implement for site staff?

5.2.2.A Setting

We will invite site staff from a minimum of 12 of the 79 English hospitals that are ICON8 sites to take part in the qualitative study. Site staff who are willing to be interviewed will be given the choice between being interviewed at their hospital, or via a Skype video call if that is not possible. If a Skype video call is not possible, a telephone interview will be carried out instead.

5.2.2.B Who will be recruited and how?

We will use purposive sampling to identify site staff to invite to be interviewed. We aim to interview 6 site staff who were involved in communicating the results to participants, and were based at a site randomised to send out printed summaries to participants, and 6 from sites that were not

randomised to send out printed summaries (a minimum sample size of 12). This will be split between high, medium and low-recruiting sites, as there may be substantial differences in the work involved in this, depending on the number of participants at the site. Further interviews will be carried out until theoretical saturation is reached. We will start by inviting site staff involved in communicating results at the sites we select for the participant interviews, in case we can learn anything from comparing the responses of participants and their research nurses. We will then invite nurses from additional sites that meet the criteria we still need, and are within feasible travel distance for the lead researcher. The lead researcher will contact nurses via email with information about the study, the site staff information sheet and consent form.

5.2.2.C Data collection

We will carry out semi-structured interviews with site staff, using a topic guide that will be amended as needed as the data collection and analysis progresses. The interviews will be audio recorded and subsequently transcribed verbatim. The transcriptions will be checked back against the recordings for accuracy, any identifying data (eg. names) will be removed. The researcher will also keep field notes after each interview, and a reflexive journal. The interviews will be carried out by Annabelle South.

5.2.3 ANALYSIS OF QUALITATIVE DATA

Analysis will be carried out alongside data collection, to allow for ongoing revision of the topic guide to further investigate emerging themes and theories. The first step will be familiarisation with the data, by listening to the recordings and reading the transcripts a number of times, recording ideas for initial codes. A thematic analysis approach will be employed. Both inductive and deductive approaches will be used to code the data, using organisational, substantive and theoretical codes. Codes will be categorised into themes and sub-themes. As a theory emerges, I will search for deviant cases, and, if required, conduct further interviews using theoretical sampling to test the emerging theory.

5.3 PARTICIPANT MANAGEMENT

We will ask sites to attempt to contact each ICON8 participant up to three times about the Show RESPECT study. If a participant cannot be contacted after three attempts, we will assume them to be uncontactable for study purposes. We will ask sites to notify us of these cases via the site-specific log mentioned in **Section 5.1.1**, above; this section also explains how we will handle cases of participants who do not wish to provide data for Show RESPECT.

It is conceivable that an ICON8 participant may transfer to a different hospital between the end of the trial and the dissemination of results, or even between dissemination of results and data collection. Such cases will be handled on a case-by-case basis, but the default would be for the new site to disseminate results and collect study data.

6 QUALITY ASSURANCE & CONTROL

6.1 RISK ASSESSMENT

The Quality Assurance (QA) and Quality Control (QC) considerations have been based on a formal Risk Assessment, which acknowledges the risks associated with the conduct of the study and how to address them with QA and QC processes. QA includes all the planned and systematic actions established to ensure the study is performed and data generated, documented and/or recorded and reported in compliance with the principles of GCP and applicable regulatory requirements. QC includes the operational techniques and activities done within the QA system to verify that the requirements for quality of the study-related activities are fulfilled.

The Risk Assessment has been reviewed by the CTU's Research Governance Committee (RGC) and has led to the development of the study data management and monitoring processes. In accordance with MRC CTU Standard Operating Procedures, these processes have been reviewed by the internal Quality Management Advisory Group. The monitoring processes are shown in **Section 6.2**, below; a separate data management plan has been developed alongside this protocol.

6.2 MONITORING PROCESSES

There will be no visits to ICON8 sites as part of Show RESPECT; all monitoring activity will be conducted centrally at the MRC CTU.

Monitoring and oversight of Show RESPECT will be provided by the Study Management Group (SMG). This Group will conduct regular reviews of the following, taking any corrective actions if required:

- Numbers of Patient Update Information Sheets distributed to ICON8 participants, overall and per site;
- Uptake of each intervention, e.g. numbers signing up to electronic mailing list, numbers accessing ICON8 results webpages;
- Return rates for patient, site and CTU quantitative data collection;
- Review of data collection and refusal rates against the planned sample size;
- Any other arising issues.

The SMG will review returned participant, site and CTU forms and questionnaires in a standardised fashion to check that data are valid. The Show RESPECT database will also identify data validity issues in an automated fashion. Where possible, issues will be resolved, although scope for resolution for the patient reported outcomes is relatively limited.

6.3 ADVERSE EVENTS

Although there is limited scope for Show RESPECT data collection to unearth or cause issues of participant wellbeing, the SMG will also respond appropriately to any issues that arise.

Returned participant CRFs will be systematically checked on arrival to look for any issues of concern (e.g. to do with participants finding ICON8 results extremely upsetting, or issues of potential harm where we might have a duty of care to act such as participant reports of suicidal thoughts). Any issues found will be passed in the first instance to the Clinical Reviewer and discussed, involving the ICON8 team if appropriate. If it is considered necessary by the Clinical Reviewer, we will inform the site staff of what we have found so that they can take appropriate action.

We will keep a record of both the checks of each participant CRF (i.e. to confirm it was checked and the presence or absence of any issues to escalate) and of any discussion and escalation of any issues we find. We will also make clear that we may do this in the pre-data collection information for participants.

If a participant becomes upset during a qualitative interview, the researcher will offer the participant the option to take a break, skip any question that they find distressing, or end the interview. At the end of the interview participants will be referred to sources of further support (local support groups and the Target Ovarian Cancer and Ovacome helplines). If something occurs during the interview that the researcher feels requires urgent attention (eg. if the participant reveals suicidal thoughts), the researcher will explain that they will refer the participant to their GP, site staff or emergency mental health team for additional support, as relevant.

7 STATISTICAL CONSIDERATIONS

7.1 METHOD OF RANDOMISATION

The participating sites will be split initially into three strata:

1. Low volume sites: sites that have 5 or fewer alive participants
2. Medium volume sites: sites that have 6-12 alive participants
3. High volume sites: sites that have 13 or more alive participants

The details of how randomisation is conducted depend on how quickly sites are able to obtain the required approvals so as to participate, and hence can be randomised.

Firstly we plan for the scenario in which all sites obtain approvals in good time, in which case randomisation would be conducted at one time point after all approvals are obtained. Because there are 8 study arms in this trial these strata will be 'capped' at a multiple of 8, and 'surplus' sites selected at random from within each stratum would form a fourth 'surplus' stratum. For example if 42 low volume, 18 medium volume and 17 high volume sites are recruited then two low volume sites, two medium volume sites and one high volume site would be selected at random and together they would form a fourth stratum for randomisation. This would leave the other strata of sizes a multiple of 8: 40 low volume, 16 medium, and 16 high volume sites. Randomisation would be performed by computerised random permutation in Stata software within each stratum separately.

Secondly we plan for the scenario in which approvals are delayed for some sites, but permitting randomisation of sites ready to participate earlier to avoid undue delay. In this case we shall generate a randomisation sequence within each volume stratum based on randomly permuted blocks of size 8 (no surplus stratum is used in this scenario). Once many sites have approvals in place, and the rate of further approvals being obtained has slowed, then we would randomise all sites with approvals in place. We would apply the randomisation sequence to sites after randomly permuting them within each stratum in Stata software. We anticipate conducting this initial randomisation for all three strata at the same time. However in the event that approvals accumulate quickly in some strata but not in others, and in particular if in one or more strata fewer than 8 sites are ready to be randomised, we may perform the initial randomisation for some strata earlier than for others. After the initial randomisation for each stratum, as further sites obtain approvals we shall allocate them in real time to the next allocation in the sequence.

7.2 OUTCOME MEASURES

7.2.1 PRIMARY OUTCOME MEASURE

The primary outcome measure (collected from participants) will be:
How satisfied are you with the way you found out the results of ICON8 (rather than the results themselves)? This will be measured using a Likert scale (1=Very unsatisfied, 2=Somewhat unsatisfied; 3=neither satisfied nor unsatisfied; 4=Somewhat satisfied; 5=Very satisfied).

7.2.2 SECONDARY EFFECTIVENESS OUTCOMES (COLLECTED FROM PARTICIPANTS)

- The information about the trial results told me everything I wanted to know (1=Strongly disagree, 2=slightly disagree, 3=neither agree nor disagree; 4=slightly agree; 5=strongly agree)

- Ease of understanding the results (1=very hard; 2=quite hard; 3=neither hard nor easy; 4=quite easy; 5=very easy)
- How upsetting did you find the results? (1=not at all; 2=slightly; 3=moderately; 4=very)
- How willing do you think you would be to take part in research again in the future? 1=Very unwilling, 2=quite unwilling, 3=not sure; 4=quite willing; 5=very willing)
- How likely are you to recommend taking part in research to friends or family? (1=Very unlikely, 2=quite unlikely, 3=not sure; 4=quite likely; 5=very likely)
- Are you glad you found out the results?
- Do you regret finding out the results?

7.2.3 Secondary process outcomes (collected from participants)

- Reported uptake of the intervention(s) offered
- Proportion of participants who wanted to find out the results who reported finding out the results
- Proportion of participants who did not want to find out the results who reported finding out
- Ease of finding out the results (1=very hard; 2=quite hard; 3=neither hard nor easy; 4=quite easy; 5=very easy)
- Would they prefer to have been given the opportunity to find out the results in a different way? If so, how and why?
- If they found out the results in several ways, which did they prefer and why?

Secondary analyses will assess whether these results varied by the following subgroups:

- By reported education level
- By reported internet use
- By ICON8 trial arm
- By strata - process outcomes only
- By English as first language vs not

7.2.4 Secondary process outcomes (collected from site staff):

- Any concerns with the interventions or process
- Time taken for each of the interventions (and by whom)
- Who delivered the interventions
- Challenges faced implementing the interventions
- Number of queries received following results being communicated
- Costs incurred by the site in implementing the interventions
- What would they like to do differently for the next trial they are involved in communicating results for?
- What proportion of patients did the Patient Update Information Sheet go out to? (Collected via log)
- For sites in the Printed Summary arm, what proportion of patients did the printed summary get posted to? (Collected via log)
- For sites in the Printed Summary arm, how participants many opted out? (Collected via log)

7.2.5 Other secondary outcomes collected from Site Staff

- Site staff's preferred method of communicating the results to participants
- Do they think any of the interventions they were allocated to should become standard practice for trials they are involved in?

- Would they prefer to have given participants a different way to find out the results? If so, what and why?

7.2.6 SECONDARY PROCESS OUTCOMES (COLLECTED FROM CTU STAFF/DATA):

- Any concerns with the process
- Time taken at CTU for each of the interventions
- Challenges faced implementing the interventions
- Number of queries received from participants or sites about the interventions or results
- Costs incurred by the CTU in implementing the interventions
- What would they like to do differently for the next trial they are involved in communicating results for?
- Uptake of basic and enhanced webpages, by site (from analytics data)
- Sign-ups to email list

7.2.7 OTHER SECONDARY OUTCOMES COLLECTED FROM CTU STAFF

- CTU staff's preferred method of communicating the results to participants
- Concerns with the interventions
- Would they prefer to have given participants a different way to find out the results? If so, what and why?

7.3 SAMPLE SIZE

Thus far 37 sites have agreed in principle to participate: 13 low volume, 12 medium and 12 high. Low volume sites (see **Section 7.1** for definition) will be asked to collect outcome data from all alive participants. Medium volume sites will be asked to collect outcome data from 6 participants, and high volume sites will be asked to collect outcome data from 12 participants, whose trial numbers will be selected at random by the MRC CTU at UCL.

We anticipate that therefore at least 255 potential participants will be initially invited to provide outcome data. Where there are refusals at medium and high volume sites then randomly selected 'replacement' participants will be subsequently invited to provide data so as to attempt to reach the site specific data collection targets. We anticipate collecting data from at least 222 participants, an average of 6 per site, and anticipate the coefficient of variation of the number of outcomes collected per site to be around 0.6. Little information concerning the likely intracluster correlation coefficient (ICC) for the primary outcome is available so we consider values between 0.01 (low) and 0.05 (moderate).

The primary outcome is ordinal but for simplicity, because of lack of knowledge of its likely distribution and to be scientifically conservative, we consider it as a binary outcome for our power calculations. We consider that even if outcomes are generally positive that no more than 80% of respondents will report being very satisfied and similarly that even if outcomes are negative that no more than 80% will indicate being very dissatisfied. As such we expect sufficient variation that we can examine improvement in satisfaction where the more satisfied group has prevalence between 20 and 80%. We consider power to detect an effect for any of the three interventions, for simplicity considering each in turn i.e. effectively conducting a power calculation for each intervention assuming the other two have no effect. We acknowledge that should multiple interventions have effects then the power will vary somewhat from that presented here.

At a low value of the ICC our sample size (considering 18 sites with and without an intervention) provides 80% power to detect an increase from 20 to 38% in the more satisfied group, or from 50 to

70% or from 80 to 93%. Should the ICC be moderate at 0.05 then the sample size provides 80% power to detect an increase from 20 to 40% or 50 to 72% or 80 to 95%.

7.4 INTERIM MONITORING & ANALYSES

As the trial interventions will all take place within a narrow timeframe, there will be no interim monitoring or analysis.

7.5 ANALYSIS PLAN (BRIEF)

The analyses will be described in detail in a full Statistical Analysis Plan agreed before final analysis begins. This section summarises the main issues.

Our primary analysis of the primary outcome will be based on the intention to treat principle. The effect of all three interventions will be estimated simultaneously by fitting an ordinal logistic regression model with random effects for site and fixed effects for each intervention. This will lead to an estimated odds ratio and 95% confidence interval for each intervention effect. We make no allowance for multiple testing of three interventions as we consider each to be distinct and its analysis to answer a distinct question. We do not anticipate interaction between the three interventions, but shall test the three pairwise interactions and shall report the effect of all the seven combination intervention arms relative to the control arm.

In secondary analysis we will exclude sites or participants that did not engage with the allocated intervention.

In planned subgroup analysis we shall test interaction effects to assess whether the effect of each intervention differs by arm of the treatment trial, by age, by whether the participant reports their first language to be English, by education level, and by reported internet use.

8 REGULATORY & ETHICAL ISSUES

8.1 COMPLIANCE

8.1.1 REGULATORY COMPLIANCE

Show RESPECT complies with the principles of the Declaration of Helsinki. It will also be conducted in compliance with the approved protocol, the principles of Good Clinical Practice (GCP) as laid down by the European Commission Directive 2005/28/EC with the implementation in national legislation in the UK by Statutory Instrument 2004/1031 (The Medicines for Human Use [Clinical Trials] Regulations 2004) and subsequent amendments, the UK Data Protection Act (DPA number: Z6364106), and the UK Policy Framework for Health and Social Care Research.

8.1.2 DATA COLLECTION & RETENTION

CRFs and administrative documentation will be kept in a secure location (for example, locked filing cabinets in a room with restricted access) and held for a minimum of 5 years after the end of the study, in line with current University College London retention schedules.

Although Show RESPECT is not a clinical trial, for regulatory purposes the source data will be the original questionnaire data provided by ICON8 participants, site staff and CTU staff.

8.2 ETHICAL CONDUCT

8.2.1 ETHICAL CONSIDERATIONS

This study addresses an ethical issue itself, namely that clinical trial participants want to hear results of trials they participate in, but often do not. We hope to generate evidence to help researchers conduct their trials more ethically in this respect.

The following additional ethical considerations should be noted:

- There is a risk that ICON8 trial participants are not satisfied with the dissemination method they are allocated to, or that it poses significant barriers to their receiving the ICON8 trial results: there is currently limited evidence to support the choice of dissemination method, and we are aiming to measure satisfaction. All ICON8 participants will have the chance to hear about the trial results via a method that is at least theoretically feasible and that has been agreed to be feasible in feedback from pre-trial Patient and Public Involvement activity and from site staff representatives.
- There is a risk that ICON8 trial participants are upset by the results of the trial: wording of all communications will be carefully chosen, and there will be Patient and Public Involvement contributions to all interventions. We will offer links and resources to help people deal with the trial results, and we will refer people to their nurse or clinician to discuss results further.
- Questionnaire completion: the participant questionnaire does not include questions collecting sensitive or personal data, and we will make clear to all participants that completing the questionnaire is entirely voluntary.
- As mentioned in **Section 2.6**, we do not plan to tell ICON8 participants about the details of the Show RESPECT study design, including the fact of the site-level randomisation to different methods of disseminating ICON8 trial results. We believe the risk of the randomisation is extremely minimal, given the absence of clear evidence about best methods for disseminating results. The Show RESPECT study ultimately aims to improve

people's experience of clinical trial participation, and the potential negative effects of information overload in this study – namely a reduced questionnaire return rate and resulting reduction in the study's statistical power – would hinder us from doing this.

8.2.2 Favourable Ethical Opinion

We will not commence the study (i.e. we will not begin sending out Patient Update Information Sheets) until we have received favourable ethical approval from a Health Research Authority Research Ethics Committee, and overall HRA approval for the study. Any further amendments will be submitted and approved by the ethics committee and HRA (as required).

The study has been developed with Patient and Public Involvement (PPI) to ensure that its design is feasible and acceptable to potential participants, and to ensure its outcomes and potential impact are relevant to the population who may benefit from its results. PPI also helps to ensure transparency and accountability throughout this research. PPI activity will continue for the duration of the study, including dissemination of study results.

8.3 OTHER APPROVALS

The protocol will be submitted by those delegated to do so to the relevant R&D department of each participating site or to other local departments for approval as required in each country. A copy of local of R&D approval (or other relevant approval) should be forwarded to the CTU. On receipt of all relevant approvals the site will be randomised and then specific Patient Update Information Sheet will be provided to the site for distribution to patients.

8.4 STUDY CLOSURE

The main phase of the study will end when, for all eligible ICON8 participants (or their replacements), we have completed a questionnaire, or we know we are not going to receive one (e.g. because of lack of response to site requests), or 6 months has passed since the start of data collection.

9 INDEMNITY

No specific indemnity for this study has been sought, however University College London, as sponsor of the study, provides insurance to cover any potential issues arising through study participation. In addition, the ICON8 trial has its own indemnity arrangements which will apply to all participants in Show RESPECT.

10 FINANCE

The Show RESPECT study is being funded by the MRC Clinical Trials Unit at UCL from their core funding from the Medical Research Council for trial conduct methodology research (grant number MC_UU_12023/24).

11 OVERSIGHT & TRIAL COMMITTEES

Details of study committees are given below. There is no Independent Data Monitoring Committee (IDMC) for this study. There is not sufficient justification for any planned interim analyses of safety or efficacy, given the short duration of the study, the improbability of any serious harm occurring to participants on any of the study intervention arms and the fact that data collection does not start until interventions have already been delivered.

11.1 STUDY MANAGEMENT GROUP (SMG)

The Study Management Group (SMG) will consist of the Chief Investigator, two Co-Investigators, and the trial manager, who will lead in the day-to-day running of the study. This will include ensuring that sites are adherent to the protocol, producing any protocol amendments as necessary, dealing with issues at the sites enrolled in the study, and high level review of data collected as part of the study. The SMG will convene as regularly as required during the study, depending on the level of activity (including central monitoring) required at each stage.

11.2 STUDY STEERING GROUP

The Study Steering Group (SSG) for the Show RESPECT trial will convene approximately every six months and when thought necessary by the SMG. The SSG's purpose in the trial will be to act as an advisory committee to help guide the SMG in the running of the trial and provide expert advice. The SSG members have expertise in qualitative research, statistical methodology, bioethics, and linguistics. There will also be at least one Patient and Public Involvement contributor on the SSG. This range of expertise will help inform study conduct and provide guidance with problem-solving when necessary.

The SSG will be chaired by one of the study Chief Investigator/Co-Investigators and there will be no formal voting process. Instead, opinions offered during the SSG will be considered by the SMG and implemented where it is thought to be appropriate.

12 PATIENT AND PUBLIC INVOLVEMENT

Patient and Public Involvement (PPI) in research is defined by INVOLVE (an advisory group established by the NIHR) as research being carried out ‘with’ or ‘by’ members of the public rather than ‘to’, ‘about’ or ‘for’ them. INVOLVE intends ‘public’ to include patients, potential patients, carers and other users of health and social care services, as well as people from organisations that represent people who use services. In some cases, this may include involvement of a trial’s participants in guidance or oversight of a trial.

12.1 POTENTIAL IMPACT OF PPI

This study is about trying to improve the participant experience of receiving (or not receiving) trial results. PPI is essential to ensuring we choose interventions and outcome measures that are relevant and acceptable to participants, and in making sure our tools to collect data are user-friendly. Working with ovarian cancer groups will help us to communicate the results to key stakeholders.

12.2 IDENTIFYING PPI CONTRIBUTORS

See **Sections 12.3** and **12.4**, below, for descriptions of PPI activity before and during the Show RESPECT study. People for the first focus group were identified through the CRUK Involvement Network. Contributors to the survey were identified through the CRUK Involvement Network and the UK CAB. Participants in the second focus group were recruited via CRUK’s Involvement Network, Target Ovarian Cancer, Ovacome, and other ovarian cancer support groups. The Patient representative was identified through her involvement in ICON8.

12.3 PROTOCOL DESIGN AND STUDY SETUP

During the design of this study we have done the following PPI activities:

- Focus Groups and Surveys
  - An initial focus group was carried out in association with Cancer Research UK to discuss possible interventions for use in the trial, based on an initial literature review. This group was also used to inform the development of a template for written summaries of trial results.
  - A further online survey to collect information on patient perspectives around proposed interventions for the trial was completed by 53 respondents in September 2017. This survey further narrowed the list of interventions to be used in the trial, and also provided information on the choice of primary outcome measure.
  - The trial outcome measurements and interventions were finalised after conducting a dedicated patient discussion group in January 2018 to assess the response of patients taking part in ovarian cancer trials. These patients were recruited using patient and cancer networks, and both open and closed questioning techniques used to determine the efficacy and acceptability of the proposed outcome measures to a patient group similar to the sample of interest in the SHOW RESPECT trial.
- Patient Advocate
  - A patient representative was invited to act in an advisory capacity for the SMG. This representative was also a patient representative on the ICON8 TMG, and they were selected to ensure that patient interests were represented when conducting the

trial. Her initial involvement was to review the draft text for the interventions from the patient perspective for acceptability and comprehensibility.

- Patient groups
  - We have discussed this study with Target Ovarian Cancer and Ovacome. Target Ovarian Cancer has provided feedback on the draft text of the interventions, and both organisations have supported recruitment to our PPI activities.

We have budgeted to hold a PPI workshop during the analysis stage of the project, and for a patient representative to be involved in the steering group.

12.4 PPI IN THE ONGOING RUNNING OF STUDY

The patient representative from ICON8 will be asked to attend the SSG meetings during the running of the study, and contribute to the discussions of the group as issues arise. We have budgeted for this activity.

12.5 INTERPRETING AND PLANNING DISSEMINATION OF STUDY RESULTS

When we have the data and initial findings from the study, we will hold another discussion group for women in ovarian cancer trials, to discuss the findings, and get their views on what the key messages should be, and how to prioritise them. This activity has been included in the budget.
We will work with Target Ovarian Cancer, Ovacome and CRUK to communicate the results to patients and those involved in running ovarian cancer trials.

13 PUBLICATION AND DISSEMINATION OF RESULTS

The results of this study will be written up for publication in peer-reviewed journal(s). We will also seek to present the results at a trial methodology or cancer research conference. We will also present our results at a seminar at the MRC CTU at UCL.

In order to avoid disputes regarding authorship, it is important to establish a consensus approach that will provide a framework for all publications derived in full or in part from this clinical trial. The following approach is derived from the Lancet and from the publication policies used in other MRC clinical trials:

- All publications are to be approved by the study steering group before submission for publication. The steering group will resolve problems of authorship and maintain the quality of publications.
- Any publications from this study will comply with the MRC and UCL Open Access policies. All conference presentations will be made available as soon as possible after the event via the MRC CTU at UCL website. All publications will acknowledge the trial's funding sources.
- The SMG will decide on a lead author for each paper, who is responsible for coordinating the writing of the paper, and deciding co-authors.
- The Members of the study steering group will be invited to be on the Writing Committee, alongside scientific and operational members of the ICON8 team who have been involved in the study. For publications where there are no named authors, the paper will be published in the name of the Show RESPECT collaborators, and members of the Writing Committee will be identified. Members of the steering group who choose not to contribute to the Writing Committee will be listed with their affiliations in the acknowledgements section of any publications from the study.
- The study management group will maintain a list of site staff involved in this study to be presented in an appendix at the end of the paper. This list will include people who contributed to the investigation being reported but who are not members of the writing committee.
- All headline authors in any publication arising from the study must have a made a substantive academic or project management contribution to the work that is being presented. “Substantive” must be defined by a written declaration of exactly what the contribution of any individual is believed to have been. In addition to fulfilling the criteria based on contribution, additional features that will be considered in selecting an authorship group will include the conduct of analyses, leadership and coordination of the project in the absence of a clear academic contribution.

To communicate the results to patients we will engage with patient groups, including Target Ovarian Cancer, Ovacome and Cancer Research UK. We will ask them, if they think it is appropriate, to share the results with patients via their communications channels.

We will keep the HRA informed of the progress and results of this study. One of the members of the SSG leads the HRA’s work in this area. The MRCT at Harvard produce guidelines on this topic, so we will also keep them informed of the progress and results of the study, via having a representative from the MRCT on the SSG. We hope these good links will facilitate consideration of the implications of our results for guidelines.

We will develop summaries of the results of this study in formats that will engage non-scientific audiences. This may include infographics, a video, animation and/or podcast. We will take into

account the results of this study when deciding how to communicate the results of this project to participants.

14 DATA SHARING

Data will be shared according to the CTU's controlled access approach, based on the following principles:

- No data should be released that would compromise an ongoing trial or study.
- There must be a strong scientific or other legitimate rationale for the data to be used for the requested purpose.
- Investigators who have invested time and effort into developing a trial or study should have a period of exclusivity in which to pursue their aims with the data, before key trial data are made available to other researchers.
- The resources required to process requests should not be under-estimated, particularly successful requests which lead to preparing data for release. Therefore adequate resources must be available in order to comply in a timely manner or at all, and the scientific aims of the study must justify the use of such resources.
- Data exchange complies with Information Governance and Data Security Policies in all of the relevant countries.

Data specific to Show RESPECT will be available for sharing following publication of the main Show RESPECT results in a peer-reviewed journal. Researchers wishing to access these data should contact the Study Management Group in the first instance.

Some data used in Show RESPECT have been collected primarily for the ICON8 clinical trial. Any researchers also wishing to access these data should contact the ICON8 Trial Management Group.

15 PROTOCOL AMENDMENTS

**Protocol v1.0 11-May-2018**	
**Protocol v2.0 24-Jul-2018**	
**Changes made**	**Sections updated**
Addition of Ethics committee reviewing the study and the Clinical Research Network the study has been adopted by.<br><br>Also the address of the Coordinating site has been corrected	General Information
Additional wording has been added regarding participant status prior to distributing the ICON8 Feedback Questionnaire:<br><br>***Prior to sending these questionnaires out to the selected participants, sites will be asked to confirm that the participant is still alive. This will be done through site staff contacting each selected participant's GP to check the patient has not died. The questionnaire should be sent within a week of this check. This check may only be omitted if the site has had contact with the participant within the preceding two weeks (eg. for a clinic visit), and has no reason to believe the participant is too unwell to receive the questionnaire. In cases of doubt, the GP should be contacted to check the patient is alive.***<br><br>**Additional wording has been added regarding participant status prior to chasing them for the complete ICON8 feedback questionnaire:**<br><br>***Before contacting patients, sites will be asked to confirm the participant is still alive through checking with the participant's GP, if the site has not been in contact with the participant within the preceding two weeks.***	5.1.1 ICON8 Participants
**Protocol v3.0 20-Aug-2018**	
**Change made**	**Sections updated**
Update to the sample size, to take into consideration the number of sites that have agreed in principle to be involved in the study.	Summary of Trial<br>Section 7.3 Sample Size
The Trial Assessment Schedule (table 1), has been updated:<br>- To be ICON8 participant specific and therefore remove the site and MRC CTU data collection timelines.<br>- The timeline for distribution of the results email to participants who have signed up to the email	Trial Assessment Schedule

list was amended to 1 month after the first site is randomised to the intervention.	
Addition of site staff consent process for the qualitative study and the addition of the following text for the method of consent for Skype and telephone interviews:<br><br>***For interviews that take place remotely via Skype or telephone, the researcher will contact the participant a week ahead of the interview by telephone to go through the information sheet and consent forms with the interviewee, and answer questions. The interviewee will then be asked to post the signed consent form to the interviewer, to reach the interviewer prior to the interview commencing. At the start of the interview, the interviewer will check whether there are any further questions, and make sure the interviewee is still happy to take part in the interview.***	Section 2.6 Consent
Addition of text to note that participants can submit questions anonymously via an online form using the Opinio survey system.	Section 4.3 Link to Enhanced Webpage<br>Section 4.5 Invitation to join email list
Amendment of the heading of item 10 in the contents of the summary from “Conclusion” to “Thank you” based on feedback from patient representatives.	Section 4.3 Link to Enhanced Webpage<br>Section 4.4 Printed summary
Amendment of the numbers of sections that will be identical to the enhanced webpage from 1-11 to 2-10.	Section 4.4 Printed summary
Amendment of text in second bullet point from “ICON8 trial staff at the CTU” to “MRC CTU staff”<br><br>Clarification that the database will store all quantitative data, rather than all data from the study.	Section 5 Quantitative and Qualitative Data Collection
Correction of timing of qualitative data collection from ICON8 participants to start two months after interventions (to match table 1).	Table 3 Summary of assessments and timings
Change from “the first page” to “the first pages” when describing where the informed consent information will be in the participant questionnaire.	Section 5.1.1 ICON8 participants
For the Site Staff qualitative interview the option of a telephone call for the interview if a hospital visit of Skype call cannot take place.	Section 5.2.2. A Setting
Amendment to the method of randomisation to note that randomisation will be dependent on when sites obtain all the required approvals. Please see the following wording change:<br><br>***However if additional sites are recruited beyond the target within any stratum then a ‘surplus group’ will be selected at random from the stratum and they will be***	Section 7.1 Method of Randomisation

reassigned to a fourth 'surplus' stratum. For example if 42 low volume, 18 medium volume and 17 high volume sites are recruited then two low volume sites, two medium volume sites and one high volume site will be selected at random and together they will form a fourth stratum for randomisation.<br><br>***Randomisation will be performed by computerised random permutation in Stata software within each stratum separately, allocating at least 9 sites to each of the 8 study arms balanced across strata. This will be completed per site once all required approvals are in place.***<br><br>New wording:<br><br>***The details of how randomisation is conducted depend on how quickly sites are able to obtain the required approvals so as to participate, and hence can be randomised.***<br><br>***Firstly we plan for the scenario in which all sites obtain approvals in good time, in which case randomisation would be conducted at one time point after all approvals are obtained. Because there are 8 study arms in this trial these strata will be 'capped' at a multiple of 8, and 'surplus' sites selected at random from within each stratum would form a fourth 'surplus' stratum. For example if 42 low volume, 18 medium volume and 17 high volume sites are recruited then two low volume sites, two medium volume sites and one high volume site would be selected at random and together they would form a fourth stratum for randomisation. This would leave the other strata of sizes a multiple of 8: 40 low volume, 16 medium, and 16 high volume sites. Randomisation would be performed by computerised random permutation in Stata software within each stratum separately.***<br><br>***Secondly we plan for the scenario in which approvals are delayed for some sites, but permitting randomisation of sites ready to participate earlier to avoid undue delay. In this case we shall generate a randomisation sequence within each volume stratum based on randomly permuted blocks of size 8 (no surplus stratum is used in this scenario). Once many sites have approvals in place, and the rate of further approvals being obtained has slowed, then we would randomise all sites with approvals in place. We would apply the randomisation sequence to sites after randomly permuting them within each stratum in Stata software.***	

We anticipate conducting this initial randomisation for all three strata at the same time. However in the event that approvals accumulate quickly in some strata but not in others, and in particular if in one or more strata fewer than 8 sites are ready to be randomised, we may perform the initial randomisation for some strata earlier than for others. After the initial randomisation for each stratum, as further sites obtain approvals we shall allocate them in real time to the next allocation in the sequence.	
Minimum length of time CRFs and documentation will be stored after the end of the study has been updated from 10 years to 5 years.	Section 8.1.2 Data collection and retention

16 REFERENCES

1. Partridge AH, Wong JS, Knudsen K, Gelman R, Sampson E, Gadd M, Bishop KL, Harris JR, Winer EP: **Offering participants results of a clinical trial: sharing results of a negative study**. *Lancet* 2005, **365**(9463):963-964.
2. Donaldson S, Khetani N, Maniatis G, Stephens D, Wright JG: **Sharing clinical trial results with adolescent idiopathic scoliosis patients**. *J Pediatr Orthop* 2009, **29**(5):467-475.
3. Brealey S, Andronis L, Dennis L, Atwell C, Bryan S, Coulton S, Cox H, Cross B, Fylan F, Garratt A *et al*: **Participants' preference for type of leaflet used to feed back the results of a randomised trial: a survey**. *Trials* 2010, **11**:116.
4. CISCRP: **Perceptions & Insights Study: Report on Study Participant Experiences**. In: *Public and Patient Perceptions of Clinical Research.* Edited by CISCRP. Boston, MA: CISCRP; 2013.
5. Elzinga KE, Khan OF, Tang AR, Fernandez CV, Elzinga CL, Heng DY, Vickers MM, Truong TH, Tang PA: **Adult patient perspectives on clinical trial result reporting: A survey of cancer patients**. *Clin Trials* 2016, **13**(6):574-581.
6. Long CR, Stewart MK, Cunningham TV, Warmack TS, McElfish PA: **Health research participants' preferences for receiving research results**. *Clin Trials* 2016, **13**(6):582-591.
7. Shalowitz DI, Miller FG: **Communicating the results of clinical research to participants: attitudes, practices, and future directions**. *PLoS Med* 2008, **5**(5):e91.
8. Partridge AH, Wolff AC, Marcom PK, Kaufman PA, Zhang L, Gelman R, Moore C, Lake D, Fleming GF, Rugo HS *et al*: **The impact of sharing results of a randomized breast cancer clinical trial with study participants**. *Breast cancer research and treatment* 2009, **115**(1):123-129.
9. Williams SL, Ferrigno L, Maraini G, Rosmini F, Sperduto RD: **A post-trial survey to assess the impact of dissemination of results and unmasking on participants in a 13-year randomised controlled trial on age-related cataract**. *Trials* 2011, **12**:148.
10. Partridge AH, Hackett N, Blood E, Gelman R, Joffe S, Bauer-Wu S, Knudsen K, Emmons K, Collyar D, Schilsky RL *et al*: **Oncology physician and nurse practices and attitudes regarding offering clinical trial results to study participants**. *J Natl Cancer Inst* 2004, **96**(8):629-632.
11. Rigby H, Fernandez CV: **Providing research results to study participants: support versus practice of researchers presenting at the American Society of Hematology annual meeting**. *Blood* 2005, **106**(4):1199-1202.
12. MacNeil SD, Fernandez CV: **Attitudes of research ethics board chairs towards disclosure of research results to participants: results of a national survey**. *Journal of medical ethics* 2007, **33**(9):549-553.
13. Cox K, Moghaddam N, Bird L, Elkan R: **Feedback of trial results to participants: a survey of clinicians' and patients' attitudes and experiences**. *European journal of oncology nursing : the official journal of European Oncology Nursing Society* 2011, **15**(2):124-129.
14. Getz K, Hallinan Z, Simmons D, Brickman MJ, Jumadilova Z, Pauer L, Wilenzick M, Morrison B: **Meeting the obligation to communicate clinical trial results to study volunteers**. *Expert Rev Clin Pharmacol* 2012, **5**(2):149-156.
15. Dixon-Woods M, Jackson C, Windridge KC, Kenyon S: **Receiving a summary of the results of a trial: qualitative study of participants' views**. *Bmj* 2006, **332**(7535):206-210.
16. Fernandez CV, Gao J, Strahlendorf C, Moghrabi A, Pentz RD, Barfield RC, Baker JN, Santor D, Weijer C, Kodish E: **Providing research results to participants: attitudes and needs of adolescents and parents of children with cancer**. *J Clin Oncol* 2009, **27**(6):878-883.
17. Dalal H, Wingham J, Pritchard C, Northey S, Evans P, Taylor RS, Campbell J: **Communicating the results of research: how do participants of a cardiac rehabilitation RCT prefer to be**

informed? *Health expectations : an international journal of public participation in health care and health policy* 2010, **13**(3):323-330.
18. Dorsey ER, Beck CA, Adams M, Chadwick G, de Blieck EA, McCallum C, Briner L, Deuel L, Clarke A, Stewart R *et al*: **Communicating clinical trial results to research participants**. *Archives of neurology* 2008, **65**(12):1590-1595.
19. Aldinger C, Bierer B, Collyar D, Li R, Myers L: **MRCT Return of Results Guidance Document**. In., vol. Version 2.1, Version 2.1 edn: The Multi-regional Clinical Trials Center of Brigham and Women's Hospital and Harvard; 2016.
