# Supplementary material for: Testing approaches to sharing trial results with participants: The Show RESPECT cluster randomised, factorial, mixed methods trial
Source: PLoS Med. 2021 Oct 4;18(10):e1003798. doi: 10.1371/journal.pmed.1003798 (PMC8523080; doi:10.1371/journal.pmed.1003798)
Supplement: S5 Appendix — (PDF) [file pmed.1003798.s009.pdf]

Show RESPECT

Show RESults to Participants Engaged in Clinical Trials

A cluster randomised factorial trial of different modes of communicating results to participants of the ICON8 phase III ovarian cancer trial

ISRCTN: ISRCTN96189403

STATISTICAL ANALYSIS PLAN

Version number 1.0

MRC Clinical Trials Unit

Version date: 15/01/2020

Contacts

Study Statistician: Andrew Copas

Tel: +44 (0)20 7670 4798

Fax: +44 (0)20 7670 4818

Email: mrcctu.ShowRespect@ctu.mrc.ac.uk

This template and all preceding versions will be stored in the Statistical Analysis Master File for this trial held by Andrew Copas

Contents

1. Background and Design 2
1.1 List of Interventions 2
1.2 Overview of data collection 3

2. Outcome measures 4
2.1 Primary outcome measure(s) 4
2.2 Secondary outcome measures 4

3. Data 8
3.1 CRF Forms and variables 8
3.2 Management of datasets 8
3.3 Data completion schedule 9
3.4 Data verification 10
3.5 Data coding 10

4. Definition of terms 10

5. Sample Size Calculations 10

6. Analysis Principles 11
6.1 Intention-to-treat (ITT) or per-protocol or other analysis population? 11
6.2 Approach to multiple interventions in factorial design 12
6.3 Significance level of tests 12
6.4 Adjustment for design factors 12
6.5 Losses to follow-up: handling missing data 12
6.6 Summarising models 12
6.7 Blinding and checking 12

7. Analysis Details 13
7.1 Recruitment and follow-up patterns 13
7.2 Baseline Characteristics 13
7.3 Intervention offer and uptake 13
7.4 Primary analysis 14
7.5 Sensitivity analyses 14
7.6 Subgroup analyses 14
7.7 Adjustment for baseline factors in analysis 14
7.8 Regression diagnostics 14
7.9 Multiple imputation by chained equations (MICE) 15

8. Signatures of Approval 16

1. BACKGROUND AND DESIGN

The Show RESPECT study aims to evaluate the feasibility and effectiveness of different approaches to communicating the results of the progression free survival (PFS) analysis in the ICON8 trial to ICON8 participants. It is a mixed methods study that includes quantitative and qualitative components. The study has a cluster randomised 2 by 2 by 2 factorial design, as shown in Figure 1, with each British ICON8 site acting as a separate cluster.

Figure 1: study schema

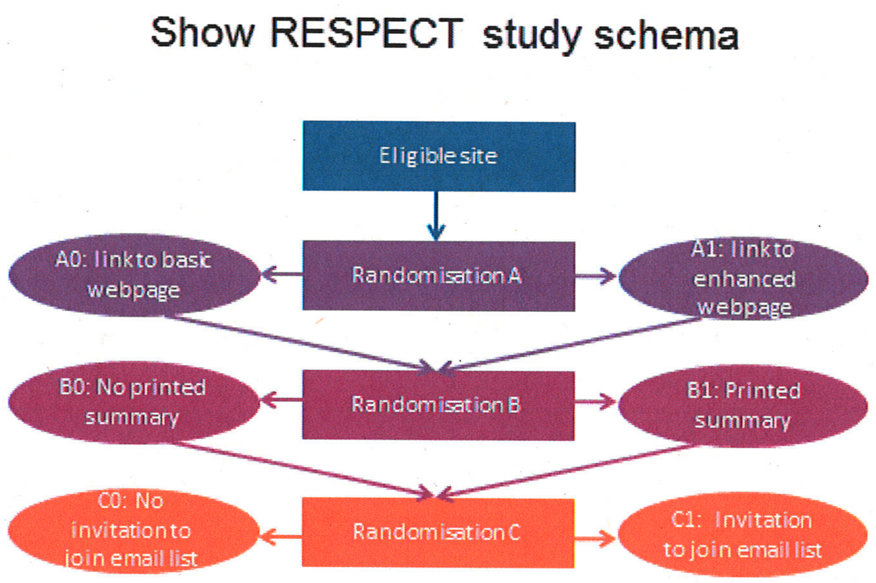

As there is clear evidence that trial participants want to be offered trial results, all participants will have the opportunity to receive the results in some form. The minimum offered is a link to a basic webpage containing the trial results. This basic webpage follows the content and structure recommended in the template in the EU Guidelines on Summaries of Clinical Trial Results for Laypersons https://ec.europa.eu/health/sites/health/files/files/eudralex/vol-10/2017_01_26_summaries_of_ct_results_for_laypersons.pdf. This will act as a control against which to test the enhanced webpage intervention.

Full details of the background to the trial and its design are presented in the protocol.

1.1 List of Interventions

- **Enhanced webpage**

Participants randomised to receive a link to an enhanced webpage were given the URL of a webpage in their End of Study Information Sheet. Each site randomised to this was given a different URL (pointing to the same page), to allow us to monitor uptake by site. The enhanced webpage was not linked from other pages on the website, was not findable via the site navigation, and search engines were discouraged from indexing it, so people will need to know the URL to access it, to reduce crossover. It was up to participants who have been told the URL as to whether they access it.

- **Printed summary**

Participants at sites randomised to use the printed summary were posted the printed summary by their current trial site, if their current address was known, around three weeks after they had been sent the End of Study Information Sheet, unless they had opted out.

- **Invitation to join email list**

Participants randomised to be invited to join an email list were given a URL to sign-up to the email list in their End of Study Information Sheet. The URL takes them to a form where participants can enter their email address onto a secure MailMan database, which was not linked to their trial data.

Further information about the interventions can be found in the protocol.

1.2 Overview of data collection

As part of the study we collected data from trial participants, site staff involved in communicating results, and members of the ICON8 team at the MRC CTU at UCL.

The primary outcome measure (which was collected from patients) is: "How satisfied are you with the way you found out the results of ICON8 (rather than the results themselves)?" This was measured using a Likert scale (1=Very unsatisfied, 2=Somewhat unsatisfied; 3=neither satisfied nor unsatisfied; 4=Somewhat satisfied; 5=Very satisfied).

All British sites of ICON8 will be included, unless they opted out of Show RESPECT.

- **Patient inclusion criteria**
  1. Participant in the ICON8 trial
  2. Currently being followed up at an ICON8 trial site in England, Scotland, Wales and Northern Ireland
  3. Aged 18 years or older

- **Patient exclusion criteria**
  1. Participant has previously informed their site that they do not wish to attend any further visits in relation to the ICON8 trial, or provide any further data (sometimes referred to as 'withdrawal of consent'); participants who previously stopped ICON8 trial treatment earlier than expected but continue in ICON8 follow-up will not be excluded, nor will participants who have reduced follow-up arrangements but still contribute data to the ICON8 trial.
  2. Lost to follow-up from the ICON8 trial
  3. Site staff consider the patient to be too unwell to be contacted about this study

- **Site stratification and randomisation, allocation blinding**

For the purposes of randomisation and data collection sites were stratified by size, i.e. the number of eligible potential participants. The three strata were defined as

1. Low volume sites: sites that have 5 or fewer alive participants
2. Medium volume sites: sites that have 6-12 alive participants
3. High volume sites: sites that have 13 or more alive participants

Sites were randomised in this cluster randomised trial. The randomisation was phased because sites took different times to obtain the necessary approvals, and because it was preferred to wait till there were blocks of size 8 [the number of allocation arms] available. Specifically randomisations were conducted at five distinct time points (five 'phases'), with the numbers of sites included at each phase as follows:

1. 8 small and 8 medium sites [one block of each size]
2. 8 large sites [one block]
3. 4 small and 4 medium sites [randomised jointly as a 'mixed' block]
4. 3 small, 1 medium and 4 large [randomised jointly as a 'mixed' block]
5. 2 small and 1 large [randomised jointly, an 'incomplete block' of final trial sites]

Randomisation was conducted through random permutation within blocks. For the incomplete block at the final fifth phase the allocations were the first three of the eight possible allocations after permutation.

To ensure allocation blinding, although the trial statistician generated the allocations for the blocks and was aware of which clinics featured in each block, a second statistician unaware of these allocations randomly permuted the clinic names within blocks. The allocations and clinic names for each block were then matched together by a third party and revealed to the trial team.

- **Individual participation and data collection**

At trial sites the allocated intervention is offered to all eligible participants, however not all are subsequently approached for data collection, so as to reduce the burden on participants and staff particularly bearing in mind the feature of cluster randomised trials that the marginal information value of each participant declines as cluster size increases. Specifically at small sites all eligible participants are invited to provide outcome data, but at medium sites we aim to collect outcome data from 6 participants and from large sites we aim to collect data from 12.

In medium and large sites the eligible participants were randomly permuted within each site by the trial statistician (using ICON8 trial identifier codes) and the first 6 patients (for medium sites) or 12 patients (for large sites) in the list were invited to provide outcome data. Should some of these participants however not provide outcome data then within each site subsequent (replacement) individuals in the randomly permuted list are approached with the aim of achieving the site target number (6 or 12).

Full details of the background to the trial and its design are presented in the protocol.

2. OUTCOME MEASURES

2.1 Primary outcome measure(s)

The primary outcome measure (which will be collected from patients) is:

"How satisfied are you with the way you found out the results of ICON8 (rather than the results themselves)?"

This will be measured using a Likert scale (1=Very unsatisfied, 2=Somewhat unsatisfied; 3=neither satisfied nor unsatisfied; 4=Somewhat satisfied; 5=Very satisfied).

2.2 Secondary outcome measures

This analysis plan is primarily concerned with the measures from the patient's perspective, but other outcomes are listed here for completeness.

2.2.1 Secondary outcome measures (patient's perspective)

Outcome measure (superscript indicates purpose)	Response options	Outcome type
Did you want to find out the results of ICON8?[1]	yes/no	Binary
Have you heard about the results of the ICON8 trial?[1]	yes/no	Binary
Uptake of the intervention(s) offered[2]	yes/no	Binary (for each intervention offered)
Proportion of participants who wanted to find out the results who reported finding out[3]	n/a	Proportion
Proportion of participants who did not want to find out the results who reported finding out[3]	n/a	Proportion
What were the main reasons you were satisfied or not satisfied[3]	free text	Free text
The information about the trial results told me everything I wanted to know[3]	1=Strongly disagree, 2=slightly disagree, 3=neither agree nor disagree; 4=slightly agree; 5=strongly agree	Ordinal
The ICON8 results were easy to understand[3]	1=Strongly disagree, 2=slightly disagree, 3=neither agree nor disagree; 4=slightly agree; 5=strongly agree	Ordinal
It was easy for me to find out the ICON8 results[3]	1=Strongly disagree, 2=slightly disagree, 3=neither agree nor disagree; 4=slightly agree; 5=strongly agree	Ordinal
I am glad I found out the results[3]	1=Strongly disagree, 2=slightly disagree, 3=neither agree nor disagree; 4=slightly agree; 5=strongly agree	Ordinal
Willingness to take part in research again in future[4]	1=Very unwilling, 2=slightly unwilling, 3=not sure; 4=quite willing; 5=very willing	Ordinal
Likelihood of recommending taking part in a	1=Very unlikely,	Ordinal

clinical trial to friends and family[4]	2=quite unlikely, 3=not sure; 4=quite likely; 5=very likely	
Are there other ways you would have liked to receive the results? If so, how?[3]	yes/no + free text	Binary + free text
Which way of finding out the results did you prefer?[3] (For patients offered more than one approach)	basic webpage, enhanced webpage, printed summary, email, other (+free text)	Categorical
I am glad I found out the results[3]	1=Strongly disagree, 2=slightly disagree, 3=neither agree nor disagree; 4=slightly agree; 5=strongly agree	Ordinal
I regret finding out the results[3]	1=Strongly disagree, 2=slightly disagree, 3=neither agree nor disagree; 4=slightly agree; 5=strongly agree	Ordinal

1. Contextual information
2. Process outcome
3. Measure of intervention effectiveness (in addition to primary outcome)
4. Other

2.2.2 Secondary outcomes (site staff perspective)

Outcome measure	Response options	Outcome type
Site staff's preferred method of communicating the results to participants	Posted, printed summary; email; basic webpage; enhanced webpage; combination of approaches (+ free text); other (+ free text)	Categorical
Concerns with the interventions / process of communicating results	Free text	Free text
Time taken to deliver the interventions (in hours) (and by whom):<br>• End of Study Information Sheet<br>• Printed Summary	0-1; 2-4; 5-7; 8-10; More than 10	Ordinal
Who delivered the interventions?	Research nurse, research practitioner, research radiologist, clinical nurse specialist	Categorical

	Clinician<br><br>Clinical Trial Coordinator or Research Manager<br><br>Data manager<br><br>Trials administrator<br><br>Other (specify)	
Challenges faced implementing the interventions	Free text	Free text
Number of queries received from patients following the results being communicated	1-2; 3-5; 5-10; More than 10	Ordinal
How long it took to deal with a single enquiry, on average (On the CRF we asked how many hours they spent dealing with participant queries, and how many queries they received, so to work out how long it took to deal with a single enquiry we'd have to divide the former by the latter)	Time	Time
Non-staff costs incurred by sites	Monetary value	Numeric
Should intervention(s) they were randomised to become standard practice for the trials they are involved in	yes/no	Binary
Would they prefer to have given participants a different way to find out the results? If so, how and why?	Yes/no + free text	Binary and free text
What would they like to do differently for the next trial they are involved in communicating results for?	Yes/no + Free text	Binary and Free text
What proportion of patients did the End of Study Information Sheet go out to?	Numeric	Proportion
What proportion of patients in sites randomised to the printed summary were sent the printed summary	Numeric	Proportion
How many patients opted out of receiving the printed summary	Numeric	Proportion

2.2.3 Secondary outcomes (CTU perspective)

**Outcome measure**	**Response options**	**Outcome type**
CTU staff's preferred method of communicating the results to participants	Posted, printed summary; email; basic webpage; enhanced webpage; combination	Categorical

	of approaches (+ free text); other (+ free text)	
Concerns with the interventions or process	Free text	Free text
Time taken at CTU for each of the interventions (in hours)	0; 1-3; 4-7; 8-10; More than 10	Ordinal
Cost of each intervention to CTU	Money	Continuous
Challenges faced by CTU in implementing the interventions	Free text	Free text
Number of queries received from participants or sites about the interventions or results	Numeric	Continuous
Would they prefer to have given participants a different way to find out the results? If so, how and why?	Yes/no + free text	Binary and free text
What would they like to do differently for the next trial they are involved in communicating results for?	Free text	Free text
Uptake of webpages by site	Numeric	Proportion

2.2.4 Other secondary outcomes

Cost per participant of each intervention (adding site and CTU costs, and dividing by the number of participants who were offered/received the results that way).

3. DATA

3.1 CRF Forms and variables

Full details of data collection and timing are described in the trial protocol (version 3.0, 20-Aug-2019).

A copy of the CRFs are presented in the protocol and the Trial Master File. Details of the variables are presented as in the metadata which forms part of the Trial Master File.

3.2 Management of datasets

For this study the primary outcome data are obtained from participant self-reports and the responses obtained cannot be subsequently queried with the participants.

The trial database will be locked before the final analysis in accordance with the MRC CTU Database Lock SOP.

Statistical analyses will be performed using the statistical analysis files extracted from the trial database and read into Stata version 15.

All data will be stored in named and dated network folders which are accessible only to the Trial Statisticians.

3.3 Data completion schedule

Data collection type	Method	Timing
Quantitative		
Quantitative data from ICON8 trial participants	Single questionnaire, distributed by site staff, with site-level incentives to increase response rates	To begin at each site 1 month after administration of last intervention (Patient Update Information Sheet sent/ email sent / printed summary sent, depending on randomisation of site) to the last patient at that site. Sites will be reminded of when this is. Aim to complete data collection within 6 months
Quantitative data from ICON8 site staff	Case Report Forms for site staff to complete; one immediately after intervention delivery, one later (more than one set per site allowed, if several people were involved in the process of communicating results)	Data about the process of communicating results will be collected immediately after interventions have been delivered. Data about the response from patients will be collected 2-3 months after administration of last intervention. Aim to complete data collection within 6 months
Quantitative data from MRC CTU trial staff	Case Report Forms for CTU staff to complete; one immediately after intervention delivery, one later (one set per team member involved in dissemination of trial results)	Data about the process of communicating results will be collected immediately after interventions have been delivered. Data about the response from patients and sites will be collected 2-3 months after administration of last intervention.
Qualitative		
Qualitative data from ICON8 trial participants	Interviews with trial participants, conducted by Annabelle South	To begin approximately two months after site has delivered intervention/Patient Update Information Sheet
Qualitative data from ICON8 site staff	Interviews with site staff, conducted by Annabelle South	To begin two months after site has delivered last intervention

3.4 Data verification

For this study the primary outcome data are obtained from participant self-reports and the responses obtained cannot be subsequently queried with the participants.

Data verification, consistency and range checks will be performed, as appropriate, when the analysis is performed (and when the datasets for analysis are constructed). All variables will be examined for unusual, outlying, unlabelled or inconsistent values.

Any problems with trial data will be queried with the Trial Managers, Data Managers, or statisticians, as appropriate. If possible, data queries will be resolved, although it is accepted that due to administrative reasons and data availability a small number of problems will continue to exist. This will be minimised.

3.5 Data coding

A list of variable coding can be found S:\MRCCTU_Show_Respect\6.0 Data Management\6.2 Metadata

4. DEFINITION OF TERMS

Term	Definition
Patient update information sheet	Document sent to patients to inform they the results are available, and how to access them
ICON8	International Collaborative Ovarian Neoplasm 8 trial

5. SAMPLE SIZE CALCULATIONS

The calculation presented in section 7.3 of the trial protocol assumed that at least 37 sites would be randomised, which was the number who had agreed at the time (12 large, 12 medium, and 13 small). Ultimately 43 sites were randomised, 13 large, 13 medium, and 17 small.

We assume, as in the protocol, that, given the range of site sizes across the strata, the coefficient of variation of the number of outcomes collected per site to be fairly substantial and take its value 0.6. Little information concerning the likely intracluster correlation coefficient (ICC) for the primary outcome is available so we consider values between 0.01 (low) and 0.05 (moderate).

The primary outcome is ordinal but for simplicity, because of lack of knowledge of its likely distribution and to be scientifically conservative, we consider it as a binary outcome for our power calculations. We consider that even if outcomes are generally positive that no more than 80% of respondents will report being very satisfied and similarly that even if outcomes are negative that no more than 80% will indicate being very dissatisfied. As such we expect sufficient variation that we can examine improvement in satisfaction where the more satisfied group has prevalence between 20 and 80%. We consider power to detect an effect for any of the three interventions, for simplicity considering each in turn i.e. effectively conducting a power calculation for each intervention assuming the other two have no effect. We acknowledge that should multiple interventions have effects then the power will vary somewhat from that presented here.

The protocol states that an average of 6 primary outcomes per site will be collected, and hence outcomes from at least 222 participants in total. At a low value of the ICC that sample size (considering 18 sites with and without an intervention) provides 80% power to detect an increase from 20 to 38% in the more satisfied group, or from 50 to 70% or from 80 to 93%. Should the ICC be moderate at 0.05 then that sample size provides 80% power to detect an increase from 20 to 40% or 50 to 72% or 80 to 95%.

We next consider power based on the final number of 43 sites randomised, and also for a smaller number of outcomes per site on average to better match the sample size now anticipated. We assume an average of 4 outcomes per site (172 in total) and consider 21 sites with and without an intervention. At a low value of the ICC that sample size provides 80% power to detect an increase from 20 to 40% in the more satisfied group, or from 50 to 71% or from 80 to 95%. Should the ICC be moderate at 0.05 then that sample size provides 80% power to detect an increase from 20 to 42% or 50 to 73% or 80 to 95%.

No calculations have been made for the secondary outcomes.

6. ANALYSIS PRINCIPLES

6.1 Intention-to-treat (ITT) or per-protocol or other analysis population?

The primary analysis will be conducted under the ITT principle, ignoring how well clusters may have implemented their allocated interventions. All allocated interventions are believed to have been offered to all eligible participants in all clusters. At the time of writing it is believed that almost all participants at sites randomised to the printed summary will receive the printed summary, as very few opted out. Conversely very few if any participants have opted in to an email group. However for the webpage intervention, and only for the primary outcome, we will conduct an 'as treated' comparison between enhanced and basic webpage restricted to those who report viewing the webpage.

However the primary outcome is only defined for participants who have received the ICON8 trial results, and hence analysis for this outcome is restricted to participants who also report receiving the ICON8 results. For this reason we describe the primary analysis as following modified-ITT (mITT). Most other secondary outcomes intended to measure the effectiveness of the interventions (see 2.2.1) are similarly only defined for participants who have received the ICON8 results. For the outcome 'report of finding out the ICON8 results', analysis will be presented separately among participants who report they wanted to find the results out, and among participant who report they did not. To assess the overall effect of the intervention, it is important to interpret the results of the primary analysis alongside these results concerning the possible effect of the interventions on whether participants found out the ICON8 results.

In this trial setting participant's health may be poor and may deteriorate before interventions are received or between intervention exposure and follow-up by questionnaire. Participants who die or become too sick to complete a questionnaire are not considered 'eligible' for data collection or analysis (see eligibility criteria), and so not considered as missing data.

6.2 Approach to multiple interventions in factorial design

The prior assumption in the trial design is that there will not be any important interactions between the three interventions. Hence the planned primary analysis is of the main effects of each intervention adjusting for the others. However for primary outcome we shall also test each of the three two-way interactions, and also report the effect of each of seven intervention combinations relative to control. In the event of statistically significant and qualitatively important interaction(s) we shall change the analysis approach to report as primary analysis for the primary outcome the effect of the three (or even seven) intervention combinations relative to control, as determined by the interaction findings.

6.3 Significance level of tests

We consider that in testing each of the three interventions distinct research questions are answered and hence we do not propose to change from the standard 5% level.

6.4 Adjustment for design factors

To reflect the design we shall in our analysis adjust for clinic size stratum, and also early [first two phases] vs. later randomisation phases.

6.5 Losses to follow-up: handling missing data

It is anticipated that a non-negligible proportion of participants invited to complete an outcome questionnaire will not return it. We shall not apply any procedure to impute outcomes in this case, though bias may be reduced through adjustment for baseline factors (see section 7.7). It is unknown whether more than a handful of individuals will return an outcome questionnaire with the primary outcome omitted but providing related information that could be used to impute the primary outcome. If more than 10% of individuals who return an outcome questionnaire omit to complete the primary outcome then we shall investigate whether and how to perform multiple imputation for the primary outcome (see 7.9). Otherwise we shall conduct an available case analysis. If multiple imputation is conducted then analysis based on the imputed datasets will be considered primary, though the available case analysis will also be presented.

6.6 Summarising models

We shall estimate and present effect measures for the interventions based on regression models. The primary and many other outcomes are ordinal based on Likert scales. We naturally propose ordinal logistic regression, but will revert to binary logistic if the assumptions of proportional odds are not met, see model diagnostics section. All models will include random effects for clinic.

6.7 Blinding and checking

The primary endpoint analysis will be independently (double) programmed by a statistician that was not involved in the analysis. Secondary outcomes will not be double programmed. Both analysts however cannot be simply blinded to allocation as they have been involved in the randomisation process. However we shall conduct some preliminary analysis tasks using a dataset in which individual and cluster identifiers, and allocation identifiers, have been removed. Firstly we will perform initial consistency checks and, where possible, assessment of the intended responses for trial outcomes where these self-reports are inconsistent. Secondly we will consider merging response categories for our ordinal outcomes (see section 7.4.1 for

details of process). Thirdly we will examine co-linearity between baseline factors (see later for details) and examine category cell sizes and make final decisions about exactly how these factors can be included in regression models for trial outcomes, and exactly which groups can be used in our subgroup analyses. Fourthly we will decide whether and how to conduct multiple imputation for the primary outcome.

7. ANALYSIS DETAILS

7.1 Recruitment and follow-up patterns

A CONSORT diagram will be presented. This will reflect that not all those who participated in the trial were asked to provide outcome data, and will also reflect that at larger sites there was a process of 'replacing' individuals who were asked to provide data but subsequently proved ineligible (e.g. too sick) or who did not respond within a suitable time period.

Shell consort diagram

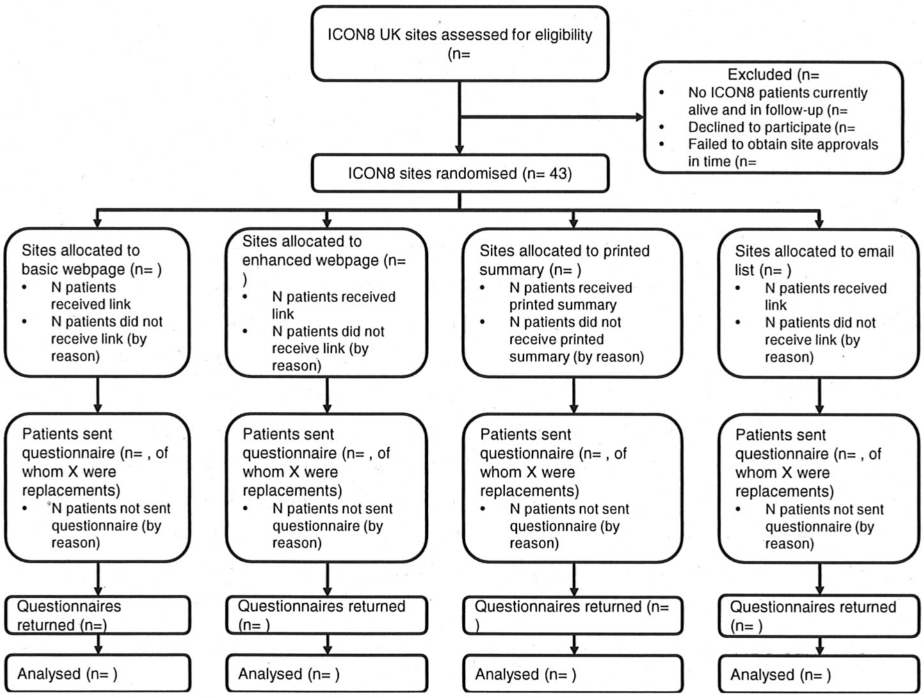

7.2 Baseline Characteristics

These will be presented 'by margin', i.e. comparing those who were and were not allocated to each intervention in turn, rather than by each of the 8 allocation arms separately. We shall also present the baseline tables in two ways – firstly for all eligible participants at the trial sites (excluding those known to have died or be too sick) and secondly for participants included in the primary analysis (i.e. eligible participants who provide outcome). For the first table we shall present characteristics recorded in ICON8 such as age and ICON8 trial arm. For the second table additional self-reported factors are available and will be presented including educational attainment, first language English, and internet use.

7.3 Intervention offer and uptake

These will be reported 'by margin'. Instances of the incorrect intervention offered, if any, will be reported. The self-reported uptake of each margin amongst those (correctly) offered will be reported for the webpage and email group, for the postal information sheet we will assume

this was received unless the participant opted out of the sheet. This is because we feel this is a more reliable way to present uptake for this intervention than self-report.

7.4 Primary analysis

- ### 7.4.1 Primary outcome

Ordinal random effects logistic regression will be used unless its assumptions are clearly violated (see section 7.8). The response categories will be merged for the regression analysis in the event of very low reporting of one or more categories (<5% of responses). If this occurs the lowest response category will be merged with the adjacent category that has the lowest response and the process repeated until all categories have >5% of responses. All decisions about merging of the response categories will be taken based on an initial dataset without cluster or allocation identifiers.

- ### 7.4.2 Secondary outcomes

Ordinal random effect logistic regression will be used for ordinal outcomes, with the same process for merging small response categories described for the primary outcome. Other secondary outcomes are binary so standard logistic regression will be used.

7.5 Sensitivity analyses

An as treated analysis for the webpage intervention is planned (see section 6.1). If multiple imputation is applied to deal with item non-response for the primary outcome then an available case analysis will also be conducted.

7.6 Subgroup analyses

For the primary outcome only, we will conduct subgroup analyses by age group (<=70 vs. 71+), arm in ICON8 trial, first language English, education group [graduate vs. not], and reported internet use [daily vs. not]. These proposed groupings are subject to cell size. For each subgroup analysis the effect of each intervention within subgroups will be presented [there are two subgroups per factor] and an interaction test will be conducted by including an interaction term between factor and intervention in the regression model (see section 6.6 for model details). The interaction term will be based on the two subgroups, except for age where the term will be based on treating age as continuous. These subgroup analyses will be conducted for each of the three interventions 'by margin'. This means that interaction terms are created for each intervention separately and each of the three terms is tested by itself in a model that contains main effects for the other two interventions.

7.7 Adjustment for baseline factors in analysis

Besides presenting estimates adjusted for the design factors as fixed effects (see section 6.4) we shall also present effect estimates adjusted for age (continuous – linear), education (graduate vs. not), and internet use (daily vs. less). Since effect estimates for each of the three interventions come from the same combined model, they will all be adjusted for the same factors.

7.8 Regression diagnostics

The key aspect of model fit we need to address is the assumption of proportional odds that underlies the ordinal logistic regression analysis we intend to apply to the primary outcome. Formal tests are available, though these may lack power in modest datasets and conversely may detect unimportant deviation from proportional odds when datasets are large. We propose therefore to base the assessment of whether ordinal regression is appropriate by inspecting the proportion of participants reporting each of the ordered response categories for each intervention in turn 'by margin'. The key deviation from proportional odds we shall look

for is that an intervention affects the primary outcome by increasing reporting at both extremes – i.e. both increased satisfaction and dissatisfaction.

In the event of clear violation of proportional odds we shall instead apply binary logistic regression to two outcomes derived from the primary outcome and dependent on cell size. Subject to sufficient cell size (>10% of participants) we shall fit two models for the outcomes (i) indifferent or agree, and (ii) strongly agree.

Some complexity arises if the proportional odds assumption is clearly violated for some but not all of the three interventions. If the assumption is violated clearly for one intervention and likely violated for the others, or if it is clearly violated for two outcomes, then we shall apply binary logistic regression as described above. However if the assumption is clearly violated for one outcome with no evidence of violation for another then we shall apply a partial proportional odds model. This will be implemented through user-written gologit2 software in Stata. This will lead to a single intervention effect for interventions that do not clearly violate proportional odds and to two intervention effects (odds ratios) for those that do. Using this software marginal effects are generated, giving a population average interpretation.

If proportional odds is clearly violated for an intervention with regard to the primary outcome then it will also be assessed whether it is clearly violated for the ordinal secondary outcomes but otherwise that assessment will not be made and ordinal logistic regression will be applied.

7.9 Multiple imputation by chained equations (MICE)

As described in section 6.5, we shall consider applying multiple imputation should >10% of participant outcome questionnaires returned have omitted the primary outcome. We do not intend to impute the response concerning whether the participant has received the results of the ICON8 trial, participants who have missed that item are excluded. The imputation model will be fitted only to participants who report they have obtained the ICON8 results. The ideal predictors to use in imputation are the secondary outcomes that relate to the effectiveness of the intervention (see 2.2.1) and reporting of the uptake of the interventions. However if these predictors are also generally missing whenever the primary outcome is missing then we shall not perform multiple imputation and present only an available case analysis. If they are generally observed but sporadically missing (to be expected) then we shall use chained equations, which imputes these outcomes as well as the primary outcome. Due to the limited data per arm across the eight allocation arms, imputation will not be conducted separately by allocation arm, but indicators of the three interventions (main effects) will be included as predictors in the imputation model. Although the data are clustered by site this complexity will not be incorporated into the imputation, and the standard mi functions in Stata version 15 will be used.

Multiple imputation will be considered to impute missing values for the baseline adjustment factors used in analysis of our primary outcome, subject to the missing data pattern. If factors have very low missing data (<2%) then imputation will not be considered, if missing data is >10% then the factor will not be included in the regression model for the primary outcome. However if missing data is intermediate and sporadic then multiple imputation will be used for the adjustment factors. If it is decided to impute the primary outcome also then through chained equations the primary and secondary outcomes will be imputed simultaneously together with the adjustment factors.

8. SIGNATURES OF APPROVAL

Date: 15 January 2020

Version: 1.0

Signatures

Name	Trial Role	Signature	Date
Annabelle South	*CI (on behalf of all TMG's)**	Annabelle South	15/01/2020
Andrew Copas	Senior Statistician/Scientist	Andrew Copas	15/1/2020
