# Supplementary material for: Testing approaches to sharing trial results with participants: The Show RESPECT cluster randomised, factorial, mixed methods trial
Source: PLoS Med. 2021 Oct 4;18(10):e1003798. doi: 10.1371/journal.pmed.1003798 (PMC8523080; doi:10.1371/journal.pmed.1003798)
Supplement: S1 Table — (DOCX) [file pmed.1003798.s010.docx]

# S1 Table: Description of the interventions tested in Show RESPECT

| Brief name | **Basic webpage** | **Enhanced webpage** | **Mailed Printed summary** | **Email list Invitation** |
| --- | --- | --- | --- | --- |
| Why | European Parliament Regulation (EU) No 536/2014 Article 37 (4) requires sponsors to  provide summary results of clinical trials in a format understandable to laypersons. These summaries will be made available in a new EU database [1]. The regulation specifies 10 items that should be included in the summary. The ‘friendly’ version of these items has been used to structure the basic webpage content, as this is the format in which lay summaries will be available for EU trials once the database is launched, and is therefore the minimum that will be available to participants. The content was written using principles of plain English. | The enhanced webpage was designed to take advantage of some of the opportunities afforded by webpages to include different sorts of content in addition to static text. It was also written specifically for participants, rather than lay audiences in general, using a structure adapted from the MRCT guidance document [2]. It was hypothesised that adding these additional elements, and restructuring the results summaries specifically for participants, would increase participant satisfaction with how the results were communicated.  The content was written using principles of plain English. | As the population of women taking part in ICON8 had an average age of >60, it was thought that lack of computer literacy might act as a barrier to some patients accessing the results via webpage or email. A printed summary might avoid these challenges.  Previous studies have seen low uptake of results when participants have to opt-in to receive them, so it was decided to make the printed summaries opt-out.  The text was also written specifically for participants, rather than lay audiences in general using a structure adapted from the MRCT guidance document[2]. It was hypothesised that providing the results in printed format to all participants who did not opt out would improve satisfaction compared to not offering a printed summary.  The content was written using principles of plain English. | The email list was designed to offer participants the opportunity to receive content similar to the enhanced webpage directly to their inbox. This was particularly relevant for responses to any frequently asked questions, to save participants having to regularly check the webpage for updates. The content of the results email was the same as the enhanced webpage, minus the video. The content of subsequent emails was the same as the frequently asked question updates of the enhanced webpage. |
| Materials | Participants received a printed update information sheet thanking them for taking part in the trial, telling them that the results were available, and how to access them (including a link to the basic webpage).  The basic webpage contained sections on:   1. Study name 2. Who sponsored this study? 3. General information about the study 4. What patients were included in this study? 5. Which medicines were studied? 6. What were the side effects? 7. What were the overall results of the study? 8. How has this study helped patients and researchers? 9. Are there plans for further studies? 10. Where can I find further information about this study?   The web page was be laid out with clear headings. The body text was Arial 12pts, black against a white background. Section 10 of the webpage contained a link to the entry on the clinical trial register. Text was written using the principles of Plain English.  <https://www.ctu.mrc.ac.uk/studies/all-studies/i/icon8/results-of-the-icon8-trial-1/> | Participants received a printed update information sheet thanking them for taking part in the trial, telling them that the results were available, and how to access them (including a link to the enhanced webpage).  The enhanced webpage contained the following sections:   1. Thank you 2. What was the study about? 3. Why was the study needed? 4. Who took part in the study? 5. How was the study carried out? 6. What did the study find? 7. How sure can we be about these results? 8. What do these results mean?  - What do these results mean for you? - What do these results mean for other people?  1. What difference will these results make? 2. Thank you 3. Further information 4. Support 5. Frequently asked questions   In addition to written content, the enhanced webpage contained a short video of a trial clinician explaining the results. The enhanced webpage also contained two diagrams.  The frequently asked questions section contained a link to an online form where participants could post anonymous questions about the results, to be answered in that section of the webpage.  The web page was be laid out with clear headings. The body text was Arial 12pts, black against a white background.  Text was written using the principles of Plain English. Section 11 contained links to the Cancer Research UK’s page about ICON8, the clinical trial register entry for ICON8, information about ovarian cancer from ovarian cancer charities,  <https://www.ctu.mrc.ac.uk/studies/all-studies/i/icon8/results-of-the-icon8-trial/> | Participants received a printed update information sheet thanking them for taking part in the trial, telling them that the results were available, and how to access them. This included a link to whichever webpage they had been randomised to. They were also told that they would be sent a printed summary of the results in three weeks time, unless they let their study team know that they did not wish to receive this.  The printed summary contained the following sections:   1. Thank you 2. What was the study about? 3. Why was the study needed? 4. Who took part in the study? 5. How was the study carried out? 6. What did the study find? 7. How sure can we be about these results? 8. What do these results mean?    1. What do these results mean for you?    2. What do these results mean for other people? 9. What difference will these results make? 10. Thank you 11. Further information   The content of the printed summary was written following the principles of Plain English. The text  of sections 2-10 was identical to that of the enhanced webpage. The further information section  contained information on relevant patient helplines, and the link to the trial registry entry.  The printed summary were laid out with clear headings, plenty of white space, and the body text was Arial 12pts, black against a white background. It used graphics to illustrate key points. It  was four A4 pages long. It was professionally printed on 150gsm paper. | Participants received a printed update information sheet thanking them for taking part in the trial, telling them that the results were available, and how to access them. This included a URL to a form where participants could enter their email address onto a secure MailMan database, which was not linked to their trial data.  The email updates were designed using a MailChimp template that works well in different email platforms, including mobile phones.  The email main results email contained the following sections:   1. Thank you 2. What was the ICON8 trial about? 3. Why was the ICON8 trial needed? 4. Who took part in the ICON8 trial? 5. How was the ICON8 trial carried out? 6. What did the ICON8 trial find? 7. How sure can we be about these results? 8. What do these results mean?    1. What do these results mean for you?    2. What do these results mean for other people? 9. What difference will these results make? 10. Thank you 11. Further information 12. Any questions? 13. Support 14. Tell us what you think about this email   Subsequent emails included answers to questions sent in by participants, and links to the previously emails.  The content of the emails were written following the principles of Plain English. The email will be laid out with clear headings, plenty of white space, and the body text will be Arial 12pts, black against a white background. |
| Procedures | The Patient Update Information Sheet was sent to all participants at sites randomised to the printed summary, giving them the link to the webpage. | The Patient Update Information Sheet was sent to all participants at sites randomised to the printed summary, giving them the link to the webpage.  Questions about the results could be submitted anonymously via an online form using the Opinio survey system. When questions were received, answers were drafted and posted onto the webpage within three weeks. | The Patient Update Information Sheet was sent to all participants at sites randomised to the printed summary, telling them they would be sent the printed summary in three weeks unless they opted out. The printed summary was then sent to participants who had not opted out. Most summaries were sent by post, but in a few cases, where participants had a clinic appointment near the time the summary was planned to be sent, they were given it in person. | The Patient Update Information Sheet was sent to all participants at sites randomised to the printed summary, giving them the link to sign up to the email list.  When participants signed up to the email list, they were sent an email, confirming their subscription and telling them how they can unsubscribe at any time.  The first email with a summary of results (using the same written content as the enhanced webpage,) was sent 1 month after the first sites randomised had received the Patient Update Information Sheets, to allow them time to distribute them to participants, and for participants to sign up.  Questions about the results could be submitted anonymously via an online form using the Opinio survey system. Update emails were sent out with answers to any frequently asked questions that have been received since the previous email, and any updates.  Participants who signed up to the email list after the first email had been sent were sent a welcome email with a link to online copies of any email(s) that have previously been sent. |
| Who provided | The patient update information sheets were provided by staff at the patient’s trial site. This was usually a research nurse or trial administrator. The webpages were hosted on the MRC Clinical Trials Unit at UCL’s website. | The patient update information sheets were provided by staff at the patient’s trial site. This was usually a research nurse or trial administrator. The webpages were hosted on the MRC Clinical Trials Unit at UCL’s website. | The patient update information sheets and printed summaries were provided by staff at the patient’s trial site. This was usually a research nurse or trial administrator. The webpages were hosted on the MRC Clinical Trials Unit at UCL’s website. | The patient update information sheets were provided by staff at the patient’s trial site. This was usually a research nurse or trial administrator. The email address that the emails were sent from was [mrcctu.icon8-results@ucl.ac.uk](mailto:mrcctu.icon8-results@ucl.ac.uk) |
| Modes of delivery | For most participants the patient update information sheet was delivered by post to the participants’ home address. For a few, where clinic visits coincided with when the documents were due to be sent, they were given in person. The webpage was then available on the internet. | For most participants the patient update information sheet was delivered by post to the participants’ home address. For a few, where clinic visits coincided with when the documents were due to be sent, they were given in person. The webpage was then available on the internet. | For most participants the patient update information sheet and printed summary were delivered by post to the participants’ home address. For a few, where clinic visits coincided with when the documents were due to be sent, they were given in person. | For most participants the patient update information sheet was delivered by post to the participants’ home address. For a few, where clinic visits coincided with when the documents were due to be sent, they were given in person. Sign-up to the email list was via a webpage. Emails were then sent to the people’s email address. |
| Where the intervention took place | At participants’ homes. | At participants’ homes. | At participants’ homes. | At participants’ homes. |
| When and how much | One Patient Update Information Sheet (2 pages) was sent.  Participants could then access the webpage as often as they liked, for as long as they liked. | One Patient Update Information Sheet (2 pages) was sent.  Participants could then access the webpage as often as they liked, for as long as they liked. | One Patient Update Information Sheet (2 pages) was sent.  For patients who did not opt out, a four page printed summary was sent three weeks later. | One Patient Update Information Sheet (2 pages) was sent. Patients who signed up for the email list were then sent a confirmation email.  The first email with a summary of results (using the same written content as the enhanced webpage,) was sent 1 month after the first sites randomised had received the Patient Update Information Sheets, to allow them time to distribute them to participants, and for participants to sign up.  Update emails were sent out with answers to any frequently asked questions that have been received since the previous email, and any updates.  Participants who signed up to the email list after the first email had been sent were sent a welcome email with a link to online copies of any email(s) that have previously been sent. |
| Tailoring | Some sites sent a personalised cover letter or compliments slip with the Patient Update Information Sheet, giving their contact details for if patients had any questions about the results. Some sites phoned patients to tell them they would be sending the Patient Update Information Sheet. Some sites did no tailoring. | Some sites sent a personalised cover letter or compliments slip with the Patient Update Information Sheet, giving their contact details for if patients had any questions about the results. Some sites phoned patients to tell them they would be sending the Patient Update Information Sheet. Some sites did no tailoring. | Some sites sent a personalised cover letter or compliments slip with the Patient Update Information Sheet and Printed Summary, giving their contact details for if patients had any questions about the results. Some sites phoned patients to tell them they would be sending the Patient Update Information Sheet and Printed Summary. Some sites did no tailoring. | Some sites sent a personalised cover letter or compliments slip with the Patient Update Information Sheet, giving their contact details for if patients had any questions about the results. Some sites phoned patients to tell them they would be sending the Patient Update Information Sheet. Some sites did no tailoring. |
| Modifications | Sites were not able to modify the Patient Update Information Sheet or webpage. The content of the webpage remained unchanged. | Sites were not able to modify the Patient Update Information Sheet or webpage. The webpage was updated with answers to questions received via the online form. | Sites were not able to modify the Patient Update Information Sheet or Printed Summary. The content of the Printed Summary remained the same throughout the course of the study. | Sites were not able to modify the Patient Update Information Sheet or email. Additional emails were sent based on questions received from participants. |
| How well | All eligible patients (with one exception) at sites randomised to the basic webpage were sent the Patient Update Information Sheet which contained the link to the basic webpage. The link to the basic webpage for two sites was broken for a week in February 2019 (affecting up to 4 patients). | All eligible patients at sites randomised to the enhanced webpage were sent the Patient Update Information Sheet which contained the link to the enhanced webpage. The link to the enhanced webpage for two sites was broken for 10 days in February 2019 (affecting up to 5 patients). | All eligible patients at sites randomised to the Printed Summary were sent the Patient Update Information Sheet, which informed them they would be sent the Printed Summary in three weeks time unless they opted out. Three patients opted out of receiving the Printed Summary. All other eligible patients were sent the Printed Summary. | All eligible patients at sites randomised to the Email List Invitation were sent the Patient Update Information Sheet which contained the link to sign up to the email list. |

1. Expert group on clinical trials for the implementation of regulation (EU) No 536/2014 on clinical trials on medicinal products for human use, *Summaries of Clinical Trial Results for Laypersons*. 2018, European Commission: Brussels, Belgium.

2. Aldinger, C., et al., *MRCT Return of Results Guidance Document*. 2016, The Multi-regional Clinical Trials Center of Brigham and Women's Hospital and Harvard.
