# Supplementary material for: Testing approaches to sharing trial results with participants: The Show RESPECT cluster randomised, factorial, mixed methods trial
Source: PLoS Med. 2021 Oct 4;18(10):e1003798. doi: 10.1371/journal.pmed.1003798 (PMC8523080; doi:10.1371/journal.pmed.1003798)
Supplement: S2 Table — (DOCX) [file pmed.1003798.s011.docx]

# S2 Table: Baseline characteristics of all eligible participants at trial sites

(NB. Not all of these participants were sent the questionnaire – see protocol for further details).

|  | **Webpage** | | **Mailed Printed Summary** | | **Email List Invitation** | |
| --- | --- | --- | --- | --- | --- | --- |
|  | **Basic Webpage**  **n (%)** | **Enhanced Webpage**  **n (%)** | **No Mailed Printed Summary**  **n (%)** | **Mailed Printed Summary**  **n (%)** | **No invitation**  **n (%)** | **Invitation**  **n (%)** |
| **Age** |  |  |  |  |  |  |
| Mean (IQR) | 67 (61-74) | 66 (58-73) | 66 (59-73) | 67 (60-74) | 67 (61-74) | 66 (59-73) |
| ≤70 years | 115 (58%) | 121 (63%) | 125 (62%) | 111 (60%) | 90 (57%) | 146 (63%) |
| > 70 years | 82 (42%) | 71 (37%) | 78 (38%) | 75 (40%) | 68 (43%) | 85 (37%) |
| **ICON8 arm** |  |  |  |  |  |  |
| A (control) | 58 (29%) | 67 (35%) | 63 (31%) | 62 (33%) | 50 (32%) | 75 (32%) |
| B | 73 (37%) | 62 (32%) | 72 (35%) | 63 (34%) | 53 (34%) | 82 (35%) |
| C | 66 (34%) | 63 (33%) | 68 (33%) | 61 (33%) | 55 (35%) | 74 (32%) |
