# Supplementary material for: Testing approaches to sharing trial results with participants: The Show RESPECT cluster randomised, factorial, mixed methods trial
Source: PLoS Med. 2021 Oct 4;18(10):e1003798. doi: 10.1371/journal.pmed.1003798 (PMC8523080; doi:10.1371/journal.pmed.1003798)
Supplement: S3 Table — (DOCX) [file pmed.1003798.s012.docx]

# S3 Table: Subgroup analyses (primary outcome only)

|  | **Very unsatisfied n (%)** | **Quite unsatisfied**  **n (%)** | **Neither satisfied nor unsatisfied**  **n (%)** | **Quite satisfied**  **n (%)** | **Very satisfied**  **n (%)** | **Unadjusted odds ratio**  **(95% CI) p-value** | | | **Adjusted odds ratio**  **(95% CI) p-value** | | |
| --- | --- | --- | --- | --- | --- | --- | --- | --- | --- | --- | --- |
|  |  |  |  |  |  | **Enhanced vs Basic Webpage** | **Mailed Printed summary vs No Mailed Printed Summary** | **Email List Invitation vs No Email List Invitation** | **Enhanced vs Basic Webpage** | **Mailed Printed summary vs No Mailed Printed Summary** | **Email List Invitation vs No Email List Invitation** |
| **Age** | | | | | | | **Interaction test:** | | p=0.654 | p=0.112 | p=0.926 |
| ≤70 years old | 8 (9) | 5 (6) | 9 (11) | 25 (29) | 38 (45) | 1.39 (0.61 to 3.19) p=0.437 | 2.66 (1.17 to 6.04) p=0.019 | 0.84 (0.36 to 1.94) p=0.686 | 1.40 (0.58 to 3.38) p=0.447 | 2.26 (0.97 to 5.26) p=0.059 | 1.02 (0.42 to 2.46) p=0.970 |
| >70 years old | 4 (7) | 7 (12) | 8 (13) | 15 (25) | 26 (43) | 1.48 (0.56 to 3.93) p=0.428 | 4.69 (1.66 to 13.20) p=0.003 | 2.59 (0.89 to 7.51) p=0.079 | 1.74 (0.63 to 4.86) p=0.286 | 4.94 (1.73 to 14.09) p=0.003 | 2.30 (0.77 to 6.86) p=0.136 |
| **Arm in ICON8 trial** | | | | | | | **Interaction test:** | | p=0.424 | p=0.543 | p=0.557 |
| Standard treatment | 3 (8) | 2 (5) | 5 (13) | 10 (26) | 18 (47) | 0.86 (0.24 to 3.11) p=0.823 | 1.76 (0.48 to 6.44) p=0.396 | 2.34 (0.62 to 8.87) p=0.209 | 0.59 (0.14 to 2.44) p=0.467 | 1.78 (0.46 to 6.84) p=0.403 | 1.96 (0.41 to 9.38) p=0.399 |
| Dose fractionated paclitaxel | 4 (8) | 4 (8) | 7 (13) | 13 (25) | 24 (46) | 2.22 (0.32 to 15.37) p=0.417 | 10.35 (1.29 to 83.07) p=0.028 | 1.22 (0.21 to 7.11) p=0.826 | 2.37 (0.35 to 16.20) p=0.380 | 12.18 (1.30 to 114.21) p=0.029 | 1.07 (0.18 to 6.38) p=0.942 |
| Dose fractionated carboplatin & paclitaxel | 5 (9) | 6 (11) | 5 (9) | 17 (31) | 22 (40) | 2.47 (0.86 to 7.06) p=0.092 | 5.51 (1.77 to 17.08) p=0.003 | 0.91 (0.31 to 2.68) p=0.862 | 2.72 (0.92 to 8.00) p=0.069 | 5.38 (1.68 to 17.28) p=0.005 | 0.96 (0.31 to 3.03) p=0.949 |
| **First language** | | | | | | |  |  |  |  |  |
| Not English | 0 (0) | 0 (0) | 0 (0) | 3 (75) | 1 (25) | - | - | - | - | - | - |
| English | 12 (9) | 12 (9) | 16 (12) | 37 (27) | 62 (45) | - | - | - | - | - | - |
| **Education** | | | | | | | **Interaction test:** | | p=0.520 | p=0.930 | p=0.951 |
| Less than degree level | 10 (9) | 10 (9) | 14 (13) | 23 (21) | 51 (47) | 1.47 (0.71 to 3.04) p=0.297 | 2.92 (1.40 to 6.08) p=0.004 | 1.39 (0.67 to 2.93) p=0.378 | 1.54 (0.74 to 3.20) p=0.252 | 2.91 (1.40 to 6.08) p=0.004 | 1.40 (0.65 to 3.00) p=0.388 |
| Graduate or above | 2 (5) | 2 (5) | 3 (8) | 17 (28) | 13 (35) | 2.20 (0.48 to 10.20) p=0.313 | 5.01 (1.19 to 21.06) p=0.028 | 1.89 (0.46 to 7.79) p=0.377 | 4.14 (0.70 to 24.60) p=0.118 | 2.70 (0.58 to 12.53) p=0.204 | 5.55 (0.89 to 34.40) p=0.066 |
| **Reported internet/email use** | | | | | | | **Interaction test:** | | p=0.955 | p=0.104 | p=0.662 |
| Less than daily | 4 (7) | 6 (11) | 7 (13) | 17 (31) | 21 (38) | 1.83 (0.56 to 5.95) p=0.317 | 8.18 (1.93 to 34.71) p=0.004 | 0.87 (0.23 to 3.21) p=0.829 | 1.82 (0.55 to 6.04) p=0.331 | 9.09 (2.04 to 40.42) p=0.004 | 0.73 (0.19 to 2.86) p=0.653 |
| Daily | 8 (9) | 6 (7) | 10 (11) | 22 (25) | 43 (48) | 1.65 (0.73 to 3.75) p=0.232 | 1.82 (0.80 to 4.14) p=0.151 | 1.42 (0.64 to 3.16) p=0.393 | 1.61 (0.70 to 3.68) p=0.259 | 1.74 (0.76 to 3.98) p=0.189 | 1.51 (0.67 to 3.44) p=0.321 |
