# Supplementary material for: Testing approaches to sharing trial results with participants: The Show RESPECT cluster randomised, factorial, mixed methods trial
Source: PLoS Med. 2021 Oct 4;18(10):e1003798. doi: 10.1371/journal.pmed.1003798 (PMC8523080; doi:10.1371/journal.pmed.1003798)
Supplement: S4 Table — (DOCX) [file pmed.1003798.s013.docx]

# S4 Table: Effect of combinations of interventions on satisfaction with how the results were shared

|  | **Very unsatisfied** | **Quite unsatisfied** | **Neither satisfied nor unsatisfied** | **Quite satisfied** | **Very satisfied** | **Unadjusted OR^[[1]](#footnote-1)^ (vs basic webpage alone)**  **(95% CI)**  **p-value** | **Adjusted OR^[[2]](#footnote-2)^ (vs basic webpage alone)**  **(95% CI)**  **p-value** |
| --- | --- | --- | --- | --- | --- | --- | --- |
| Basic Webpage alone | 2 (13) | 4 (25) | 5 (31) | 2 (13) | 3 (19) | - | - |
| Basic Webpage & Mailed Printed Summary | 2 (13) | 1 (7) | 0 (0) | 4 (27) | 8 (53) | **5.66 (1.47 to 21.81) p=0.012** | **5.34 (1.38 to 20.63) p=0.015** |
| Basic Webpage & Email List Invitation | 1 (6) | 1 (6) | 4 (25) | 7 (44) | 3 (19) | 1.77 (0.53 to 5.91) p=0.352 | 1.65 (0.46 to 5.90) p=0.439 |
| Basic webpage & Mailed Printed Summary & Email List Invitation | 1 (5) | 2 (9) | 2 (9) | 3 (14) | 14 (64) | **7.71 (2.21 to 26.89)**  **p=0.001** | **7.91 (2.22 to 28.20) p=0.001** |
| Enhanced Webpage alone | 2 (13) | 1 (6) | 2 (13) | 4 (25) | 7 (44) | 2.94 (0.81 to 10.62) p=0.101 | 2.85 (0.78 to 10.37) p=0.112 |
| Enhanced Webpage & Mailed Printed Summary | 2 (11) | 2 (11) | 1 (5) | 3 (16) | 11 (58) | **5.12 (1.39 to 8.93) p=0.014** | **5.05 (1.36 to 18.75) p=0.016** |
| Enhanced Webpage & Email List Invitation | 1 (6) | 1 (6) | 1 (6) | 10 (63) | 3 (19) | 2.67 (0.80 to 8.93) p=0.111 | 3.10 (0.90 to 10.66) p=0.072 |
| Enhanced Webpage & Mailed Printed Summary & Email List Invitation | 1 (4) | 0 (0) | 2 (8) | 7 (28) | 15 (60) | **7.21 (2.20 to 23.69) p=0.001** | **7.09 (2.16 to 23.27) p=0.001** |

1. Adjusted for strata, randomisation phase (early vs late) and clustering [↑](#footnote-ref-1)
2. Adjusted for age, education level and internet use as well as strata, randomisation phase (early vs late) and clustering [↑](#footnote-ref-2)
