# Supplementary material for: Testing approaches to sharing trial results with participants: The Show RESPECT cluster randomised, factorial, mixed methods trial
Source: PLoS Med. 2021 Oct 4;18(10):e1003798. doi: 10.1371/journal.pmed.1003798 (PMC8523080; doi:10.1371/journal.pmed.1003798)
Supplement: S5 Table — (DOCX) [file pmed.1003798.s014.docx]

# S5 Table: Qualitative data categories

| **Category** | **Description** |
| --- | --- |
| Choose source | Information about how trial participants go about choosing a source of health information to use. It encompasses issues around credibility and utility. Credibility is the perceived trustworthiness, accuracy, reliability and lack of bias of information. Utility is the perceived usefulness, relevance, timeliness and ease of use. |
|  |  |
|  |  |
|  |  |
|  |  |
| Communication medium | A communication medium is the channel through which information is communicated (eg. the internet). This category contains information about patients views on different communication mediums, including their preferred communication medium for receiving trial results; issues around the accessibility of different communication mediums; whether different mediums are needed for different circumstances; offering choice; and reflections on how the resource limitations of the NHS affect patients’ preferences around communication mediums for trial results. |
|  |  |
|  |  |
|  |  |
|  |  |
|  |  |
| Communication Process | Information about the stages of sharing/receiving trial results; whether an opt-in or opt-out approach should be used, and if opt-in, when patients should be approached; patients expectations around whether they would receive results; whether there is a need for personalisation; the role of the clinical trials unit in the communication process; and patients views around what the process should be for the longer-term results for ICON8. |
|  |  |
|  |  |
|  |  |
|  |  |
|  |  |
|  |  |
| Feelings and thoughts on receiving information | Describes patients’ feelings and thoughts on receiving the Patient Update Information Sheet; printed summary and webpage; and reflections on receiving emotionally challenging results. |
|  |  |
|  |  |
|  |  |
| Information non-use | Describes the reasons why patients decided not to seek further information or use the information products that they were offered. |
| Information product | Contains patients’ views on the information products used within Show RESPECT, including the Patient Update Information Sheet; Mailed Printed Summary; Basic Webpage; Enhanced Webpage; and Email. This includes comments on the content of these information products, and their preferences among the Show RESPECT interventions. |
| Information | Patients’ views on the information contained within the Show RESPECT information products, including whether it was clear; whether it covered what they wanted to know; the language used; the level of information; the framing of the message; the length of the information; and feedback on specific items of information (eg. information on side-effects) |
| Other factors | Patients’ views on other factors that may influence how results should be shared, including patients’ health at the time they receive the results; the design of the trial (whether it is placebo controlled or not); and what the results show. |
|  |  |
|  |  |
| Outcomes | This describes the outcomes that receiving the trial results had on participants, including comprehension, emotional response, interest in future results, questions, patients’ interpretation of the results and their impact, and information users becoming information providers, sharing the information they have received with others, including family, friends and other patients. |
|  |  |
|  |  |
|  |  |
|  |  |
|  |  |
| Sharing results with others  Information User’s context  Information User’s experience receiving the results  Information User’s inhibiting factors | Patients’ views on whether and how trial teams should go about sharing trial results with others, including family members of trial participants, GPs of trial participants, and other patients.  The environmental and personal context of the Information User (trial participant), including their professional background, access to support, their health, their cancer experience, their use of health information generally and about cancer specifically, their relationship with their GP, and fundraising activities they carry out.  Description of participants’ experience of receiving (or not receiving) the results, including how they found out the results; challenges they faced, such as not being told how to access it; site staff prompting patients to access results; patients seeking out the results for themselves; and family members acting as gatekeepers for the information.  Factors inhibiting participants from seeking information, such as not wanting to bother health professionals, and not feeling able to cope with too much information. |
|  |  |
|  |  |
|  |  |
|  |  |
|  |  |
|  |  |
|  |  |
|  |  |
|  |  |
|  |  |
|  |  |
|  |  |
|  |  |
|  |  |
|  |  |
|  |  |
|  |  |
|  |  |
| Information User’s motivating factors | Factors motivating participants to seek information about the trial results, including desire for closure and wanting to know if the trial was any use. |
|  |  |
|  |  |
|  |  |
| Information User’s needs, wants, goals | Things that trial participants need or want, and their goals. This includes what they hope to achieve through their trial participation and their reasons for joining the ICON8 trial; support needs; desire for health/cancer information generally, and information about the ICON8 trial specifically, including feedback as the trial progresses; not wanting to think about cancer; and wanting to protect their family from worry. |
|  |  |
|  |  |
|  |  |
|  |  |
|  |  |
|  |  |
|  |  |
| Information User’s perceptions | Includes trial participants’ perceptions of what the benefits of trial participation are; the importance of research generally; the role of support groups; the relevance of the results to themselves; their own ability to understand health information; the importance of the ICON8 research question; how much site staff know about the trial results; and whether the ICON8 results were communicated properly. |
|  |  |
|  |  |
|  |  |
|  |  |
|  |  |
|  |  |
|  |  |
| Information User’s views | Trial participants’ views of the benefits of sharing results, including putting their individual experience in context and showing that participants are valued. |
| Using communication | Descriptions of how trial participants’ used the information products, including reading the results; processing them; keeping the information for future reference; discussing the results with others, and relating the overall ICON8 results to their trial experience. Also includes difficulties taking the results in. |
