# Supplementary material for: Testing approaches to sharing trial results with participants: The Show RESPECT cluster randomised, factorial, mixed methods trial
Source: PLoS Med. 2021 Oct 4;18(10):e1003798. doi: 10.1371/journal.pmed.1003798 (PMC8523080; doi:10.1371/journal.pmed.1003798)
Supplement: S6 Table — (DOCX) [file pmed.1003798.s015.docx]

**S6 Table: Proportion of participants who wanted to know the results reporting finding them out, by randomisation and subgroup**

|  | **Webpage** | | **Mailed Printed Summary** | | | **Email List Invitation** | | **Overall** |
| --- | --- | --- | --- | --- | --- | --- | --- | --- |
|  | **Basic Webpage**  n/N (%) | **Enhanced Webpage**  n/N (%) | **No Printed Summary**  n/N (%) | **Printed Summary**  n/N (%) | **No Invitation**  n/N (%) | | **Invitation**  n/N (%) |  |
| Overall | 71/80 (89) | 74/84 (88) | 67/81 (83) | 78/83 (94) | 65/74 (88) | | 80/90 (89) | 145/164 (88) |
| **Site size**^^[[1]](#footnote-1)^^ | | | | | | | | |
| Small | 13/14 (93) | 21/24 (88) | 16/20 (80) | 18/18 (100) | 19/21 (90) | | 15/17 (88) | 34/38 (89) |
| Medium | 22/26 (85) | 15/17 (88) | 15/20 (75) | 22/23 (96) | 18/22 (82) | | 19/21 (90) | 37/43 (86) |
| Large | 36/40 (90) | 38/43 (88) | 36/41 (88) | 38/42 (90) | 28/31 (90) | | 46/52 (88) | 74/83 (89) |
| **Age** | | | | | | | | |
| ≤70 years | 42/45 (93) | 43/48 (90) | 41/48 (85) | 44/45 (98) | 33/38 (87) | | 52/55 (95) | 85/93 (91) |
| >70 years | 29/35 (83) | 31/36 (86) | 26/33 (79) | 34/38 (89) | 32/36 (89) | | 28/35 (80) | 60/71 (85) |
| **ICON8 arm** | | | | | | | | |
| Standard treatment | 16/20 (80) | 21/75 (75) | 16/24 (67) | 21/24 (88) | 17/20 (85) | | 20/28 (71) | 37/48 (77) |
| Dose fractionated paclitaxel | 29/31 (94) | 24/26 (92) | 28/30 (93) | 25/27 (93) | 24/27 (89) | | 29/30 (97) | 53/57 (93) |
| Dose fractionated carboplatin & paclitaxel | 26/29 (90) | 29/30 (97) | 23/27 (85) | 32/32 (100) | 24/27 (89) | | 31/32 (97) | 55/59 (93) |
| **Education level** | | | | | | | | |
| Up to A level | 52/59 (88) | 56/66 (85) | 48/60 (80) | 60/65 (92) | 53/62 (85) | | 55/63 (87) | 108/125 (86) |
| Degree or above | 19/21 (90) | 18/18 (100) | 19/21 (90) | 18/18 (100) | 12/12 (100) | | 25/27 (93) | 37/39 (95) |
| **Use of internet/email** | | | | | | | | |
| Less than daily | 24/28 (86) | 32/37 (86) | 27/32 (84) | 29/33 (88) | 24/29 (83) | | 32/36 (89) | 56/65 (86) |
| Daily | 46/51 (90) | 42/47 (89) | 39/48 (81) | 49/50 (98) | 41/45 (91) | | 47/53 (89) | 88/98 (90) |

1. Small sites had 5 or fewer ICON8 patients, Medium sites 6-12 ICON8 patients, and large sites 13 or more ICON8 patients alive at the time of the site agreeing to be part of Show RESPECT. [↑](#footnote-ref-1)
