# Supplementary material for: Testing approaches to sharing trial results with participants: The Show RESPECT cluster randomised, factorial, mixed methods trial
Source: PLoS Med. 2021 Oct 4;18(10):e1003798. doi: 10.1371/journal.pmed.1003798 (PMC8523080; doi:10.1371/journal.pmed.1003798)
Supplement: S7 Table — (DOCX) [file pmed.1003798.s016.docx]

# S7 Table: Qualitative Feedback on the interventions within Show RESPECT

|  | **Basic webpage** | **Enhanced webpage** | **Mailed Printed summary** | **Email** |
| --- | --- | --- | --- | --- |
| **General views** | Many of the comments about the basic webpage were positive, with patients describing it  as good and informative.  *"Clear, readily available information, presented in a factual manner - easy to absorb the*  *information"* CSQ06 | Many of the comments about the enhanced webpage overall were positive, with patients describing it as clear and easy to understand. | Patients were generally very positive about the printed summary.  *"But that’s really good, that summary, I like it, that is a really well put together piece of information."* BLI01 | In principle, most of the women I interviewed said they would be fine with receiving results via email. |
| **Content** | For some patients, the basic webpage contained everything that they expected to be in a summary of the results. However, some patients thought there were some items of content that they would have liked to be included in the basic webpage, including thanking patients and information on where to go for further support.  The basic webpage started with the full, scientific, study name, registration numbers and who sponsored the study. There were mixed opinions about whether this information was necessary for patients.  *"I think it’s important to me who sponsored the study, the general information about the study, the*  *amount of women that were included in the study on the different groups. I think it’s quite necessary."* BLI01  *“I’m not quite sure of the necessity to put all, I don’t know what it means even, under number 1, Study name.”* DLI01  One patient commented positively on the links to further information in section 10.  Another patient thought the basic webpage was too wordy. | The enhanced webpage starts by thanking participants for taking part in the trial, which led one patient to describe the enhanced webpage as more personal and more speaking to the patient than the basic webpage. The level and amount of content was about right for some patients.  *"It's just got enough there that people can understand, because I think you lose people if it's too… They need to know enough, but I think sometimes you can go in too deep and I think it's probably*  *just at the right level."* GMI02  The extra components of the enhanced webpage (diagrams, video, FAQ section and links to further information and support) were generally liked by most participants, with many thinking they would be useful for other patients, if not themselves. | There were mixed feelings around the length of the printed summary, with some describing the printed summary as short, and a good length, whereas others thought it was too wordy and would benefit from being reduced in length.  *"It wasn’t too long. I thought it was about the right length of report giving information."* CLI01  *"Well I personally think if it had been half as long, it would have been more interesting. I think all*  *you’ve got to do is cut the words down."* GMI01 | The content of the email was the same as the enhanced webpage (minus the video), so see the comments on content for the enhanced webpage. |
| **Language** | There were mixed views on the language used in the Basic Webpage, which started with the full scientific title of the ICON8 trial, which does use medical jargon, before moving on to plainer English.  *"This one is more clinical I guess and I’m not used to reading clinical trials particularly."* CLI01  *"It was in language that worked for somebody who wasn't medically trained."* CSI01  The tone of the writing was also noticed, with the basic webpage being described as being cold  and less conversational than the enhanced webpage. However, this did not hinder understanding. | The language used in the Enhanced webpage, printed summary and email was the same. Many patients described the language as clear and easy to read, not using too much jargon.  *"I think the language is good because it wasn’t technical language at all. I thought it was quite easy to understand."* DMI01  *"It was quite short sentences, not too much jargon."* FLI01 | | |
| **Layout & structure** | Patients described the layout of the basic webpage as clear, and talked about it being easy to navigate because of the use of headings. They appreciated that the text was split up into small chunks rather than long paragraphs. Some found the single column layout easier to  follow than the enhanced webpage, which used two columns.  There were mixed views on the font size, which was 14px, however, one patient who had viewed the enhanced webpage first, which uses larger type, asked if the basic webpage was meant for healthcare professionals because of the smaller type.  Some patients found the layout dull and would have preferred to have information conveyed in a more graphical way.  For some patients, the structure of the information was logical. However, some found it confusing, and gave up before reaching the results. | Most patients liked the look of the enhanced webpage, describing it as user-friendly, clear and pleasing to look at. The diagrams added colour to the webpage that was missing from the basic webpage, which one patient said made the enhanced webpage more appealing to look at.  *"Well immediately it looks more user-friendly... it’s clear, it’s plain"* DLI01  However, some patients found the two column layout of the enhanced webpage distracting, or even messy and anxiety provoking.  The enhanced webpage contained 'quick links' at the top of the page (in the right hand column), so patients could navigate straight to any section they were interested in. The feature was appreciated by some. | Patients appreciated the clear headings, which they said made it easy to find the information they were most interested in. Several patients also commented that they liked the text being broken up into small chunks. The use of colour in the printed summary was appreciated. Several patients noted approvingly the large font size used for the printed summary.  *"I quite like the fact that it is slightly a larger font size, and I know that my husband would find it easier to read something like this than something with a smaller font." CSI01* | Participants generally said they liked the layout of the email. |
| **Suggested adaptations** | Some patients would have liked a webpage that contained some of the extra features of the enhanced webpage (video, FAQ section and links to support), while maintaining the single column layout of the basic webpage.  *"You could put the support thing at the bottom, maybe put the video in it. And I think where you can ask a question, I think that could be quite good, and see what questions other people have*  *asked as well. You could bung that on the bottom, so that you’re not cluttering up the sides with*  *more things."* FLI01 | Apart from the few patients who disliked the two-column layout, most patients had no suggestions for how to improve the enhanced webpage. | One patient commented that she would prefer it if the printed summary had been broken up into more pages, with less information on each page, and use of pictures to make the text more attractive. Another suggested change was to emphasise the key messages and thank you more visually. Other changes suggested include reducing the length of the printed summary,  emphasising key messages more visually, and using more bullet points. | The only suggestion for improvement for the email was that women should be able to opt-in to the email list when they joined the study. |
| **Preferences** | A few patients preferred the basic webpage to the enhanced webpage. This was because they found the simpler layout of the basic webpage easier to navigate. | Most patients preferred the enhanced webpage to the basic webpage. They often gave multiple reasons for preferring the enhanced webpage to the basic  webpage. For some, this preference was, at least in part, due to the content items that the basic webpage lacked (FAQ section, diagrams, video, thanks). For some patients, the visual  layout or navigability of the webpage was a reason for preferring it. Readability was  another reason given for preferring it. | Many patients said they would prefer to receive results via the printed summary sent by post, rather than the webpages or email. For some that was the convenience of not having to access the webpage for themselves (particularly for those who were less confident with  computers), and for others it was about having a physical copy they could read and file.  While many patients preferred to have a hard copy of the results, some who received the printed summary would have preferred to have looked up the results online, either because they felt it would be easier to understand online, or because it would save the NHS the costs of posting and printing. | The way the interventions were delivered in Show RESPECT, women who wanted to receive results by email had to visit a url to sign up to the mailing list. For women who wanted the results immediately, it was quicker to access the website. And for those who were less concerned about speed, the printed summary was an easier option, as the opt-out approach meant they did not need to take action to receive it. |
