# Supplementary material for: Testing approaches to sharing trial results with participants: The Show RESPECT cluster randomised, factorial, mixed methods trial
Source: PLoS Med. 2021 Oct 4;18(10):e1003798. doi: 10.1371/journal.pmed.1003798 (PMC8523080; doi:10.1371/journal.pmed.1003798)
Supplement: S10 Table — (DOCX) [file pmed.1003798.s019.docx]

# S10 Table: List of staff involved at Show RESPECT sites

NB. Staff are listed in alphabetical order (by first name) within each site.

* indicates Principal Investigator for site.

| **Site** | **Name** |
| --- | --- |
| Addenbrookes | Cara Mason |
| Addenbrookes | Christine Parkinson* |
| Addenbrookes | Ellie Couch |
| Addenbrookes | Ilene Cannon |
| Addenbrookes | Joe Biggins |
| Addenbrookes | Scott Cheetham |
| Airedale | Alison Shaw |
| Airedale | Dan Lee* |
| Airedale | Helen Hothersall |
| Airedale | Lucy Sootheran |
| Barts | Alia Mahboob |
| Barts | Ana-Marie Pena-Remorin |
| Barts | Jennifer Moore |
| Barts | Rowan Miller * |
| Barts | Sadiyah Robbani |
| Bedford | Carina Galpin |
| Bedford | Jennier Valentine |
| Bedford | Laura Kelly |
| Bedford | Sarah Smith* |
| Broomfield | Christian Barnett |
| Broomfield | Helena Nam* |
| Broomfield | Jennier Child |
| Broomfield | Lauren Perkins |
| Broomfield | Tracey Camburn |
| Broomfield | Yvonne Lester |
| Castle Hill | Caroline Abernathy |
| Castle Hill | Darren Camp |
| Castle Hill | Georgios Bozas* |
| Castle Hill | Louise Hunn |
| Cheltenham | Audrey Cook* |
| Cheltenham | Jill Chittock |
| Cheltenham | Julia Hall |
| Cheltenham | Sarah Beazer |
| Christie | Gordon Jayson* |
| Christie | Kate Prideaux |
| Christie | Linzi Davies |
| Clatterbridge | Danielle Shaw |
| Clatterbridge | Hannah Doughty |
| Clatterbridge | Nicholas Garbutt |
| Clatterbridge | Rosemary Lord* |
| Dorset County | Andrew Rees |
| Dorset County | Karen Hogben |
| Dorset County | Maxine Flubacher* |
| Dorset County | Zoe Sheppard |
| George Eliot | Judith Lake* |
| George Eliot | Pritpal Klear |
| George Eliot | Shelley Grant |
| Great Western | Jean Kordula |
| Great Western | Omar Khan* |
| Great Western | Sarah Louise Matthews |
| Hammersmith | Chynna Pascal |
| Hammersmith | Emily Pickford |
| Hammersmith | Hani Gabra* |
| Hammersmith | Maria Martinez |
| Hammersmith | Ruth Nicholson |
| Hinchingbrooke | Li Tee Tan* |
| Hinchingbrooke | Rebecca Lam |
| Huddersfield | Deivasikamani Ramanujam* |
| Huddersfield | Lesley Thomis |
| Huddersfield | Lisa Gledhill |
| Huddersfield | Selina Shaw |
| Huddersfield | Wendy Cook |
| Ipswich | Frances Farnworth |
| Ipswich | Liz Sherwin* |
| Ipswich | Paul Ridley |
| Ipswich | Richard Smith |
| Ipswich | Sarah Batholomew |
| Ipswich | Susan Upson |
| Maidstone | Amie Thomas |
| Maidstone | Gavin Fossey |
| Maidstone | Jeff Summers* |
| Mount Vernon/Lister | Ignacio Vazquez* |
| Mount Vernon/Lister | Lwam Habteab |
| Mount Vernon/Lister | Robyn Dacres-Sam |
| Musgrove Park | Ali Chedham |
| Musgrove Park | Angela Locke |
| Musgrove Park | Clare Barlow* |
| Norfolk & Norwich University Hospital | Adele Cooper |
| Norfolk & Norwich University Hospital | Daniel Epurescu* |
| Norfolk & Norwich University Hospital | Denise Archer |
| Norfolk & Norwich University Hospital | Julie Cook |
| Norfolk & Norwich University Hospital | Julie Mercer |
| Norfolk & Norwich University Hospital | Michael Sheridan |
| Norfolk & Norwich University Hospital | Susan Halliwell-Bass |
| North Devon District Hospital | Becky Holbrook |
| North Devon District Hospital | Jennifer Morrison |
| North Devon District Hospital | Kate Scatchard* |
| North Devon District Hospital | Lynne Van Koutrik |
| North Devon District Hospital | Mark Bryce |
| North Devon District Hospital | Martin Howard |
| Northampton | Andrea Kempa |
| Northampton | Michelle Spinks |
| Northampton | Roshan Agarwal* |
| Nottingham University Hospital | Anjana Anand* |
| Nottingham University Hospital | Jo Hobbs |
| Nottingham University Hospital | Johnathan Ho |
| Nottingham University Hospital | Kerri Jenkins |
| Nottingham University Hospital | Louisa Jones |
| Peterborough City Hospital | Elisa Barter |
| Peterborough City Hospital | Kerrie Cavanagh |
| Peterborough City Hospital | Marcia Mulligan |
| Peterborough City Hospital | Sarah Ayers* |
| Peterborough City Hospital | Tessa Stoby |
| QEQM | Joanne Williams |
| QEQM | Justin Waters* |
| QEQM | Miranda Foad |
| QEQM | Sarah Lines |
| QEQM | Susan Kelly |
| Queen's Romford | Helen Mackenzie |
| Queen's Romford | Kate Donaldson |
| Queen's Romford | Mary Quigley* |
| Queen's Romford | Tina Mills-Baldock |
| Royal Berksire Hospital | Chelsea Gallagher |
| Royal Berksire Hospital | Emma Vowell |
| Royal Berksire Hospital | Jessica Mckean |
| Royal Berksire Hospital | Madhumita Bhattacharyya* |
| Royal Berksire Hospital | Maxine Gauntlett |
| Royal Cornwall | Abigail Weeks |
| Royal Cornwall | Alyson Andrew |
| Royal Cornwall | Angela Loweth |
| Royal Cornwall | Kirsty Maclean* |
| Royal Cornwall | Laura Royle |
| Royal Cornwall | Micheala Ayers |
| Royal Derby Hospital | Mojca Persic* |
| Royal Derby Hospital | Nicole McKee |
| Royal Devon and Exeter | Alison Roantree |
| Royal Devon and Exeter | Kate Scarchard* |
| Royal Devon and Exeter | Kizzy Baines |
| Royal Devon and Exeter | Suzanne Tasker |
| Royal Lancaster Infirmary | Aileen Menzies |
| Royal Lancaster Infirmary | Claire Bartlett |
| Royal Lancaster Infirmary | Gail Wiley |
| Royal Lancaster Infirmary | Margaret Cooper |
| Royal Lancaster Infirmary | Nicola Mackenzie |
| Royal Lancaster Infirmary | Sarah Moon* |
| Royal Marsden Hospitals | Alexandra Habich |
| Royal Marsden Hospitals | Kylie Fitch |
| Royal Marsden Hospitals | Laura Pope |
| Royal Marsden Hospitals | Melody Ncube |
| Royal Marsden Hospitals | Monica Hamill |
| Royal Marsden Hospitals | Susana Banerjee* |
| Royal Shrewsbury | Alison Tilley |
| Royal Shrewsbury | Angela Loughlin |
| Royal Shrewsbury | Rebecca Wilcox |
| Royal Shrewsbury | Sally Potts* |
| Royal United Hospital | Denise Preece |
| Royal United Hospital | Kelly Spencer |
| Royal United Hospital | Rebecca Bowen* |
| Royal United Hospital | Samantha Curtis |
| Royal United Hospital | Thomas Tylee |
| Southend | Bernanrd Hadebe |
| Southend | Eugene Mphansi |
| Southend | Helena Nam* |
| Southend | Tracey Davies |
| St George's | Antonio Pesino |
| St George's | Claire Gilmartin |
| St George's | Fiona Lofts* |
| St George's | Jesusa Toledo |
| St George's | Sam Hollingworth |
| St James (Leeds) | Gaushiyanu Saiyad |
| St James (Leeds) | Gwendolyn Saalmink |
| St James (Leeds) | Helen Payne |
| St James (Leeds) | Jade McCann |
| St James (Leeds) | Jane Hook* |
| St James (Leeds) | Jude Clarke |
| Torbay | Ingrid Koehler |
| Torbay | Ken Almedilla |
| Torbay | Nangi Lo* |
| Torbay | Sarah Youlden |
| Torbay | Shelley Chamberlain |
| UHCW | Chris Poole* |
| UHCW | Kate Herbert |
| UHCW | Kay Sanders |
| UHCW | Pauline Kelly |
| Velindre | Emily Rumney |
| Velindre | Kathy Bishop* |
| Warwick Hospital | Denise Hrouda* |
| Warwick Hospital | Frances Walsh |
| Warwick Hospital | Jo Williams |
| Warwick Hospital | Kerrie Webb |
| Weston Park | Alison Redfearn |
| Weston Park | Catharine Spalton |
| Weston Park | Emma Griffiths |
| Weston Park | Katharine Behennah |
| Weston Park | Luke Barron |
| Weston Park | Simon Pledge* |
| York | Angela Darby* |
| York | Cancer Research Team |
| York | Claire Brookes |
| York | Richard Furnival |
| York | Sally Gilroy |
| York | Shamila Saleem |

* = Principal Investigator
