# Supplementary material for: Testing approaches to sharing trial results with participants: The Show RESPECT cluster randomised, factorial, mixed methods trial
Source: PLoS Med. 2021 Oct 4;18(10):e1003798. doi: 10.1371/journal.pmed.1003798 (PMC8523080; doi:10.1371/journal.pmed.1003798)
Supplement: Attachment — Submitted filename: PLOS Medicine Resubmission Covering letter v1 020921.pdf [file pmed.1003798.s021.pdf]

PLOS Medicine
1265 Battery Street, Suite 200
San Francisco, CA 94111
United States

Thursday, 02 September 2021

Dear Dr Davidson,

We are delighted that PLOS Medicine has decided to accept our manuscript, subject to us dealing with the remaining editorial and production issues. We thank the reviewers and editors who took the time to re-review the manuscript.

We have made the changes requested in your email:

- Legends have been added to Figures 1, 2 and S8 Fig – do let us know if we have not interpreted what you asked for correctly.
- We have trimmed the author summary as requested
- We have removed the ‘Data availability’ and ‘Funding source’ sections from the manuscript main body
- We have removed the 12 month delay period for data availability, and have updated the information on the submission system
- We have removed the ‘Contributor and guarantor information’, ‘Copyright’ and ‘Competing interests’ sections from the main body of our manuscript.
- We have removed the funding information from the abstract.

I will be on leave from 3rd-19th September, and will not be able to respond to emails, so apologies in advance for any delay in replying to you.

Do please let me know if you require any further changes or information from me.

Yours sincerely

Annabelle South

Annabelle South
Policy, Communications & Research Impact Coordinator

For the Show RESPECT collaborators

MRC Clinical Trials Unit at UCL
Institute of Clinical Trials & Methodology

Direct line: +44 (0)20 7670 4827
Email: a.south@ucl.ac.uk
