# Supplementary material for: Testing approaches to sharing trial results with participants: The Show RESPECT cluster randomised, factorial, mixed methods trial
Source: PLoS Med. 2021 Oct 4;18(10):e1003798. doi: 10.1371/journal.pmed.1003798 (PMC8523080; doi:10.1371/journal.pmed.1003798)
Supplement: Attachment — Submitted filename: Responses to Reviewers comments v1.docx [file pmed.1003798.s020.docx]

# Responses to Editors’ and Reviewers’ comments

|  | **Editors’ / Reviewer’s comment** | **Authors’ response** | **Location of change (‘All mark up’ view)** |
| --- | --- | --- | --- |
|  | **Editors** |  |  |
| E.1 | The feeling amongst the editorial team was that the manuscript needs to be better organized and condensed before publication can be considered. Please aim to streamline the manuscript where possible, taking into account the comments from peer reviewers. One suggestion may be to separate out the presentation of the quantitative and qualitative aspects and relocate some of the qualitative work to the supplementary materials section. | We thank the editors and reviewers for their comments. We recognise that the manuscript requires streamlining. We have done this through moving much of the qualitative findings relating to secondary outcomes to the supplementary materials. We did, however, maintain the integration for the primary outcome and findings related to accessibility, as we believe these are important as primary outcome data and for understanding our headline result.  The study was designed as a mixed methods study, and we consider both to have equal weight in shedding light on the topic (rather than viewing the qualitative component subsidiary to the RCT). We feel it is important to integrate the findings from both quantitative and qualitative components, consistent with the study design. Lack of integration of qualitative and quantitative findings in many mixed methods studies in healthcare has been criticised as a major limitation <https://doi.org/10.1136/bmj.b3496> which we want to avoid. The structure of our results section (with both quantitative and qualitative results on an outcome presented before moving on to the next outcome) is a function of the method we chose for triangulating the different types of data (‘following the thread’). Maintaining an integrated presentation of the quantitative and qualitative results for the primary outcome (while segregating to supplemental data additional secondary outcomes) is important in mixed methods research and, we hope, provides a compromise that allows the streamlining of the manuscript that the editors’ and reviewer’s suggestion was meant to achieve.  We hope this approach addresses the editors concerns while maintaining the strength of our approach | Page 9 Lines 150-152; 155-158.  Page 10 Lines 159-160.  Page 12 lines 230-231; Page 13 lines 232-247.  Page 25 lines 529-531  Page 28 lines 552-556.  Page 38, lines 627-635.  Page 39, lines 636-659.  Page 40, lines 660-671.  Page 41, lines 683-695, 702-708.  Page 42 lines 709-726.  Page 44, lines 777-782.  Page 45, lines 783-805.  Page 46, lines 806-825, 829-830.  Page 47, lines 830-844.  Page 48, lines 854-857.  Page 51, lines 889-890, 892-894. |
| E.2 | In your data availability statement, please include details of where the CTU’s ‘Data Sharing Policy’ can be located | We have made the requested change, adding the URL to the Data Availability section | Page 59, lines 1038-1039 |
| E.3 | Please revise your title according to PLOS Medicine's style. Your title must be nondeclarative and not a question. It should begin with main concept if possible. I would suggest ‘Testing approaches to sharing trial results with participants: The RESPECT cluster randomised, factorial, mixed methods trial’. | We have revised the title as requested, to read: ‘Testing approaches to sharing trial results with participants: The Show RESPECT cluster randomised, factorial, mixed methods trial’ | Page 1, lines 1-4 |
| E.4 | Please structure your abstract using the PLOS Medicine headings (Background, Methods and Findings, Conclusions).  Abstract Background: Provide the context of why the study is important. The final sentence should clearly state the study question.  Abstract Methods and Findings:  * Please ensure that all numbers presented in the abstract are present and identical to numbers presented in the main manuscript text (e.g. 72% is included in the abstract but I could not locate this in the main text).  * Please include the years during which the study took place and length of follow up.  * In the last sentence of the Abstract Methods and Findings section, please describe the main limitation(s) of the study's methodology." | We have restructured the abstract using the PLOS Medicine headings.  We have checked the numbers in the abstract are identical to those in the text and removed the 72%.  We have added the years during which the study took place, the length of follow-up and the main limitation:  “The study was carried out from 2018-2019.”  “Follow-up questionnaires were sent one month after patients had been offered the interventions.”  “The main limitation of our study is the 65% response rate to the questionnaire.” | Page 4, lines 54-76.  Page 5, lines 77-100.  Page 6, lines 101-110. |
| E.5 | At this stage, we ask that you include a short, non-technical Author Summary of your research to make findings accessible to a wide audience that includes both scientists and non-scientists. The Author Summary should immediately follow the Abstract in your revised manuscript (you currently include something similar but please update to match the Author Summary format). This text is subject to editorial change and should be distinct from the scientific abstract. | We have added an Author Summary as requested. | Page 7, lines 112-137.  Page 8, lines 138-143. |
| E.6 | Please relocate paragraph 1 (lines 406-410) of the Results section into the Methods section. | We have made the requested change. | Page 16, lines 314-317. Page 18, lines 364-365. |
| E.7 | Please specify whether informed consent was written or oral. | Written informed consent was given by participants in the qualitative study – we have added this information. We have also expanded the information on the consent process for the quantitative questionnaire. | Page 18, lines 376-380.  Page 22, line 466 |
| E.8 | Please begin the Discussion with a short, clear summary of the article's findings and ensure the strengths and limitations of the study are clearly described in their own paragraph later in the Discussion. | We have added a short clear summary of the article’s findings (both qualitative and quantitative) to the start of the Discussion. The first paragraph of the discussion then summarises the articles quantitative findings, while the second paragraph presents a short summary of the qualitative findings.  The third paragraph describes the strengths of the study, while the fourth paragraph discusses the limitations. | Page 50, lines 862-868 |
| E.9 | Thanks for providing a CONSORT checklist. Please update the checklist to use section and paragraph numbers, rather than page numbers (as these will change in subsequent revisions). | We have made this change. | S15 Table |
| E.10 | As part of your revision, please complete and submit a copy of the COREQ Guidelines checklist, a document that aims to improve reporting of qualitative studies for purposes of post-publication data analysis and reproducibility | We have completed the COREQ checklist and uploaded it as S16 Table. We have had to expand the qualitative methods section to accommodate all the checklist requirements. | S16 Table |
| E.11 | Your trial appears to have been registered after the participants were randomized. Please explain in the paper why your trial was registered late. | We have added this to the manuscript: “The trial was registered in February 2019, which was after some sites had been randomised, due to human resource constraints.” | Page 18, lines 365-367. |
| E.12 | The sample size listed in the submitted manuscript and the trial registry differ. This discrepancy appears to be due to the manuscript reporting participants offered the intervention as well as those that returned questionnaires. Please ensure it is made clear throughout the manuscript how many participants were included in your analyses. | We have clarified how many participants were included in our analyses throughout the manuscript. | Page 24, lines 521-522.  Tables 4, 5, 6, 7 |
| E.13 | Please include the study protocol document and analysis plan, with any amendments, as Supporting Information to be published with the manuscript if accepted. | We have included these as S17 Appendix and S18 Appendix. | S17 Appendix  S18 Appendix |
|  | **Reviewer 1** |  |  |
| 1.1 | Thank you for the opportunity to review this manuscript. The authors tackle an important question regarding offering research results to participants. As ICON8 was a high-impact, and large trial, I think the experience in this manuscript will be useful to investigators considering how to communicate results to participants in planned trials. | We thank the reviewer for their comments, and are glad that they think our results will be useful to investigators in other trials. | n/a |
| 1.2 | I do not think that the manuscript is suitable for publication in its current form. There is way too much text here; over 100 pages were included in the pdf for my review: the manuscript itself is ~7500 words and the tables are large and confusing to navigate. The authors' important results will not be communicated effectively if the reader (even if familiar with gynecologic cancers and communication of research results) cannot make it through the manuscript. It may be that the qualitative component should be presented in a separate submission; in my opinion the quantitative component is more important and likely to influence research practice. Suggest a target of ~3000 words for these data. | We appreciate the reviewer’s point that a manuscript that is too long and confusing to navigate will not effectively communicate results. In the light of the reviewer’s comments, we have trimmed the paper and moved much of the qualitative results to the Supplementary Materials.  We have separated Table 4 into three separate tables, to make it easier to navigate.  However, we must not completely separate out the qualitative findings as this study was designed as a mixed methods study, with equal weight placed on the quantitative and qualitative components, particularly with respect to the primary outcome. We believe presenting both aspects together is a strength of our work and concordant with the original study design. Qualitative and quantitative results from mixed methods studies are often presented separately, meaning the potential value of the mixed methods approach is not fully realised (see <https://trialsjournal.biomedcentral.com/articles/10.1186/1745-6215-15-215>). In this study, the qualitative findings aid in understanding our quantitative results. Through highlighting the experiences and views of our participants, (as well as their categorical responses), we hope to encourage trialists to consider the needs, preferences and hopes of participants around the sharing of trial results; the quantitative data alone does not adequately captures this, and for too long practice around sharing results with participants in trials has been non-existent or poor.  We have moved much of the qualitative data to supplemental data (other than those essential for understanding the quantitative data) and restructured the manuscript. We have shortened the manuscript considerably.  The reviewer’s suggested target word limit, however, is not possible for reporting a mixed methods study while complying with CONSORT and COREQ guidance (see <https://bmcmedresmethodol.biomedcentral.com/articles/10.1186/1471-2288-9-82> for discussion of this issue). | Page 9 Lines 150-152; 155-158.  Page 10 Lines 159-160.  Page 12 lines 230-231; Page 13 lines 232-247.  Page 25 lines 529-531  Page 28 lines 552-556.  Page 38, lines 627-635.  Page 39, lines 636-659.  Page 40, lines 660-671.  Page 41, lines 683-695, 702-708.  Page 42 lines 709-726.  Page 44, lines 777-782.  Page 45, lines 783-805.  Page 46, lines 806-825, 829-830.  Page 47, lines 830-844.  Page 48, lines 854-857.  Page 51, lines 889-890, 892-894.  Tables 4, 5, 6.  S10 Table.  S12 Text.  S13 Text. |
| 1.3 | Suggest considering using "mailed" or "sent via post" instead of "posted." For most of the early part of this paper I thought that "posted" meant "tacked to a wall in a public place" rather than "sent via postal service." | We have made this change throughout. | Page 4, lines 56-57, 75.  Page 5, lines 87, 88, 90, 92  Page 6, line 104  Page 8, line 141  Page 11, lines 203-4  Page 14, lines 266-7, 274  Page 15, lines 285, 288  Table 1  Page 26 lines 529, 532  Table 2  Page 28, line 551  Table 3  Page 30, line 567  Table 5  Page 37, line 592  Page 38, lines 618, 620  Page 40, lines 679-80  Page 43 lines 733, 740, 745  Table 7  Page 50, line 863,  Page 51, line 894  Page 56, line 990  Page 58, line 1026 |
| 1.4 | Description of ICON8 in lines 141-143 is confusing as written, and q 3 week chemo is not always standard of care... this is beside the point of this article and distracting to a reader familiar with the trial. Perhaps better to say RCT of three chemotherapy regimens for up-front treatment of ovarian cancer and leave it at that. | We have made this change. | Page 11, lines 191-193 |
| 1.5 | Table 4 and according locations in the text: what do the odds ratios represent? | We have added text to the methods to clarify how odds ratios from ordinal logistic regression should be interpreted. | Page 20, lines 420-424 |
| 1.6 | The Discussion undersells the impact of making conclusions on hard-copy versus website/email list in a population primary composed of older women. I would want to see a little more specific discussion of which populations might benefit from (cheaper) more technologically-focused methods of results communication, and which might require results sent by post. | We have expanded our discussion of this:  “Four in ten of our respondents reported using the internet or email less than daily, with 15% never using them. Data from the UK Office for National Statistics in 2019 shows that 10% of the UK population are classed as internet non-users, having either never used the internet, or not used it in the last three months [41]. Internet non-users in the UK are more likely to be women, over the age of 65, have a disability, or be economically inactive (particularly those on long-term sick leave) [41]. Households with lower incomes are also less likely to have an internet connection [41]. While the number of internet non-users has been declining in recent years, trialists should be careful about relying on the internet or email to share results with participants if their trial population overlaps with some of the groups most likely to be internet non-users. Failure to take this into account could exclude a significant proportion of participants from accessing results.”  Firmer discussions will follow after others have repeated this study (and variations on it) in other populations. | Page 55, lines 973-996 |
| 1.7 | Would be interesting to read whether the authors believe that there is an ethical obligation to budget for sending out trial results by mail in a similar patient population. | We have added the following text to the discussion:  “If trials are to meet the ethical obligation to offer results to participants, they need to plan and budget for this in a way that is accessible to the trial population. Trials with similar patient populations should budget for Mailed Printed Summaries, or ensure alternative approaches to webpages are easily available and known to for those participants for whom webpages are inaccessible.” | Page 55, lines 988-992 |
|  | **Reviewer 2** |  |  |
| 2.1 | It is great to see a robust SWAT comparing different approaches to the dissemination of results to trial participants. Overall, I found the manuscript very well presented and most of my comments are minor. | We thank the reviewer for their comments. | n/a |
| 2.2 | My only major comment relates to missing data. Participants are lost at two stages: 1. a number of participants who were offered the intervention were not sent the questionnaire and 2. A number who were sent the questionnaire did not return it. I understand that only a random sample of those offered the intervention were included due to logistical constrains; however, this departs from the ITT principle. Out of the 274 participants who were sent a questionnaire, only 180 (66%) returned it. This large amount of missing data creates substantial uncertainty and would probably warrant further sensitivity analyses as well as more recognition in the discussion. | In cluster randomised trials it is not unusual to collect outcome data from only a random sample of the members of clusters, for example where clusters are very large. We do not consider those whom we did not send the questionnaire to be ‘missing data’ (although it does reduce our sample size), nor is this a violation of the ITT principle.  Our 65% response rate is comparable to that seen in similar studies with patient reported outcomes, looking at communicating trial results to participants. For example Mancini 2010 had a 69% response rate among breast cancer trial participants, Partridge 2009 had 73% response rate and Donaldson 2009 had a 68% response rate. While we would have liked a higher response rate, our 65% compares favourably to the 32% response rate in Brealey 2010 and the 58% reported in Dorsey 2008.  We have no basis to assess the plausible range of how non-responders may differ from responders. We do not think sensitivity analysis based on arbitrary ranges would add anything.  We have extended the discussion of the uncertainty caused by our response rate. | Page 51 lines 914-918 |
| 2.3 | Abstract, Results: I am not sure it is necessary to provide a breakdown of returned questionnaire by randomised arm in the abstract itself. | We have included this as it is a requirement of the CONSORT abstract checklist. |  |
| 2.4 | Abstract, Conclusion: can we really say that the study "provides *clear* evidence on how to share results". I find the wording too strong considering some of the potential limitations (e.g. missing data / potential selection biases). I would suggest rewording to "the study *some* evidence" | As this is the first randomised trial to compare different approaches to sharing results with participants, our study provides much stronger evidence than has previously been available to guide trial practice in this area. Perhaps the clarification in response to comment 2.5 will make the reviewer feel more comfortable about the basis for our conclusion that Printed Summaries (when added to webpages) were the best intervention that we tested (it was not that we compared them to no intervention). We have revised the statement to read “This study provides evidence on how to share results with other similar trial populations.” | Page 6, line 106 |
| 2.5 | I note that the webpage comparison involved an enhanced webpage compared to a basic webpage (active comparator) whereas the printed summary was compared to no summary at all. The authors conclude that the printed summary had the greatest effect on satisfaction; however, I wonder this is due to the choice of comparator. Would a fairer comparison have been webpage vs no-webpage? | The factorial design of the trial means that all participants received the link to a webpage (either basic or enhanced). The effect of the Mailed Printed Summary we report is therefore in the context of a webpage being also available to participants, and indeed we have no evidence to assess the importance of providing a webpage compared to no webpage. (We decided against having a no intervention comparison as we felt this was unethical, given the high level of desire for results reported in the literature).  We have amended the text in light of the referee’s comment to make it clearer that the printed summary was the most important of the three innovations that we tested, in terms of satisfaction, but we do not know whether it is sufficient to replace the provision of a webpage alongside it. | Page 14, lines 276-277 |
| 2.6 | I am surprised to see that the detectable differences are almost the same with an ICC of 0.01 or 0.05 (see sample size calculation). According to my calculations, with an average cluster size of 4 patients, the inflation factor due to the cluster design goes from 1.03 to 1.15 with an ICC of 0.01 and 0.05, respectively. While this is a moderate increase, I would expect it to have more impact on the detectable difference. Please confirm/clarify. | Our detectable difference calculations were conducted in Stata using the following ‘power twoproportions’ and the resulting differences rounded to the nearest whole percentage. However we realise we omitted to report that a coefficient of variation of 0.6 in cluster size was assumed and used in the calculation, and have now added that detail. | Page 17, lines 347-349 |
| 2.7 | At the start of the analysis section (line 306), the analysis population (mITT) is defined as participants who reported receiving the ICON8 results. Should not the primary analysis population include all who were sent the printed Patient Update Information Sheet (ITT), regardless of whether they actually accessed any result? Or at least all who were sent the questionnaire? If this is a missing data issue (i.e. no satisfaction data was collected in those who did not actually receive the results), some sensitivity analyses may be required (e.g. missing data imputations). | Our primary outcome measure question was carefully worded, after substantial patient and public involvement, to try to make sure we were measuring the concept we were interested in. The wording “How satisfied are you with the **way** you found out the results of ICON8 (rather than the results themselves)?” is not applicable to participants who did not find out the results, and therefore we do not have data on the satisfaction of patients who did not find out the results. These data are not missing.  We say later in the first paragraph of the analysis section, *“To assess the overall effect of the intervention, it is important to interpret the results of the primary analysis alongside results concerning the possible effect of the interventions on whether participants actually found out the ICON8 results.”*  For our data, this interpretation is straightforward. The printed summary resulted in higher satisfaction, and people who wanted to know results were more likely to find them out if they were in the group randomised to the printed summary, while no-one who didn’t want the results found them out. Both measures point in the same direction – the printed summary is superior to no printed summary. | n/a |
| 2.8 | Table 2 shows the baseline characteristics of those who returned the questionnaire. It would be interesting to compare these to participants who did not return the questionnaire to assess the potential for biases. | While we agree this could be interesting, we do not have data on non-responders beyond what is presented in Supplementary Material Table 2. Comparing Supplementary Table 2 with Table 2 shows the age distribution and spread across the ICON8 arms is similar between all eligible ICON8 participants and those that returned the questionnaire. We cannot tell how similar they were on other characteristics. | n/a |
| 2.9 | I cannot find the results of the interaction test between interventions referred to on line 460. Please consider including them. | We have added this information. | Page 30, lines 572-573 |
| 2.10 | Please consider replacing (or augmenting) Supplementary Table 3 with a forest plot to allow an easier comparison of effects across interventions. The forest plot could potentially be included as a figure in the body of the manuscript. I note that none of the interaction tests were significant; however, these are likely underpowered and therefore unlikely to detect existing interactions. | We agree and have augmented the manuscript with a forest plot. Please note that, in response to editorial comments to streamline the manuscript, we have added it to the supplement rather than main body of the manuscript. | S8 Figure |
| 2.11 | Table 6 shows the results of those who reported finding out the results among those who wanted to know. Can you please clarify why the analysis is limited to those who wanted to know instead of all enrolled? Also, the text mentions odds ratios and 95% CI which do not appear in Table 6 which instead shows data by subgroups. I wonder whether data by subgroups should be in the supplement (were these pre-specified?). Please also consider adding the results of the logistic regression to Table 4 together with the other outcomes. | We had limited Table 6 to those who wanted to know as no-one who did not want to know the results reported finding them out.  We have incorporated the overall numbers from Table 6 into Tables 4, 5 and 6 and added the odds ratios and 95% CI (for those who wanted to know) as requested.  We have moved the data from subgroups to the supplementary materials. | Table 4  Table 5  Table 6  S11 Table |
| 2.12 | Please consider shortening the results section as well as rearranging it to have all quantitative results together followed by all qualitative results. | The Editorial team has made a similar comment about length and we have shortened the results section by moving some of the qualitative results to the Supplementary Materials. However, as discussed in our response to comments E.1, we have not separated out the qualitative and quantitative results entirely, as this integration is related to the original study design and method we chose for triangulation. | Page 25 lines 529-531  Page 28 lines 552-556.  Page 38, lines 627-635.  Page 39, lines 636-659.  Page 40, lines 660-671.  Page 41, lines 683-695, 702-708.  Page 42 lines 709-726.  Page 44, lines 777-782.  Page 45, lines 783-805.  Page 46, lines 806-825, 829-830.  Page 47, lines 830-844.  Page 48, lines 854-857. |
| 2.13 | Please consider discussing the impact of missing data as a limitation. | We have added discussion on the impact of this as a limitation. | Page 52, lines 914-918 |
| 2.14 | In the conclusion, I am not sure one can write that Posted Printed Summaries (PPS) were superior to web-based approaches in terms of patient satisfaction and ensuring those who wanted to know the results were able to find them out as there is no direct comparison between posted printed summaries and web-based approaches. In fact, when comparing the raw distribution between enhanced web-based and PPS, the numbers look very similar and one could argue that the two are equivalent and potentially both better than no active intervention. What we can conclude is that PPS showed better satisfaction than no PPS and that there was no significant difference between enhanced web display vs standard display | As noted in response to comment 2.5, our factorial design means we do not have a direct comparison between printed summaries and the web-based approaches, as everyone in the trial received at least a link to one of the webpages. We have amended the text to make it clearer that we have demonstrated that the addition of PPS is beneficial in the context where a webpage is provided. When looking at the raw distribution of satisfaction between the enhanced webpage and printed summary, it is important to remember that half of those in both webpage groups will also have received the printed summary. Supplementary Table 4 includes the comparison of intervention combinations to basic webpage alone and clearly shows that the addition of PPS provides more benefit than replacing the basic webpage with an enhanced version. The reviewer is correct that we found no significant difference in satisfaction between the two webpages. We do not have a ‘no active intervention’ group (we felt this would be unethical, as explained above), so cannot say whether either webpage was superior to not offering the results by that method at all. | n/a |
|  | **Reviewer 3** |  |  |
| 3.1 | This study takes on a very important topic: How do different methods of sharing trial results' influence trial participants' satisfaction with receiving results? The study design and implementation are very complex. Due to practical considerations related to studying participants in an existing randomized controlled trial, the study makes several necessary deviations from an idealized textbook study design and implementation. However, these limitations are outweighed by the novel approach to prospectively randomly assigning trial participants to several types of results sharing activities. These findings do add new information to what is already known, particularly by taking a random assignment approach rather than an observational/single-arm approach. | We thank the reviewer for their comments. | n/a |
| 3.2 | The abstract does not immediately and clearly explain how the interventions were deployed. It would be good to make clear early on that the study is a site-level cluster randomized 2x2x2 such that participants at each site received one of eight possible combinations of communication activities. | We have revised the abstract to clarify this.  “We carried out a cluster randomized, 2 by 2 by 2 factorial, non-blinded, study within a trial, with semi-structured qualitative interviews with some patients (ISRCTN96189403). Each cluster was a UK hospital participating in the ICON8 ovarian cancer trial. Interventions were shared with 384 ICON8 participants who were alive and considered well enough to be contacted, at 43 hospitals (clusters). Hospitals were allocated to share results with participants through one of the 8 intervention combinations based on random permutation within blocks of 8, stratified by number of participants.” | Page 4, lines 60-69. |
| 3.3 | Because no participants actually subscribed to the email list, the "Email List" intervention might be best described as the "Email List Invitation" intervention or similar throughout the entire article and supporting materials. The abstract itself might be improved by making clear that no patients signed up for the email list. | We agree that the reviewer’s term is more accurate. We debated using this term but chose to use the shorter term for the manuscript. However, we have now made the change throughout. We have added that no patients signed up to the email list to the abstract. | Page 4, line 57  Page 5, lines 88, 94.  Page 14, line 269  Table 1  Table 2  Table 3  Page 30, line 570  Table 6  Page 40, lines 680-1  Table 7  Page 51, line 888 |
| 3.4 | Line 322: This assumption is stated without explaining its justification. The reader would likely be helped by providing a brief rationale why "the prior assumption in the Show RESPECT trial design is that there will not be any important interactions between the three interventions." | There was no specific prior evidence to suggest whether or not there would be interactions between the interventions. We were unable to think of a mechanism for potential interactions, so designed the trial based on the assumption that the effect of each intervention (eg. enhanced vs basic webpage) would not be substantially affected by whether or not the participant was allocated to the other interventions. We have added this to the manuscript. | Page 19, lines 400-406 |
| 3.5 | Line 500: It may be useful to clarify why there was so much missing data (40%) on the question about accessing the web pages. The quantity of missing data makes the percentages reported in the Results for this question relatively uninterpretable. | We do not know why this question was so poorly completed in the questionnaires. We agree it makes this data uninterpretable, but we included it for transparency.  Of those who did not complete this question, 60/72 reported having found out the results, 51 of whom were in the printed summary arm, so were likely to have found out the results from the printed summary (we cannot tell if they also used the webpage).  In order to streamline the manuscript as requested, we have moved this paragraph to the supplementary materials, given its limited value for understanding uptake. | Page 38, lines 627-635  S12 Text |
| 3.6 | In the Discussion, it is important to note that the specific results of the ICON8 trial may have influenced participants' satisfaction in general. The content of ICON8's specific results may have interacted with the communication intervention activities to influence participants' perceptions. This is not a major limitation, but should be clearly noted. | It is true that the findings we were communicating may have influenced reported satisfaction in our study. However, this did not come out of the qualitative research as a major driver of satisfaction or dissatisfaction. Interviewees did talk about whether different approaches were needed, had the ICON8 results been different. We have added the following text to the discussion to address the reviewer’s point.  “The ICON8 results that we were communicating (no difference between the trial regimens) may have influenced participants’ reported satisfaction, interacting with the communication interventions. The results of the ICON8 trial did not come out of the qualitative research as a major reason for satisfaction or dissatisfaction with how they were shared, and most participants in the qualitative interviews understood the importance of ‘negative’ trial results.” | Page 56, lines 1001-1006 |
| 3.7 | In the Discussion, it may be useful to elaborate on the decision that "the primary outcome measure was defined only for participants who received the ICON8 trial results, and hence analysis for this outcome was restricted to participants who reported receiving the ICON8 results. For this reason, we describe the primary analysis as following modified intention to treat (mITT)." The main outcome could have been defined with respect to satisfaction with the overall approach by which that results sharing was handled in the trial, which would have allowed for inclusion of people who opted out or didn't receive results. From an ethical and/or funder perspective, this broader, more inclusive outcome may be at least as important as satisfaction with how results were communicated. | We agree it’s important to take into consideration the extent to which the interventions succeeded in sharing results with those who wanted to know, while not forcing them upon those who did not want to know. As outlined in response to comment 2.7, developing our primary outcome was challenging, and involved substantial patient and public input to ensure it was worded clearly – it would have been difficult to do this clearly for the broader concept you describe (although we agree that such an outcome would be of interest).  While our primary outcome was defined solely among those who reported receiving the results, we say in the analysis section of the methods *“To assess the overall effect of the intervention, it is important to interpret the results of the primary analysis alongside results concerning the possible effect of the interventions on whether participants actually found out the ICON8 results.”*  In the second sentence of the discussion we bring together the results from the primary outcome and reach outcome.  We were fortunate that, for our data, this interpretation is straightforward. The printed summary resulted in higher satisfaction, and people who wanted to know results were more likely to find them out if they were in the group randomised to the printed summary, while no-one who didn’t want the results found them out. Both measures point in the same direction – the printed summary is superior to no printed summary.  We have added discussion of this issue to the first paragraph of the discussion section. | Page 50, lines 875-878 |
| 3.8 | Line 698: It is interesting to consider the importance of the finding that participants made a distinction between features they used vs what they thought others would like to use. This may have implications for how researchers work with participants to design results sharing activities for participants in their own studies. | We agree this is an interesting finding, and it underscores the value of patient and public involvement in designing the form and information to be including in the return of results. It also suggests that researchers should explicitly ask those involved in designing the form and information about what other individuals may wish to use, thinking beyond themselves. Involving a diverse range of contributors who can talk to expectations and preferences of a wide spread of people is important. |  |
| 3.9 | Line 721: The word "interventions" is used here to refer to the ICON8 trial, generating some confusion because "interventions" is mostly used to refer to the interventions in the results sharing trial. The authors might consider rephrasing to improve clarity. | We agree our wording was confusing, and have revised it to “treatment regimens” for ICON8 to distinguish it from communication interventions. | S13 Text |
| 3.10 | Line 827: The conclusion states: "Box 1 contains recommendations based on this research for trial teams, research funders and ethics committees." However, Box 1 contains advice that extends well beyond what was learned from this study. The researchers may consider removing points that are not directly supported by study findings or could make clear that these recommendations are drawn from other sources. Most of Box 1 reports broad suggestions that are not derived directly from the current study's specific findings and are available in existing resources for results sharing that could be cited in the paper rather than being reprinted here in a paper that already includes a great deal of detailed information. | We have revised Box 1 to include only those recommendations directly supported by the study findings presented in this manuscript. | Box 1 |
